# SproutAngio: an open-source bioimage informatics tool for quantitative analysis of sprouting angiogenesis and lumen space

**DOI:** 10.1038/s41598-023-33090-6

**Published:** 2023-05-04

**Authors:** M. Beter, A. Abdollahzadeh, H. H. Pulkkinen, H. Huang, F. Orsenigo, P. U. Magnusson, S. Ylä-Herttuala, J. Tohka, J. P. Laakkonen

**Affiliations:** 1 A.I. Virtanen Institute for Molecular Sciences, University of Eastern Finland, Neulaniementie 2, P.O.Box 1627, 70211 Kuopio, Finland; 2 Department of Immunology, Genetics and Pathology, Uppsala University, Uppsala, Sweden; 3 Vascular Biology Unit, IFOM ETS - The AIRC Institute of Molecular Oncology, Milan, Italy; 4 Heart Center, Kuopio University Hospital, Kuopio, Finland; 5 Gene Therapy Unit, Kuopio University Hospital, Kuopio, Finland

**Keywords:** Angiogenesis, Cardiovascular diseases

## Abstract

Three-dimensional image analyses are required to improve the understanding of the regulation of blood vessel formation and heterogeneity. Currently, quantitation of 3D endothelial structures or vessel branches is often based on 2D projections of the images losing their volumetric information. Here, we developed SproutAngio, a Python-based open-source tool, for fully automated 3D segmentation and analysis of endothelial lumen space and sprout morphology. To test the SproutAngio, we produced a publicly available in vitro fibrin bead assay dataset with a gradually increasing VEGF-A concentration (https://doi.org/10.5281/zenodo.7240927). We demonstrate that our automated segmentation and sprout morphology analysis, including sprout number, length, and nuclei number, outperform the widely used ImageJ plugin. We also show that SproutAngio allows a more detailed and automated analysis of the mouse retinal vasculature in comparison to the commonly used radial expansion measurement. In addition, we provide two novel methods for automated analysis of endothelial lumen space: (1) width measurement from tip, stalk and root segments of the sprouts and (2) paired nuclei distance analysis. We show that these automated methods provided important additional information on the endothelial cell organization in the sprouts. The pipelines and source code of SproutAngio are publicly available (https://doi.org/10.5281/zenodo.7381732).

## Introduction

Angiogenesis is a hallmark of physiological development and is involved in the pathogenesis of many diseases. The most observed type of angiogenesis is sprouting angiogenesis^1^, in which endothelial cells form new capillary branches from pre-existing vessels by interacting with neighboring endothelial cells, extracellular matrix, and other cell types^2^. To date, various cell and animal models have been used to study sprouting angiogenesis, such as in vitro 3D angiogenesis assays based on collagen or fibrin matrix^3^ or mouse retina model^4–6^.

Currently, the most used image analysis parameters to measure vascular structures in angiogenesis models are quantification of vessel/endothelial branch number (sprout number), branch area, branch length, nuclei number, and radial migration/expansion of the vessels^7–9^. Several image analysis tools^10–13^ based on the skeletonization of the endothelial sprouts can be used to measure these parameters. However, multiple limitations for studying vascular structures exist in these tools, such as the usage of segmentation methods based on thresholding that can lead to limited segmentation accuracy. This is especially problematic when the noise and complexity of the vascular network increase making it difficult to find the edges of the objects^14,15^. Another limitation of the current analysis tools is the segmentation of 2D projections rather than 3D images, which results in a loss of valuable information on the 3D morphology of the vessels and the spatial locations of the endothelial cells. Finally, current automated analysis tools including commercial software^16–18^ do not quantitate the empty space within the endothelial cell tube (i.e., lumen) that would bring valuable information on the formation or in part functionality of the vessel. At present, the most used methods for vessel lumen quantitation are manual counting, grading-by-eye, quantifying cross-sections, or simply showing representative cross-section images of the lumens^19–22^. Thus, there is a need for automated lumen quantification to improve the analysis of the vessel structures and better interpretation of sprout/vascular heterogeneity in general.

In this work, we provide a Python-based open-source tool called SproutAngio for segmentation and improved analysis of endothelial and vascular structures. In addition, we provide a fibrin bead assay bioimage dataset for further method development and image analysis studies. SproutAngio improves the accuracy of the automated analysis of endothelial/vascular sprouts and enables automated quantitation of the endothelial lumen space, thus being a useful and valuable tool for the analysis of vascular morphology in various biomedical applications.

## Results

### Description of the confocal microscopy datasets

**Figure 1:**
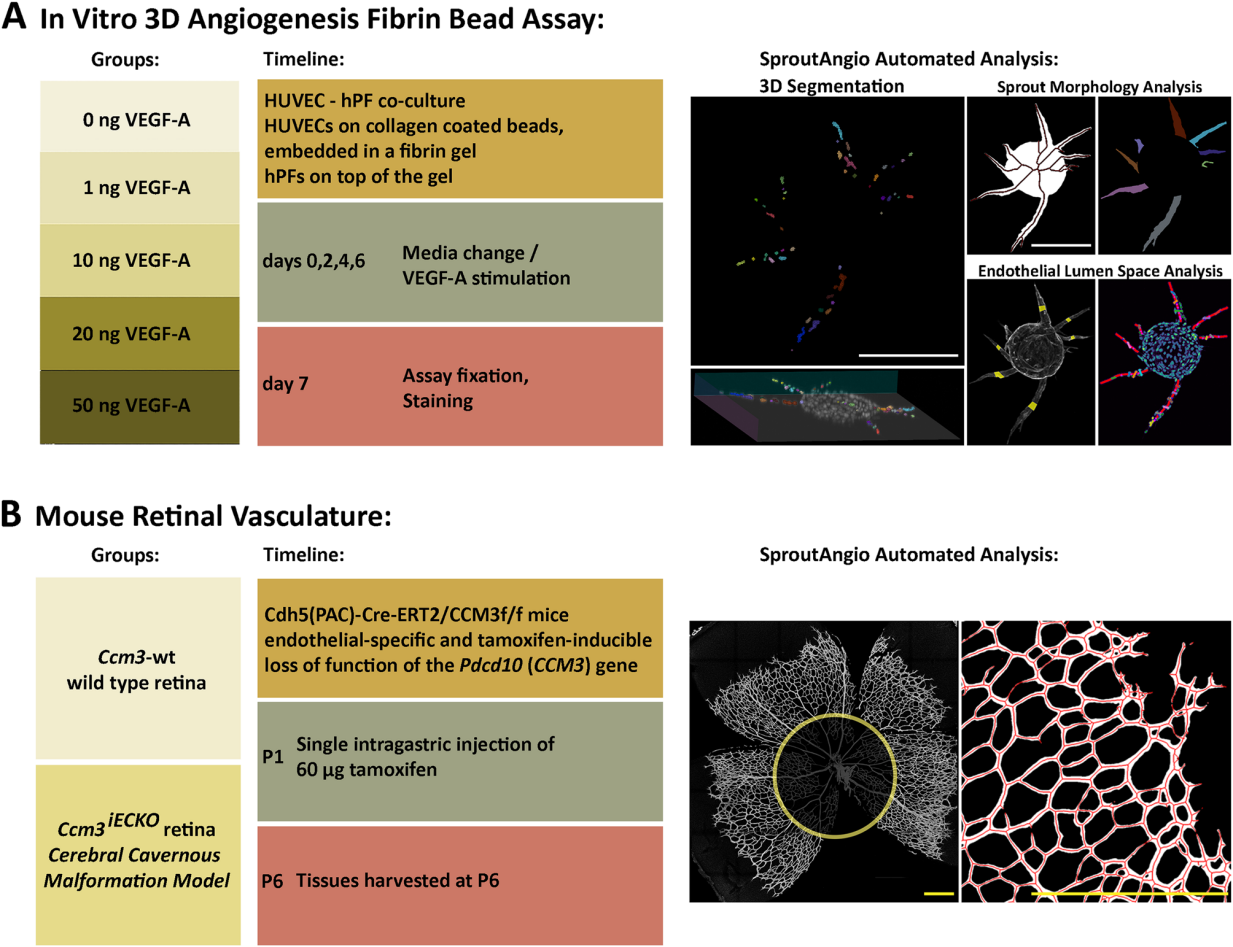
Overview of the datasets used for image analysis by SproutAngio tool. (**A**) In vitro fibrin bead assay was done using five VEGF-A treatment groups (0, 1, 10, 20, 50 ng/ml concentrations) in a 7-day experimental setup. Media change and VEGF-A stimulation were carried out every other day. HUVEC cells were fixed on day 7. 3D segmentation and automated analysis were done using the SproutAngio tool. Regional width analysis and paired nuclei distance analyses were done for the first time, as novel automated methods for the analysis of lumen space. SproutAngio representative images shown from 10 ng/ml VEGF-A treated sample. Scale bars: 200 µm. (**B**) In vivo mouse retinal vasculature model was used to test the image analysis tools in tissue samples. *Ccm3*^*iECKO*^, a mouse model for cerebral cavernous malformation, and control wt mice were used. Branch length analysis was done using the SproutAngio tool. SproutAngio analysis representative images shown from the control *Ccm3* wt sample. P: Postnatal day. Scale bars: 500 µm.

Fibrin bead assay (FBA), an in vitro angiogenesis assay, was used to produce a controlled dataset for analyzing sprouting angiogenesis (Fig. 1A). In this assay, primary HUVECs were put on collagen-coated cytodex beads, embedded in fibrin gel, and fibroblasts were plated on top of the gel^14^. To test our image analysis methods, we produced five groups by adding VEGF-A to the cell growth medium (0, 1, 10, 20, and 50 ng/ml). Media change and VEGF-A stimulation were carried out every other day and HUVEC cells were fixed on day 7. Samples were stained using DAPI for labeling nuclei and phalloidin A-635 for F-actin. Bioimage data was created by using confocal microscopy. 3D segmentation and automated analysis were done using the SproutAngio tool developed in this paper. Regional width analyses and paired nuclei distance analyses were done for the first time, as novel automated methods for the analysis of lumen space by using SproutAngio.

Mouse retina model for a genetic disease, cerebral cavernous malformation (CCM), was used to test the SproutAngio in tissue samples (Fig. 1B). Cdh5(PAC)-Cre-ER^T2^/Ccm3^*f/f*^ mice, we referred here as the *Ccm3*^*iECKO*^ mice, had endothelial-specific and tamoxifen-inducible loss of function of the Pdcd10(Ccm3) gene, as previously described^5^. Tamoxifen was administered to the mice to induce Cre activity and genetic modifications. Mouse pups received a single intragastric injection of 60 μg tamoxifen at P1 (Postnatal day1), and retinas were harvested at P6. CD93 antibody was used to label the vessels. As vein enlargement and hypersprouting near the migration front of the retina was a strong phenotype observed previously in CCM retina model^5^, SproutAngio tool was used to analyze the branches close to the migration front.

### SproutAngio pipeline for automated 3D segmentation and analysis of the endothelial lumen space

**Figure 2:**
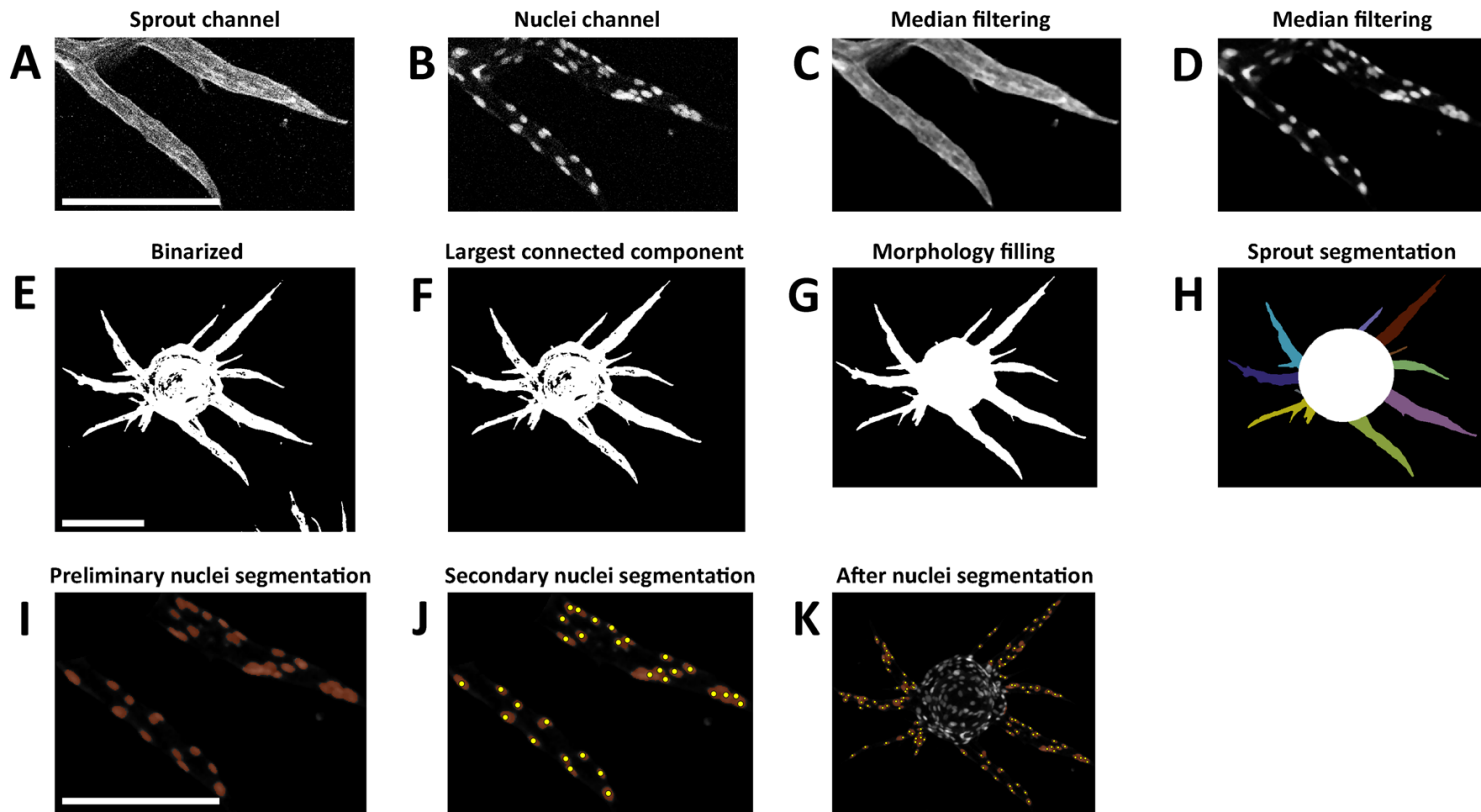
Fibrin bead assay image segmentation steps in SproutAngio tool. (**A**) Zoom-in confocal microscopy image of sprouts (stained with phalloidin A-635), (**B**) Zoom-in confocal microscopy image of nuclei (stained with DAPI), (**C**) 3D median filtered sprout channel using a 3 × 3 × 3 sliding window, (**D**) 3D median filtered nuclei channel, (**E**) Maximum projection of the 3D denoised sprout channels binarized using Otsu’s method, (**F**) Largest connected component after applying a connected components analysis, (**G**) Morphology filling operation applied to close the holes, (**H**) Bead removal and segmentation of the sprouts via superimposing maximum inscribed circles, (**I**) preliminary segmentation of cells via Chan-Vese active contours, (**J**) Secondary segmentation on preliminary under-segmented components, (**K**) Nuclei channel image after secondary segmentation. All SproutAngio representative images are shown from 10 ng/ml VEGF-A treated sample. All scale bars: 200 µm.

We developed an automated pipeline to segment and analyze the morphology of endothelial sprouts and lumen space (Fig. 2). For that, we initially segmented and removed the bead from the FBA images to be able to segment the endothelial sprouts. Then, the cell nuclei were segmented using the Chan-Vese active contours^23^ and further corrected for the under-segmentation errors using the marker-based watershed transform. Next, we analyzed the morphology of the segmented sprouts and the distance between pairs of cell nuclei within each sprout to quantitate the lumen space. Segmentation steps are explained in detail in the methods section.

### Sprout morphology analysis and quantitation of nuclei number

**Figure 3:**
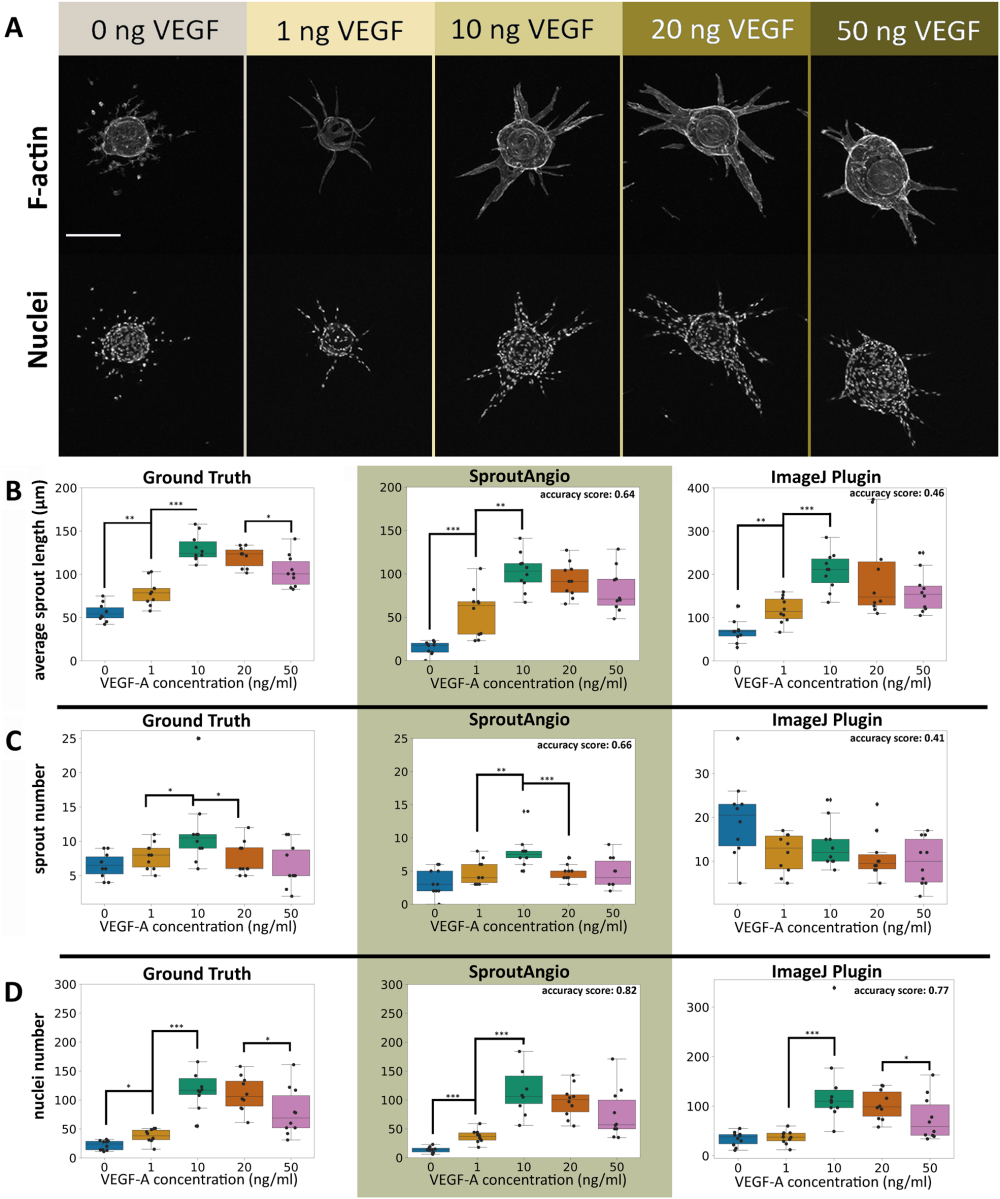
Sprout morphology analysis is improved by SproutAngio tool. (**A**) Representative confocal microscopy images from the fibrin bead assay dataset with gradually increasing VEGF-A concentrations (0–50 ng/ml) are shown in grayscale. F-actin is stained using phalloidin-A635 (upper images), and nuclei with DAPI (lower images). Scale bar: 200 µm. Raw dataset of the confocal microscopy images .czi files are available at: (10.5281/zenodo.7240927). Analysis of (**B**) average sprout length. (**C**) sprout number and (**D**) nuclei number. Ground truth data (left graphs) were created by manually measuring the same parameters from the raw images using the Napari viewer^47^. Automated analyses of SproutAngio (middle graphs) and ImageJ Sprout Morphology plugin (right graphs) were used for comparison. Accuracy scores were calculated by averaging the % group accuracies with 1 being the ground truth. In all groups, n = 10 images/group were used for the analysis. Kruskal–Wallis test was used to determine the statistical significance. *p < 0.05, **p < 0.01, ***p < 0.001.

Representative bead images (Fig. 3A) from each group showed different endothelial sprout morphologies depending on the added VEGF-A concentration. Endothelial cells started sprouting when 1 ng/ml VEGF-A was added to the media and increased the length and thickness of the sprouts gradually when VEGF-A concentration increased to 50 ng/ml. To analyze the sprout morphology by image analysis tools, we used our SproutAngio and an available ImageJ Fiji Sprout Morphology plugin against manually produced ground truth data. For the ground truth data average sprout length, number of branches and nuclei were manually measured from the raw images using Python Napari viewer. SproutAngio results were well in line with the ground truth data in the presence of endothelial sprouts of varied sizes and complexities (Fig. 3B–D). We calculated accuracy scores (Supplementary Fig. 1) for each data point and took an average for each VEGF-A treatment group. Overall accuracy score comparisons (Fig. 3B–D) suggest that our automated analysis by SproutAngio improved all three analysis results compared to the Sprout Morphology plugin. The largest difference in nuclei number and sprout length measurements (ground truth data) were observed between the VEGF-A 1 ng/ml and 10 ng/ml groups (Fig. 3B,D). Accordingly, a significant difference between the same VEGF-A groups was detected using the SproutAngio 3D volumetric analysis (Supplementary Fig. 2).

### Analysis of endothelial lumen space in FBA

**Figure 4:**
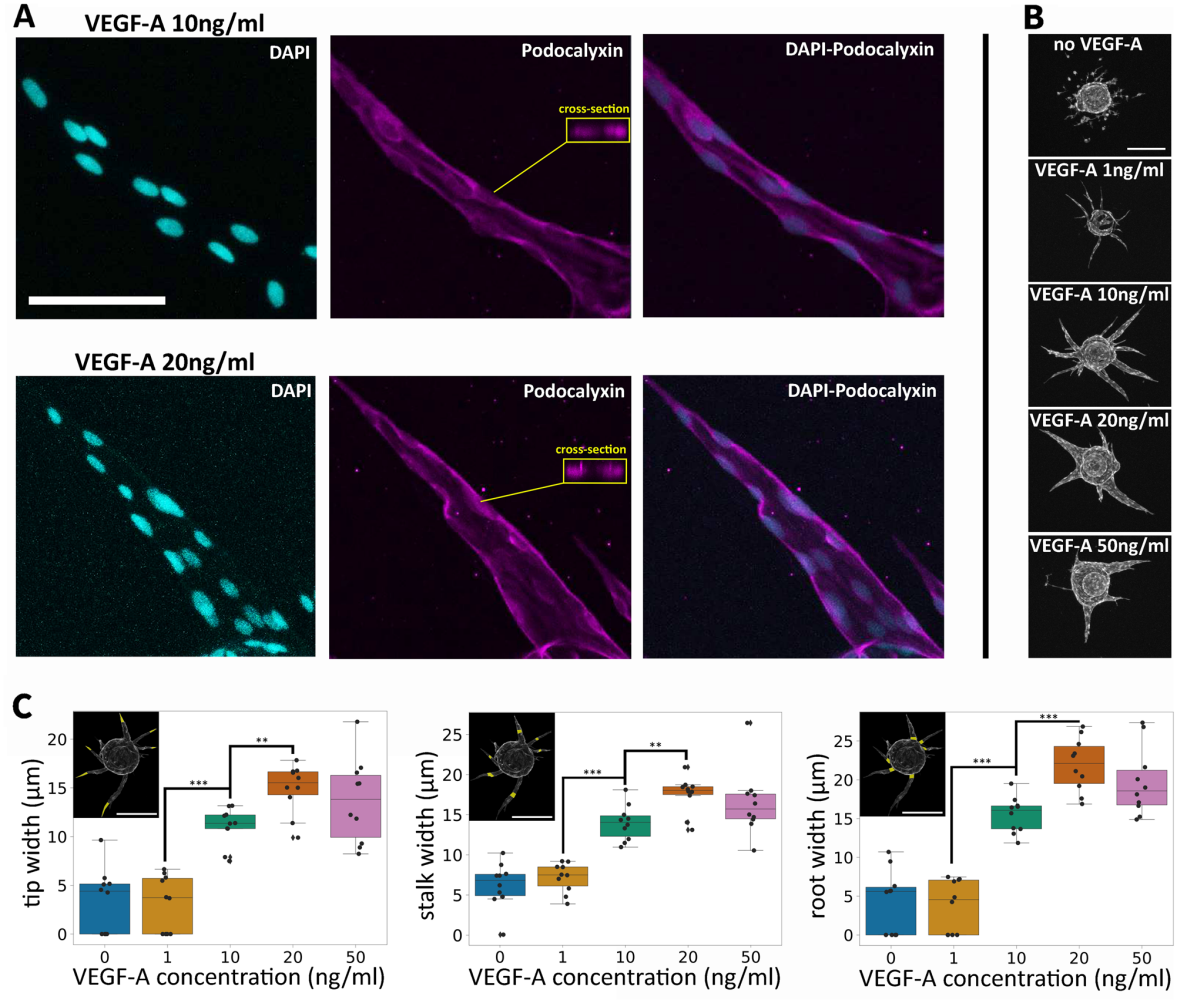
Analysis of endothelial lumen space in fibrin bead assay: width analysis by SproutAngio. (**A**) Nuclei were stained using DAPI (cyan). Cell membrane was immunostained by using podocalyxin (purple). Scale bar: 100 µm. Cross-sections show an empty space in the sprouts, indicating the presence of the lumen. Intensity profiles of cross-sections are shown in Supplementary Fig. 3. (**B**) Representative confocal microscopy images showing the difference in the width between lower and higher VEGF-A concentrations (0, 1, 10, 20, 50 ng/ml). Scale bar: 200 µm. (**C**) Denoting the length of a sprout skeleton as L, we calculated the sprout width by SproutAngio at three locations: tip (0.25 × L), stalk (0.50 × L), and root (0.75 × L). Width measurements from the tip (upper image), stalk (middle image), and root (lower image) segments of the sprouts showing the difference quantitatively between the VEGF-A treatment groups. Representative images in the upper left corners shown from 10 ng/ml VEGF-A treated sample. Scale bars: 200 µm. In graphs, each data point represents the average of an image (n = 10 images/group). Kruskal–Wallis test was used to determine the statistical significance. *p < 0.05, **p < 0.01, ***p < 0.001.

Previously, HUVECs in FBA have been demonstrated to form a lumen space^24^. To confirm the presence of lumen space in our FBA samples we performed podocalyxin immunostaining. Podocalyxin was shown to localize on the cell membrane as expected (Fig. 4A), and intensity profiles of the sprout cross-sections showed the presence of luminal spaces (Supplementary Fig. 3). For the automated analysis of endothelial lumen space by SproutAngio, we used two novel methods: (1) width measurement from different segments of the sprouts and (2) paired nuclei distance analysis. Representative confocal microscopy images show the difference in width between lower and higher VEGF-A concentrations (Fig. 4B). In width analysis using the phalloidin-stained endothelial branches, sprout width was measured from three regions: tip, stalk, and root segments (Fig. 4C). The lowest concentration of VEGF-A (1 ng/ml) did not have a significant difference to sprout width compared to untreated cells. However, starting from 10 ng/ml VEGF-A concentration a gradual increase of the sprout width was observed until a threshold was reached at 20–50 ng/ml concentration of VEGF-A. Comparing the tip, stalk, and root segments; overall width measurements for 10, 20, and 50 ng/ml VEGF-A groups gradually increased from tip to root direction of the sprouts.

**Figure 5:**
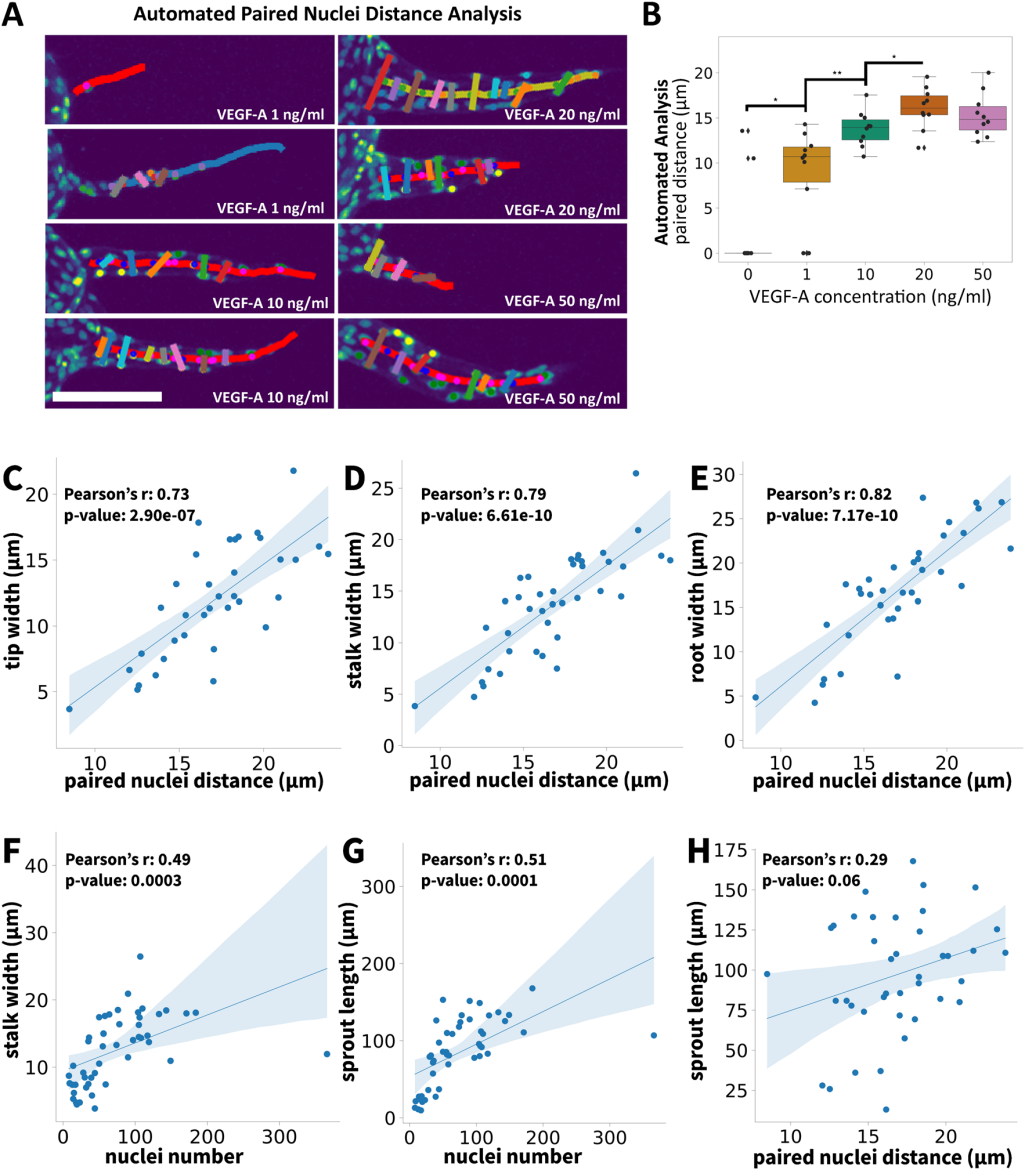
Analysis of endothelial lumen space in fibrin bead assay: paired nuclei distance analysis by SproutAngio. (**A**) Lumen space is determined by measuring the distance between the paired nuclei. The Euclidean distance between the paired nuclei i.e. two nuclei located at opposite sides of the sprout’s curve skeleton that have the minimum distance when projected on the sprout’s curve skeleton is calculated for the analysis. Scale bar: 100 µm. (**B**) SproutAngio automated analysis was consistent with the ground truth data (n = 10 images/group). Kruskal–Wallis test was used to determine the statistical significance: *p < 0.05, **p < 0.01, ***p < 0.001. The correlation analysis between (**C**) tip width—paired nuclei distance, (**D**) stalk width—paired nuclei distance, (**E**) root width—paired nuclei distance, (**F**) stalk width—nuclei number, (**G**) sprout length—nuclei number, (**H**) sprout length—paired nuclei distance was done using Pearson’s correlation. r 0.20–0.39 weak correlation, 0.40–0.59 moderate correlation, 0.60–0.79 strong correlation, 0.80–1.00 very strong correlation.

In paired nuclei distance analysis by SproutAngio, we used the information of nuclei locations in the forming sprouts (Fig. 5). This idea was based on the hypothesized multicellular lumen formation mechanics, in which cell nuclei are positioned towards the sidewalls of the sprout to create the empty lumen space in between the cells^25–27^. After 3D segmentation of the individual sprouts by SproutAngio, nuclei were paired as two nuclei located at opposite sides of the sprout’s curve skeleton that have the minimum distance when projected on the sprout’s curve skeleton (Fig. 5A). The Euclidean distance between the paired nuclei was measured, and an average for each image was taken as a data point (Fig. 5B). We also calculated the accuracy of our automated lumen space analysis by using manual measurements (Supplementary Fig. 4). SproutAngio automated lumen space analysis results were consistent with the manual measurement data suggesting that the 20 ng/ml VEGF-A treatment group had the most enlarged lumen space in comparison to other used VEGF-A concentrations.

To investigate further the differences between basic sprout morphology analysis and lumen space analysis we used Pearson’s correlation analysis. The paired nuclei distance results had strong positive correlations with the sprout width measurements (Fig. 5C–E), indicating that the cells/nuclei were organized to create a lumen space in these regions. Also, as expected, positive correlations were observed between the nuclei number and the stalk width (Fig. 5F), and between the nuclei number and sprout length (Fig. 5G). Instead, no significant correlation was observed between the paired nuclei distance and sprout length (Fig. 5H, Supplementary Fig. 5). The latter is known to be caused by tip cell guiding and stalk cell proliferation, indicating that these parameters do not assess the same biological step in angiogenesis (width vs length) and our paired nuclei distance analysis can provide complementary information of the angiogenesis processes.

### Branch length analysis in the mouse retina

**Figure 6:**
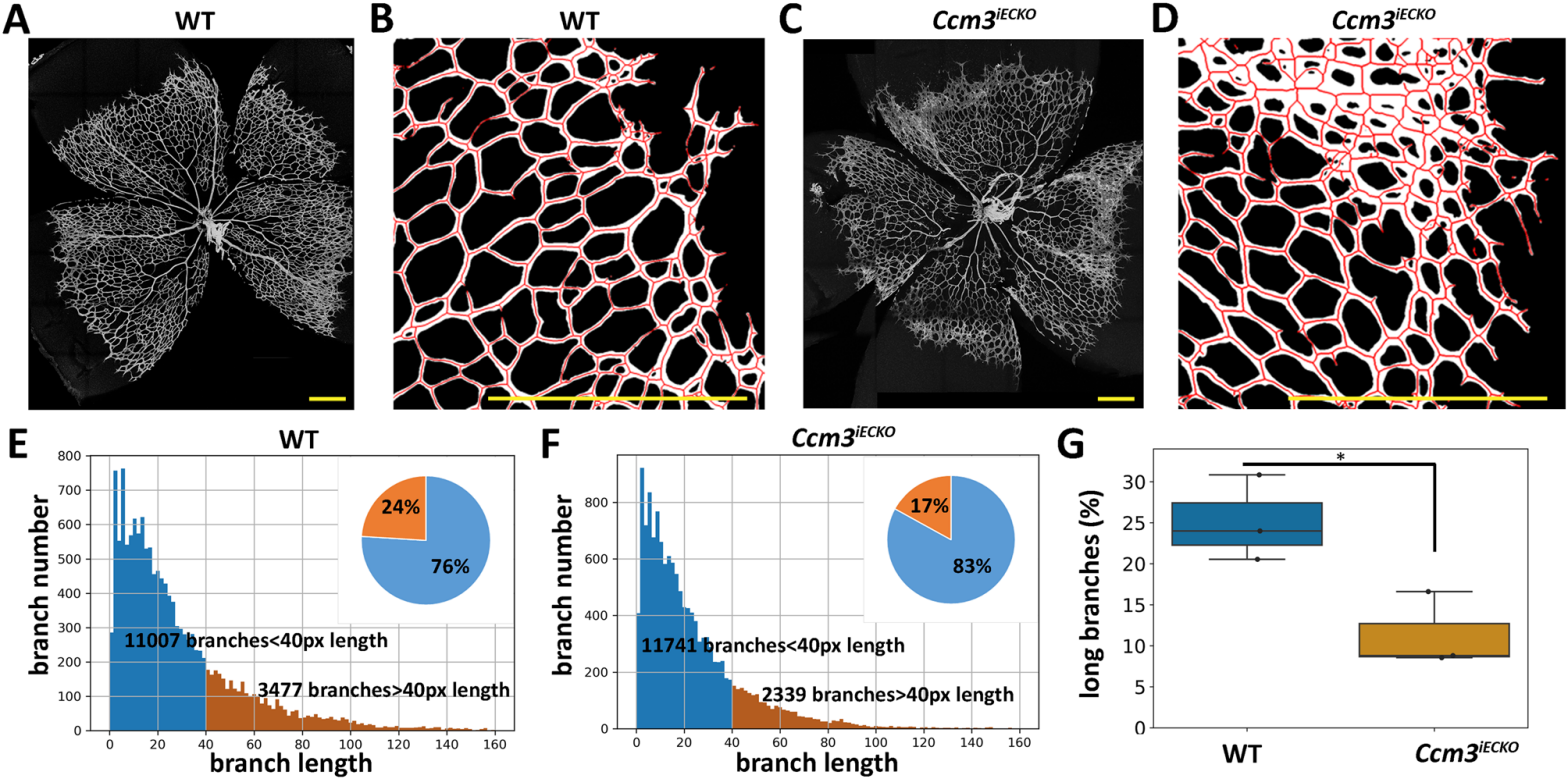
Automated branch length analysis of the mouse retinal vasculature by SproutAngio tool. Retina vessels were visualized with vessel marker CD93. The branches closer to the migration front were analyzed by excluding the central part and the optic nerve of (**A**) wt control and (**C**) *Ccm3*^*iECKO*^ samples (scale bars: 200 µm). (**B**, **D**) Zoom-in images showing the morphological difference of the branches closer to the migration front (scale bars: 500 µm). (**E**, **F**) The histogram x-axis showing the branch lengths in separate bars which were measured by SproutAngio and the y-axis showing the number of branches corresponding to the length values of the individual bars. The percentage of short (blue regions, < 40px [23 μm]) and long (orange regions, ≥ 40px [23 μm]) branches are shown on the pie charts from a representative image. (**G**) Result of the percentage analysis of all samples (n = 3 mice/group) showing a significant difference between *Ccm3*^*iECKO*^ and wild-type retina, while r = 0.5R. t-test was used to determine the statistical significance *, *p* < 0.05.

Next, to show whether our SproutAngio analysis tool works on tissue samples, images of mouse retinal vasculature were used. Morphologically, *Ccm3*^*iECKO*^ retinas displayed abnormal vasculature compared to wild-type retina samples. Vessels in the migration front of the retinas were highly dense and the vascular hierarchy was lost (Fig. 6A–D). To quantify this observation, we analyzed the branches closer to the migration front by excluding the central part and the optic nerve.

**Figure 7:**
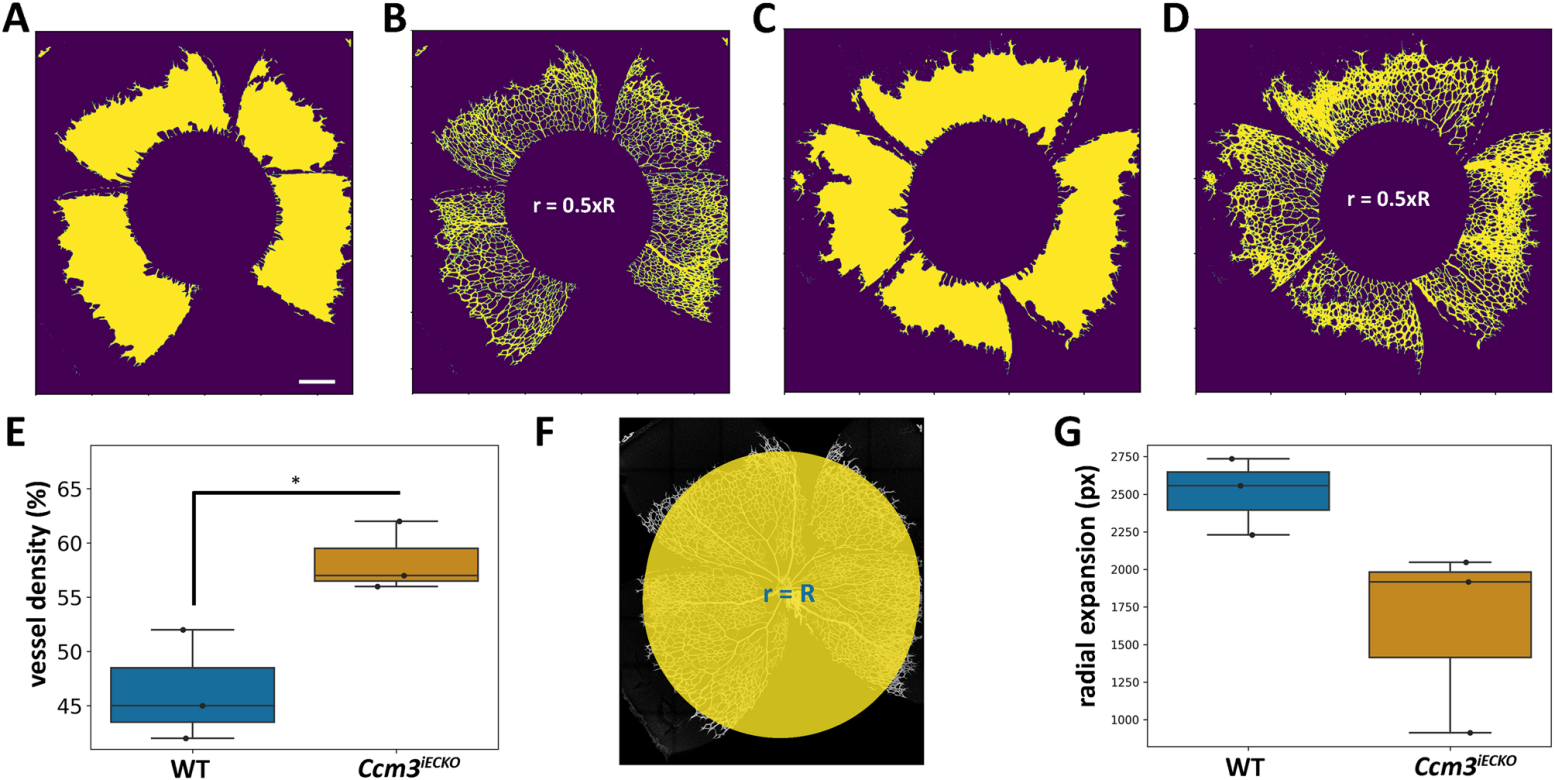
Automated quantification of vessel density, branch number, and radial expansion of the mouse retinal vasculature by SproutAngio tool. Retina vessels were visualized using the vessel marker CD93. The branches closer to the migration front were analyzed from wt control and *Ccm3*^*iECKO*^ samples by excluding the central part and the optic nerve. (**A**, **C**) A morphology-filling operation to close the holes in segmented branch images was applied to calculate the vessel density (total area). Vessel density was calculated automatically by dividing the segmented vessel area (**B**, **D**) by the total area. (**E**) Vessel density was significantly different between *Ccm3*^*iECKO*^ and wild-type retina (n = 3 mice/group). (**F**) The radial expansion was calculated automatically by SproutAngio by measuring the diameter (R) of the largest maximum inscribed circle inside the segmented retina area. (**G**) Radial expansion measurement showed no significant difference between the groups (n = 3 mice/group). In all images, t-test was used to determine the statistical significance *, *p* < 0.05. Scale bar: 200 µm.

To exclude the central area as in FBA bead exclusion, we defined radius R as the maximum of the Euclidean distance transform and used r = a × R where “a” is a user-defined threshold in SproutAngio. To test the best option, we used a = 0.5 (Fig. 6), a = 0.3, and a = 0.7 (Supplementary Fig. 6) in our analyses. After the skeletonization step (Fig. 6B,D), we analyzed the skeleton structures with SproutAngio by measuring skeleton length and branch number, using the open-source Python library Skan^28^ (Fig. 6E,F). We further used a user-defined threshold of 40px (23 μm) for vessel branch length, to define the long branches. As a result, the *Ccm3*^*iECKO*^ group had a significantly lower percentage of long branches compared to the control at P6 (Fig. 6G). Comparable results were obtained when a = 0.3 or a = 0.7 was used (Supplementary Fig. 6), whereas no significant difference was observed in the total number of branches (n = 3 mice/group). Vascular density and radial expansion parameters, widely used for retinal vasculature analysis, were also integrated into the automated vascular analysis of SproutAngio. Vascular density was shown to be significantly increased for the *Ccm3*^*iECKO*^ group (Fig. 7A–E). ImageJ Fiji Vascular Analysis plugin was also used for comparison, which similarly showed an increase for the Ccm3^iECKO^ however not significant (Supplementary Fig. 7). The radial expansion was reduced for the *Ccm3*^*iECKO*^ group; however, it was also not statistically significant (n = 3 mice/group; Fig. 7F,G). For validation, we also used manual measurement of radial expansion showing similar results (Supplementary Fig. 7). Altogether, these data demonstrate that SproutAngio can be used for detailed and automated analysis of retinal vasculature with ease of modifying the analysis parameters.

## Discussion

We developed SproutAngio, a Python-based open-source tool for automated analysis of endothelial lumen space and sprout morphology. SproutAngio improved the automated segmentation method used for analysis of the sprout morphology and outperformed a widely used ImageJ plugin. In SproutAngio tool, we also provide two novel methods for the automated analysis of endothelial lumen space: width analysis from different segments of sprouts and paired nuclei distance analysis. In addition to providing methods for in vitro 3D assay, we also show the usability of SproutAngio to analyze the branching of the retinal vasculature.

Various 3D cell models have been developed to study vasculature in vitro^29,30^, however, the analysis of endothelial structures has been performed mainly from 2D projections. Here, we used automated 3D segmentation to analyze the endothelial sprout morphology. Comparison of the accuracy scores of SproutAngio and ImageJ Sprout Morphology plugin^12^ against the manually produced ground truth data showed that SproutAngio outperformed the ImageJ plugin in analyzing sprout length/number, and nuclei number in the presence of endothelial structures of varied sizes and complexities. Segmentation methods provided in SproutAngio enabled both the analysis of nuclei locations and the sprout volume of 3D images of the FBA. In addition, sprout volume images showed that angiogenic sprouts developed in both the horizontal and the vertical planes. Therefore, using SproutAngio’s 3D segmentation provides more precise information compared to 2D analysis tools such as the widely used AngiogenesisAnalyzer^13^. SproutAngio also allows more precise bead detection, including the proliferating ECs around the beads often observed in FBA experiments after days 5–6. These ECs do not form sprouts but proliferate and enlarge the central bead region complicating the sprout analysis as in 50 ng VEGF-A treatment samples in our dataset. In this case, the central bead region does not have a perfect spherical shape and correct segmentation of the tip cell guided sprouts is dependent on accurate detection of the bead area.

So far analysis of lumen space in 3D has been rare due to the usage of 2D segmentation, and tedious immunolabeling protocols for detecting the empty lumen space in vessels, not suitable for all samples^20^. Here, we used SproutAngio to measure lumen width from 3D segmented images by either analyzing the sprout width in various segments along the endothelial sprout or by measuring the paired nuclei distance in the sprout. Both methods gave comparable results, indicating that these methods can be used for automated lumen analysis. Importantly, SproutAngio lumen space analysis methods provided also additional information that we could not measure using the basic sprout morphology methods. As an example, the 10 ng/ml VEGF-A group gave the maximum angiogenic response having the highest endothelial sprout length, sprout number, and nuclei number. However, 20 ng/ml VEGF-A group significantly increased the endothelial lumen space compared to 10 ng/ml VEGF-A group, suggesting that our methods provided additional information about the endothelial tube formation in the presence of growth factor stimulation. Further investigation of the correlation between the measured parameters by SproutAngio indicated a higher cellular organization in terms of nuclei positioning on the sides of the wider endothelial sprouts. Regional width results from sprout segments were strongly correlated with the paired nuclei distance results, whereas sprout length had no role. It is important to note that in our analysis all the segments were chosen based on pixel length only and thus did not represent the true tip cell area, and thus this segment likely also consists of stalk cells, a specialized endothelial cell phenotype responsible for lumen formation^31,32^. A recent computational study by Soares et al.^33^ suggested that the increase in proliferation of stalk cells triggered by VEGF-A did not necessarily increase the vessel thickness, but the amount of vessel branches. Supporting this hypothesis, our results indicate that under a growth factor stimulation, sprout width is less dependent on the overall increase in nuclei and sprout numbers and more dependent on the cellular organization.

Both paired nuclei distance analysis and regional width analysis methods provide alternative ways to quantify lumen space from multiple segments of sprouts. These measurements from longitudinal vessels in 2D/3D images likely give more reliable results compared to lumen area analysis from cross-section images. In our dataset, both methods also provided additional information about the organization of the nuclei in sprouts. For example, there was no significant difference between untreated and 1 ng/ml VEGF-A treatment groups in sprout width. However, by paired nuclei distance analysis, a significant difference between these groups was observed showing the increased nuclei organization already with 1 ng/ml of VEGF-A.

Besides endothelial sprouts from in vitro bioimage dataset*,* we also analyzed the vascular branch length in the retina of *Ccm3*^*iECKO*^ mice. The defective vascular sprouting of the *Ccm3*^*iECKO*^ group has previously been observed^5^. In the earlier image analysis, the radial expansion of the vascular network was measured manually as the mean distance covered by the vessels growing from the optic nerve. Accordingly, we show here similar defective sprouting, i.e., reduced branch length, in the *Ccm3*^*iECKO*^ group by using an independent dataset (at P6). Moreover, we reasoned that the observed reduction in the branch length should reflect an increased vascular density and a reduced radial expansion of the vascular network, which are two widely used parameters for retinal vasculature analysis. Therefore, we included these parameters in the SproutAngio analysis pipeline and similarly found an increased vascular density in the *Ccm3*^*iECKO*^ group. We also used ImageJ Fiji Vascular Analysis plugin for comparison. Vascular Analysis plugin results also showed an increased vascular density in the *Ccm3*^*iECKO*^ group, although not statistically significant. In the radial expansion of the vascular network, we measured a reduction, also not statistically significant. We confirmed this result by manually measuring the radial expansion as well. The lack of statistical validation might be due to the small number of animals analyzed in our dataset or the age of the mice in comparison to the previous publication (P6 vs. P8)^5^. However, statistical significance in SproutAngio vessel density results compared to the Vascular Analysis plugin also shows that the exclusion of user-defined central region in SproutAngio analysis can provide important information for analyzing the migration front in the retinal vasculature.

SproutAngio tool has limitations in the current version related to nuclei pairing, area filters and microscopy imaging. These method limitations are discussed under the materials and methods section.

In conclusion, we demonstrate here a new image analysis tool SproutAngio for a reliable and automated quantitation of angiogenic sprouts and vasculature. We tested our tool using both in vitro and in vivo confocal microscopy datasets. The source code for SproutAngio (https://doi.org/10.5281/zenodo.7381732, under MIT license), and in vitro confocal microscopy dataset (https://doi.org/10.5281/zenodo.7240927) are publicly available.

## Materials and methods

### Cells

Human umbilical vein endothelial cells (HUVEC) were isolated as previously described^34^ using collagenase 3 mg/10 ml concentration and maintained in PromoCell Endothelial Cell Growth Medium (Heidelberg, Germany) with 1× endothelial growth kit addition and 10%FBS. HUVECs were used in passage 2. Human lung primary fibroblasts (hPF) were purchased from PromoCell and maintained in DMEM with 10% FBS. hPFs were used at passage 8. Collection of umbilical cords for cell isolation was approved by Ethics Committee of the Kuopio University Hospital (Kuopio, Finland, 341/2015).

### Fibrin bead assay

A 3D in vitro model (FBA) mimicking angiogenesis was performed as described previously^13–15^ with additional optimizations. Briefly, Cytodex 3 beads (GE Healthcare, Little Chalfont, UK) were hydrated 3 h in PBS and autoclaved at 121 °C for 16 min. HUVECs were seeded on top of collagen coated Cytodex 3 beads -2500 beads with one million HUVECs in 1.5 ml final volume for the coating with multiple washing steps to get rid of non-attached cells. Incubation for coating takes 4 h with gentle inversion of tubes every 20 min for confluent attachment of HUVECs. After that, an overnight incubation in T25 flask with additional 5 ml media follows. Next day, 25 mg/ml fibrinogen in DPBS at 37 °C, with additional 0.15 U/ml aprotinin was prepared for embedding step (fibrinogen, aprotinin, thrombin; Merck KGaA, Darmstadt, Germany). After several washing steps using DPBS, HUVEC coated beads were resuspended in fibrinogen solution ~ 300 beads/1 ml fibrinogen. 3 µl thrombin (10 U/ml) was pipetted at the center of each well and then mixed with 250 µl fibrinogen-bead mixture pipetting up/down 5–6 times for each well of a 48-well-plate. Since the fibrinogen—thrombin mixture solidifies fast, it is important to avoid extra movement and change pipette tips after each well. After 5 min room temperature and 15 min 37** °C** incubation, hPFs were cultured on top of the fibrin gel -10,000 cells/well in 48-well-plate. Cell culture was maintained with EBM media with additives (Lonza, Basel, Switzerland), and stimulated with 0 ng/ml, 1 ng/ml, 10 ng/ml, 20 ng/ml or 50 ng/ml VEGF-A (R&D Systems, Minneapolis, MN). Medium change and VEGF-A stimulation were done every other day during the 7-day follow-up^15^. Media change was done dropwise to avoid damaging the gel.

On day 7, fibroblasts were removed using 0.25% trypsin and after a washing step using 1× DPBS, HUVECs were fixed in 4% PFA for 10 min. Then, after another PBS washing step, cells were treated with methanol (− 20 °C) for 5 min. After the fixation, cells were permeabilized with 0.5% triton in PBS for 5 min and then stored in DPBS in + 4 °C until the immunostaining procedure.

For immunostaining, the cells were treated with 3% triton in PBS for 15 min. Phalloidin-A635 (Thermo Fisher Scientific, 1:40) staining was performed in 3% triton-PBS solution for 90 min to label F-actin. After three 15 min washing steps with 1X DPBS, nuclei were stained with DAPI (1:1000, Sigma-Aldrich, St. Louis, MO) 5 µg/ml in 3% triton-PBS solution for 10 min and washed with DPBS. The cells were stored in DPBS at + 4 °C prior to imaging.

### Murine model

*Cdh5(PAC)-Cre-ER*^*T2*^*/Ccm3*^*f/f*^ mice were used for the analysis of mouse retinal vasculature. *Ccm3*^*f/f*^ mice exons 4–5 of the *Pdcd10 (Ccm3)* gene flanked by loxP sites were bred with *Cdh5(PAC)-Cre-ER*^*T2*^ mice to obtain endothelial-specific and tamoxifen-inducible loss of function of the *Pdcd10 (Ccm3)* gene, as previously described^5^. The *Cdh5(PAC)-Cre-ER*^*T2*^ mouse line was kindly provided by R.H.Adams (Department of Tissue Morphogenesis, Faculty of Medicine, Max Planck Institute for Molecular Biomedicine University of Münster, Germany). The genotype of the *Ccm3*^*iECKO*^ mice were *Ccm3*^*f/f*^, *Cdh5(PAC)-Cre-ER*^*T2*^+ (referred to in the results as *Ccm3*^*iECKO*^). The genotype controlled the selection of mice used for the study and the genotype of *Pdcd10 (Ccm3)* wild-type mice were *Ccm3*^*f/f*^*; Cdh5(PAC)-Cre-ER*^*T2*^− (referred to in the results as wild-type). Comparison of *Ccm3*^*iECKO*^ mice (n = 3) with wild-type mice (n = 3) was performed between litter mates. The mice were maintained in the barrier facility of the Rudbeck Laboratory (Uppsala University, Sweden) and were screened for the specific pathogens according to the guidelines of the Federation of European Animal Laboratory Science Associations. All of the mice were housed in microisolator cages that contained wood shavings and enrichment and were kept in a climate-controlled environment with 12-h light/12-h dark cycles. They were fed standard rodent chow with free access to water and regularly monitored for health status. No adverse events were observed.

Tamoxifen was dissolved in 10% ethanol-corn oil (10 mg/ml) and administered to the mice to induce Cre activity and genetic modifications. Mouse pups received a single intragastric injection of 60 μg tamoxifen at P1 performed by an experienced animal technician, and the tissues were harvested at P6. This mouse model provides *Ccm3*^*iECKO*^ mice that develop lesions in the retina and in the brain. This model, *Ccm3*^*iECKO*^ can be kept until P10 as mice are viable and healthy mice, despite lesions in the retina and the brain^35^. The health status of the pups was controlled on a daily basis.

The mouse eyes were collected and fixed in 4% PFA in PBS for 2 h at 4 °C. Retinas were dissected and incubated at 4 °C overnight with primary antibodies diluted in PBSTC buffer (PBS with 0.5% Triton-X-100, 0.1 mM CaCl_2_) supplemented with 5% donkey serum, followed by the suitable species-specific Alexa Fluor-conjugated secondary antibody staining, overnight at + 4 °C. For vessel staining anti-CD93 (R&D Systems, AF1696) antibody was used.

No randomization was used for this animal study. The animal technician handled the breeding and tamoxifen delivery to mice and tissue collections unaware of future group allocations in the experiment. The *Ccm3*^*iECKO*^ mice have a clear phenotype that is very different from the wild-type control mice and three mice per group determined the sample size. Both female and male pups were used for this study. In line with the guidelines of the National Centre for the Replacement, Refinement and Reduction of Animals in Research (3Rs, London, UK) we were able to reduce the number of animals by optimizing the tissue preparations upon collection.

All experiments involving animal studies were conducted according to the principles in the Swedish National Board for Laboratory Animals and European Convention for Animal Care. Animal experiments were approved by the regional ethics committees in Uppsala, Sweden (permit number 5.8.18-16224/2020).

A protocol registration for animal breeding was performed on a routine basis using the Software program FileMaker for documentation. Animal statistics are reported on a yearly basis to the Swedish Board of Agriculture, Jönköping, Sweden. Animal procedures were performed in accordance with the ARRIVE Guidelines (https://arriveguidelines.org/).

### Image acquisition

FBA plates were imaged with a Zeiss LSM800 confocal laser scanning microscope using 10×/0.3 PlanApo objective. DAPI was detected using 353/465 nm excitation/emission wavelengths, phalloidin was detected using 631/648 nm wavelengths. The frame size:1024 × 1024 pixels, z-dimension step size: 5 µm, and averaging of 2 were used.

Retina samples were imaged on Leica confocal SP8 using a 20× objective. Laser line 405, 653 was used for the detection of images. The total retina z-stack was 30 µm with 1 µm, step size, having a frame size of 1024 × 1024 pixels.

### FBA image analysis by ImageJ Sprout Morphology plugin and generation of the ground truth data

The confocal microscopy images were analyzed by ImageJ Sprout Morphology plugin, as described^12^. We set the thresholds experimentally evaluating the performance of the tool, using 2 images. We used the same threshold values for all images. Ground truth data for average sprout length, number of branches and nuclei were manually measured from the raw images using Napari viewer.

### FBA image segmentation by SproutAngio tool

The confocal microscopy images were separated into the sprouts (Fig. 2A, stained with phalloidin A-635) and nuclei channels (Fig. 2B, stained with DAPI). We applied a 3D median filter using a 7 × 7 × 7 sliding window to denoise each channel separately, as shown in Fig. 2C,D. It should be noted that, because an FBA is a 3D co-culture model, the presence of gel causes noisier images compared to a mono-layer in vitro model.

#### Sprout segmentation

The maximum projection image of the 3D denoised sprout channel was generated and then binarized with Otsu’s method^36^ (Fig. 2E). We applied a connected components analysis to the binarized image and kept the largest connected component (Fig. 2F) as the initial step in segmenting the sprouts in FBA images; we denote this image as B. To segment the central bead, first, we applied a morphology filling operation to close the holes in B (Fig. 2G). Then, we found the largest maximum inscribed circle inside *B* by applying the Euclidean distance transform^37^ on *B* (the distance transform of a binary image labels each pixel of the image with the distance to the nearest boundary pixel in a binary image). We define radius *R* as the maximum of the Euclidean distance transform. The location at which the maximum occurs is the locus of the largest maximum inscribed circle, or bead, inside *B*. As the sprouts grow around the bead, removing the detected central bead from *B* leads to the segmentation of the sprouts. We ensured that bead removal leads to the segmentation of sprouts by enlarging the detected bead via superimposing maximum inscribed circles with radii bigger than *aR*, where *a* is a user defined threshold (Fig. 2H).

#### Nucleus segmentation

We developed an automated nucleus counting technique based on a preliminary foreground segmentation and a subsequent correction of the under-segmentation error. For the preliminary cell segmentation, Chan-Vese active contours^23^ were applied to the median filtered nuclei channel images, as implemented in Python Scikit-Image^38,39^. The Chan-Vese model was initialized with a binary mask and the speed function was the gradient of the nuclei channel. The smooth factor equal was set to 0.25, and the maximum number of iterations was set to 500 to perform the evolution of the segmentation. The initial contours were deformed on the speed function to adapt to the shape of cells, resulting in the preliminary segmentation of cells (Fig. 2I). The preliminary segmentation, however, contained under-segmented components (nuclei touching each other appear in the same connected component) and very small noise components. Thus, we measured the area of every preliminary segmented nucleus. If the area was smaller than a threshold S = 30 pixels [25 μm^2^] the component was discarded, and if the area was greater than a threshold G = 400 pixels [336 μm^2^] the component was recognized as an under-segmented component. The preliminary segmented components with the area in [S,G] were correctly segmented, requiring no further analysis. A secondary segmentation was run (Fig. 2J,K) on every preliminary under-segmented component using the marker-based watershed transform. Within the domain of an under-segmented component, we defined the regional maxima of the H-maxima transform of the filtered image as markers. A set of conditions were applied to the extracted markers: (1) the value of a marker on the intensity image should be greater than a threshold I = 0.1; (2) the spatial distance between two markers should be greater than the average of correctly segmented nucleus diameter. Determining the set of markers, the watershed transform was applied to finalize the cell segmentation. We set the thresholds experimentally evaluating the performance of the automated nuclei segmentation compared to 2 images in which all cells were counted by an expert (MB). We used the same threshold values for all images.

#### Endothelial lumen space analysis

The width of each segmented sprout was measured by first defining its skeleton using an approach previously described^40,41^, which remains in the center of the sprout. Then, we applied Euclidean distance transform to a sprout segment; the value of the distance transform at each point on the sprout skeleton is half the sprout width at that point^42^. Denoting the length of a sprout skeleton as L, we calculated the sprout width at three locations: tip (0.25 × L), stalk (0.50 × L), and root (0.75 × L). For short length sprouts, when L is smaller than [25 μm] 30 pixels, we only measured the stalk width and excluded the tip and root measurements. We used another method to measure the lumen space which is to measure the distance between paired nuclei. Paired nuclei were defined as two nuclei located at opposite sides of the sprout’s curve skeleton that have the minimum distance when projected on the sprout’s curve skeleton. This means that, within each sprout, we first identified at which side of the sprout’s skeleton curve a nucleus was located—the sprout’s skeleton curve partitions the sprout’s domain into two. The nuclei were projected to the sprout’s curve skeleton and iteratively paired two nuclei that had the shortest distance between them. After pairing the nuclei, the Euclidean distance between the pairs was calculated.

### Mouse retinal vasculature image segmentation by SproutAngio tool

The FBA pipeline was adapted to segment and analyze the morphology of the retinal vasculature of confocal microscopy images. The retina images were visualized with vessel marker CD93. A median filter using a 6 × 6 sliding window was applied to denoise the image. To exclude the central area, we found the largest maximum inscribed circle inside the image by applying the Euclidean distance transform as in fibrin bead assay segmentation. We defined radius *R* as the maximum of the Euclidean distance transform and used *aR*, where a is a user-defined threshold, we used *a* = 0.5 in our experiment. Then, we analyzed the skeleton structures by measuring the skeleton length and branch number, using the open-source Python library Skan. Skan’s output was previously compared to Fiji’s Analyze Skeleton plugin by Nunez-Iglesias et al.^28^. To quantify the vascular density, we first applied a morphology-filling operation to close the holes in the vessel branch area. Then after dividing the area of the branches by the morphology-filled total area, we multiplied the result by 100 to find the vessel density percentage for each group. For measuring the radial expansion of the retinal vasculature, we used the radius of the maximum inscribed circle (R) as the average radial expansion value, the mean distance covered by the vessels growing from the optic nerve.

### Statistical analysis

For the analysis of the FBA datasets, we compared neighboring groups using the Kruskal–Wallis test if Shapiro–Wilk test rejected the null-hypothesis of normality with the alpha-level 0.05. Pearson’s correlation was used for the correlation analysis. The absolute values of r 0–0.19 were regarded as very weak, 0.2–0.39 as weak, 0.4–0.59 as moderate, 0.6–0.79 as strong, 0.8–1 as very strong correlation. *p < 0.05, **p < 0.01, ***p < 0.001 were used to show the statistical significance of the figures. Our statistical analysis and graph drawing algorithms are available at SproutAngio repository. Accuracy for Supplementary Fig. 1 was calculated by 1-%error and average was used as accuracy score in Fig. 3. For retina analysis of vessel density and branch length results, t-test was used.

### Limitations of SproutAngio tool

Paired nuclei distance analysis uses data from both sprout segmentation and nuclei segmentation. When the complexity of sprouting increases, if the sprout segmentation does not separate the sprouts correctly, there is a higher chance of wrong pairing of the nuclei. It is also possible that nuclei from different, adjacent sprouts pair wrongly affect the data analysis. In this case, the user needs to manually omit those data points looking at the resulting images.

In nuclei segmentation, area filters are used to define individual nuclei to increase the accuracy of the segmentation. Users can change these filter parameters based on their samples to improve the segmentation. However, these filters might cause missing data points in nuclei segmentation part. In our paired distance results, when compared with the ground truth, SproutAngio automated analysis results had slight overestimations. Area filters could be a reason for this observation. Also, for smaller width sprouts, which consists mostly of single line nuclei on the sprouts, such as 1 ng/ml VEGF-A treatment in our dataset, there are fewer nuclei to pair. This decrease in paired nuclei sample, increases the error for the automated paired distance measurements.

For FBA dataset, microscopy images containing only one bead per image is optimal. Because for the automated analysis, our pipeline needs to detect the largest connected component in the image. If there is more than one bead or big enough parts of other beads, the tool might fail to detect the correct object. However, smaller nonconnected parts do not cause this problem.

## Supplementary Information

Supplementary Information 1.

Supplementary Information 2.

## Data Availability

In vitro data used in the present analyses are publicly available on https://doi.org/10.5281/zenodo.7240927https://zenodo.org/record/7240927 and in vivo data supporting the conclusions of this manuscript will be made available by the corresponding author, upon reasonable request, to any qualified researcher.

## Code availability

SproutAngio code used for data processing, analysis and figure generation directly relies on the following open-source Python packages: astropy^43^, czifile^44^, fil-finder^45^, matplotlib^46^, napari^47^, numpy^48^, scikit-image^49^, scipy^50^, seaborn^51^. Additional software used in the reported analyses: ImageJ Fiji^10,11^, Sprout Morphology Analysis plugin^12^. The source code for SproutAngio is publicly available and is provided under MIT License. https://doi.org/10.5281/zenodo.7381732 (https://zenodo.org/record/7381732).
