# Supplementary material for: SproutAngio: an open-source bioimage informatics tool for quantitative analysis of sprouting angiogenesis and lumen space
Source: Sci Rep. 2023 May 4;13:7279. doi: 10.1038/s41598-023-33090-6 (PMC10160097; doi:10.1038/s41598-023-33090-6)
Supplement: Supplementary file 1 — Supplementary Information 1. [file 41598_2023_33090_MOESM1_ESM.docx]

**SproutAngio: An Open-Source Bioimage Informatics Tool for Quantitative Analysis of Sprouting Angiogenesis and Lumen Space**

**Authors:** Beter M., Abdollahzadeh A., Pulkkinen HH, Huang H., Orsenigo F., Magnusson PU, Ylä-Herttuala S., Tohka J., Laakkonen JP.

**SUPPLEMENTARY FIGURES**


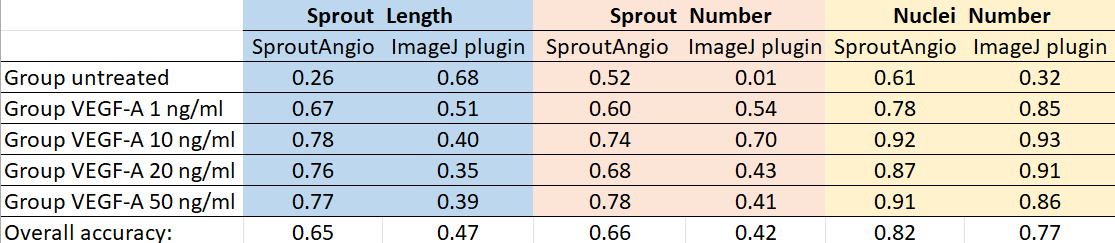


**Supplementary Figure 1**: The comparison of accuracy scores between our automated analysis results and ImageJ Sprout Morphology plugin results for each VEGF-A treatment group (0-50 ng/ml) in fibrin bead assay. Accuracy scores were calculated using manual measurements. Scale 0-1, 1 being the ground truth.


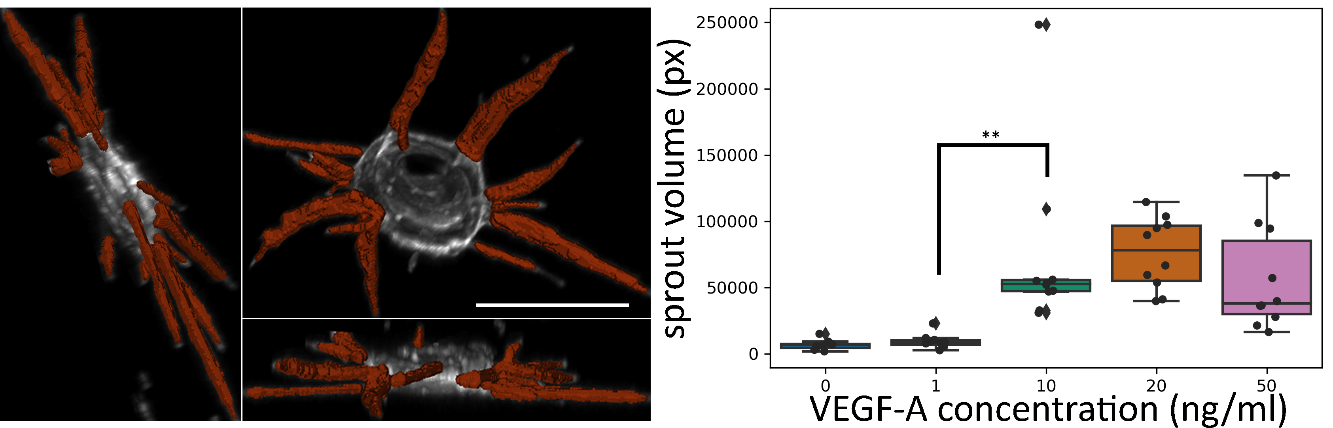


**Supplementary Figure 2**: Representative image showing the 3D segmentation of sprouts by SproutAngio, scale bar: 200 µm (left figure). Sprout volume measurements from the fibrin bead assay confocal microscopy images are shown on the right for each VEGF-A treatment group (0-50 ng/ml). Each treatment group has 10-x images. Kruskal-Wallis test was used to determine the statistical significance. ** p<0.01.


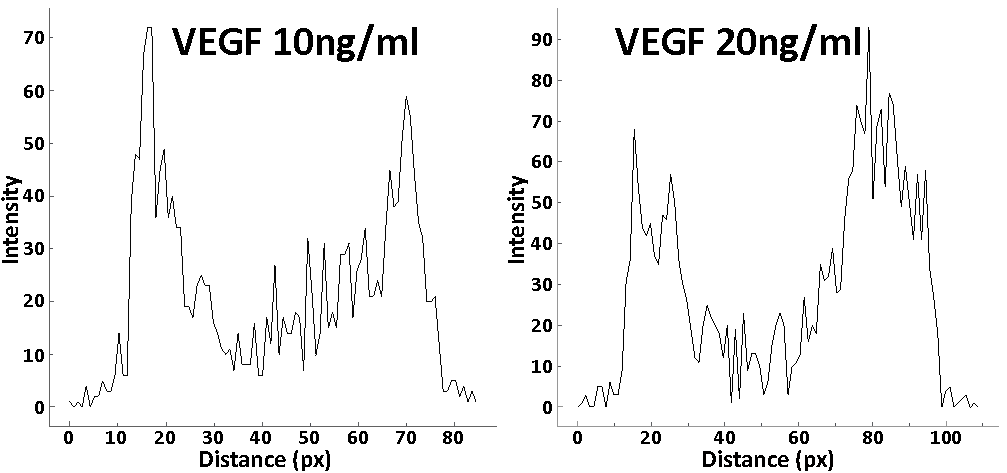


**Supplementary Figure 3:** Representative intensity profiles from cross-sections of 10ng/ml VEGF-A and 20ng/ml VEGF-A samples used in Fig. 3A. The cell membrane was labelled using podocalyxin. The lower intensities are detected in the middle of the sprouts, implicating the presence of the endothelial lumen. The intensity profiles were measured by the Napari-Plot-Profile plugin.


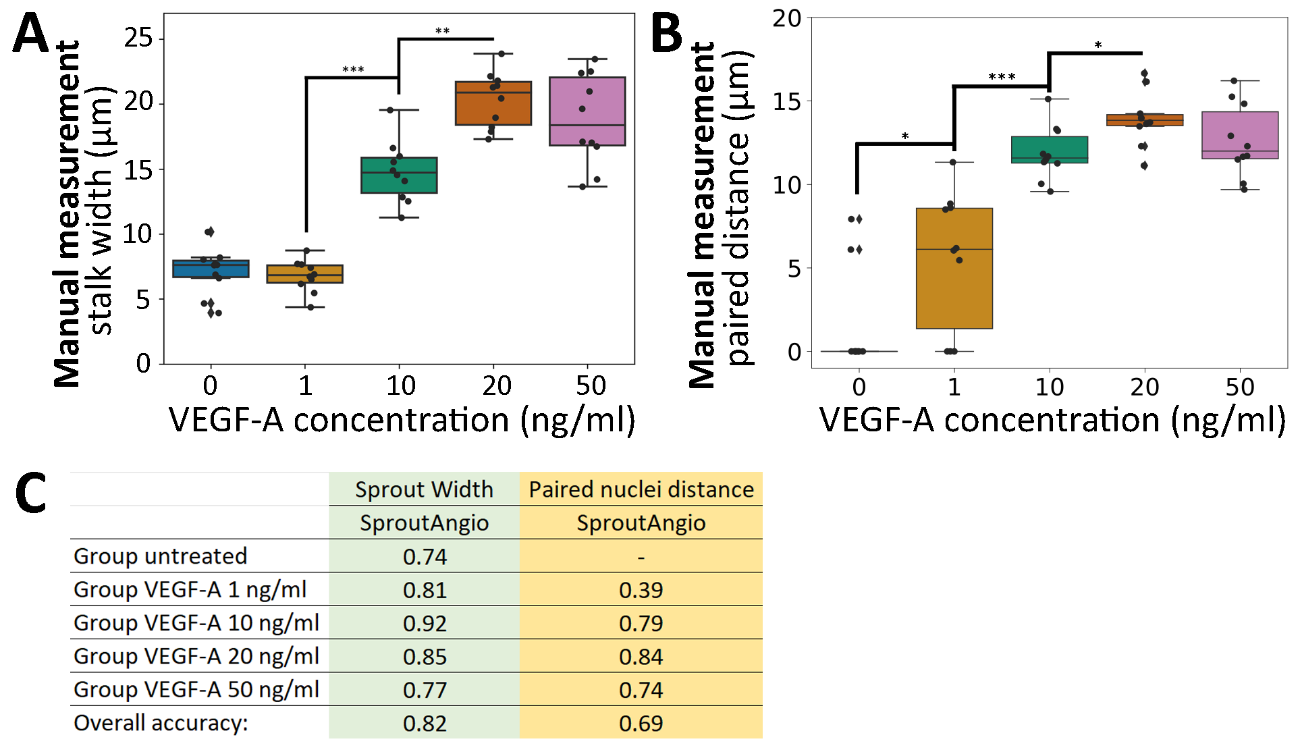


**Supplementary Figure 4:** **A)** Manual measurement of the sprout width at the center of the sprouts (stalk width) for the VEGF-A treatment groups. **B)** Manual measurement of the paired nuclei distance from the raw images using Napari viewer and Pythagorean theorem for distance. **C)** The accuracy scores of automated lumen analysis results for each VEGF-A treatment group (0-50 ng/ml) in fibrin bead assay. Accuracy scores were calculated using manual measurements. Scale 0-1, 1 being the ground truth. Kruskal-Wallis test was used to determine the statistical significance. * p<0.05, ** p<0.01, *** p<0.001.


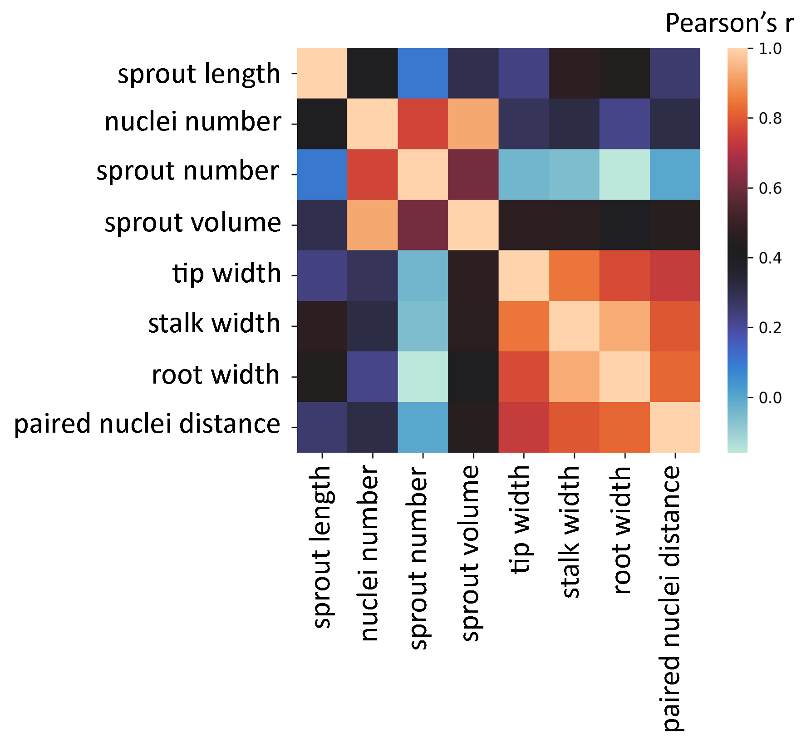


**Supplementary Figure 5**: Heatmap of the correlation analyses obtained from the fibrin bead assay data. Scale 0-1. The red color tones indicate a stronger correlation. Pearson’s correlation was used for the analyses. r 0.20-0.39 weak correlation, 0.40-0.59 moderate correlation, 0.60-0.79 strong correlation, 0.80-1.00 very strong correlation.


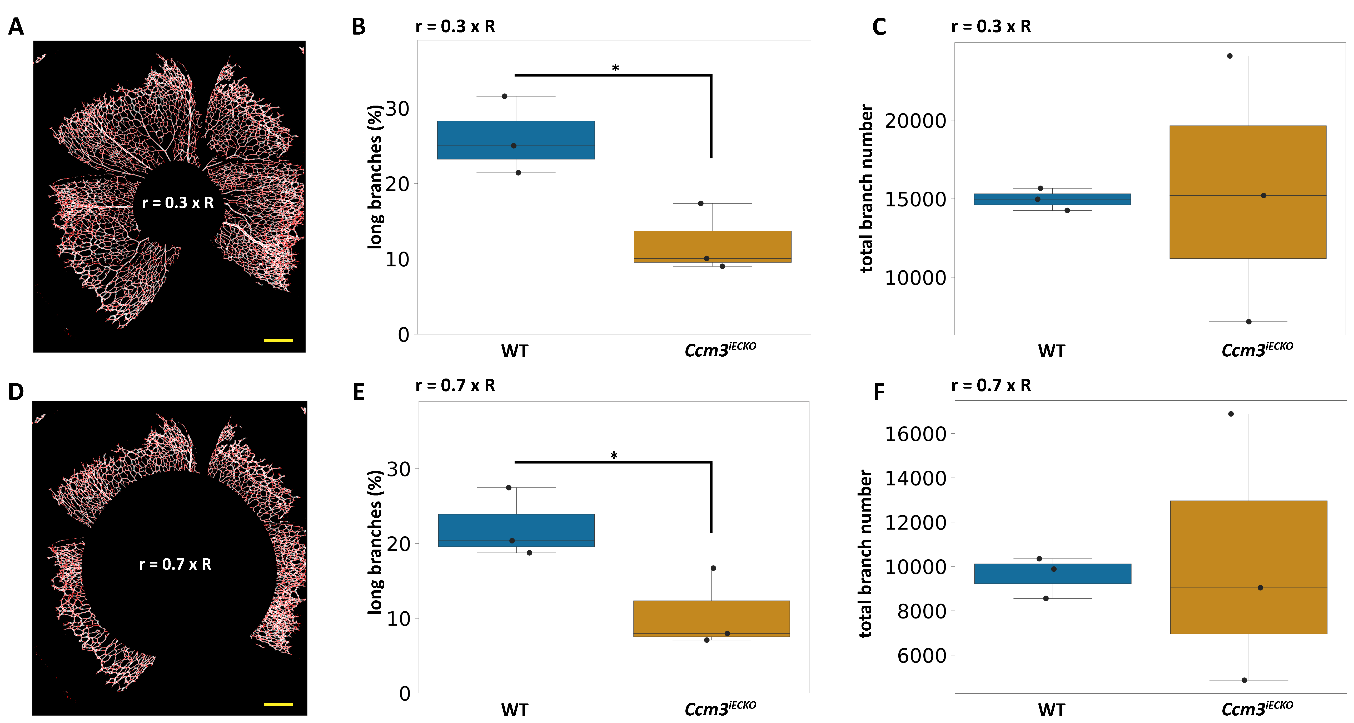


**Supplementary Figure 6**: Retinal images showing the outside circle closer to the migration front were used for the analysis. Vasculature was labeled with anti-CD93. **(A)** r = 0.3 x R was used to exclude the branches in the central part of the retina. **(B)** Percentages of long branches for the WT and *Ccm3^iECKO^* dataset for r = 0.3 x R showing the significant difference between the groups, with p<0.05. **(C)** There was no significant difference in the total number of branches between the WT and *Ccm3^iECKO^* groups, for r = 0.3 x R. **(D)** r = 0.7 x R was used to exclude the branches in the central part of the retina. (**E, F**) Percentages of long branches for r = 0.7 x R showing a significant difference, whereas no significant difference for the total number of branches, for r = 0.7 x R. In all images, t-test was used to determine the statistical significance *, p<0.05. Scale bars: 500 µm.


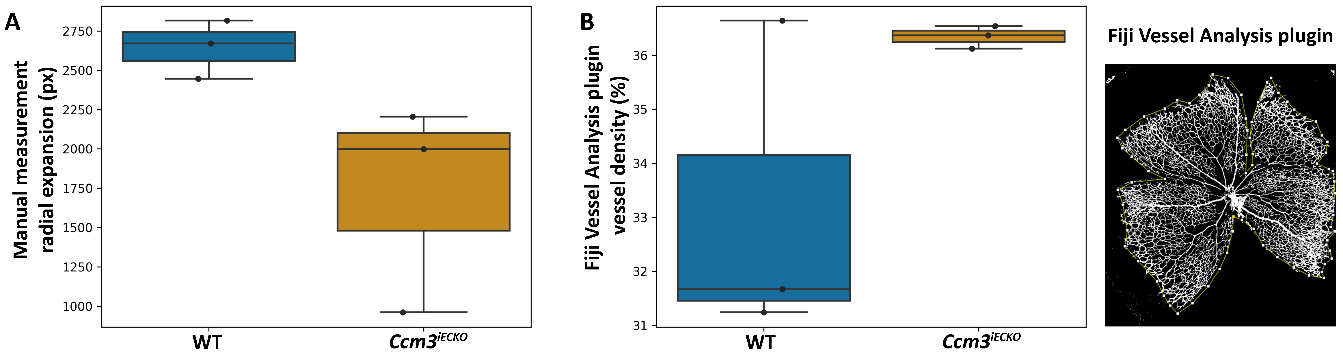


**Supplementary Figure 7**: **A)** Manual radial expansion measurement showed no significant difference between the groups confirming the SproutAngio results. **B)** ImageJ Fiji Vessel Density plugin was used for comparison purposes. There was no significant difference between *Ccm3^iECKO^* and wild-type retina samples. Biggest difference between Fiji plugin and SproutAngio analysis was the region selection. In Fiji plugin the whole area was manually selected for the analysis. In SproutAngio, the central region was removed by using a user-defined threshold and was not included in the vessel analysis. n=3 mice/group. t-test was used to determine the statistical significance.
