# Supplementary material for: SproutAngio: an open-source bioimage informatics tool for quantitative analysis of sprouting angiogenesis and lumen space
Source: Sci Rep. 2023 May 4;13:7279. doi: 10.1038/s41598-023-33090-6 (PMC10160097; doi:10.1038/s41598-023-33090-6)
Supplement: Supplementary file 2 — Supplementary Information 2. [file 41598_2023_33090_MOESM2_ESM.docx]

**Step-by-step User Manual for SproutAngio**

**A. INSTALLING**

**1**. If you are new to Python, one of the best ways to install and use SproutAngio tool is by installing Anaconda Navigator. It simplifies the package management and installation process. Install Anaconda using their updated installation guide here: <https://docs.anaconda.com/anaconda/install/>

Note! After the installation of Anaconda update it to the current version if needed.

**2**. Before installing SproutAngio and the necessary libraries, you need to create a new working environment using Anaconda. It always opens the base (root) environment as a default (marked by yellow rectangular in the figure below).


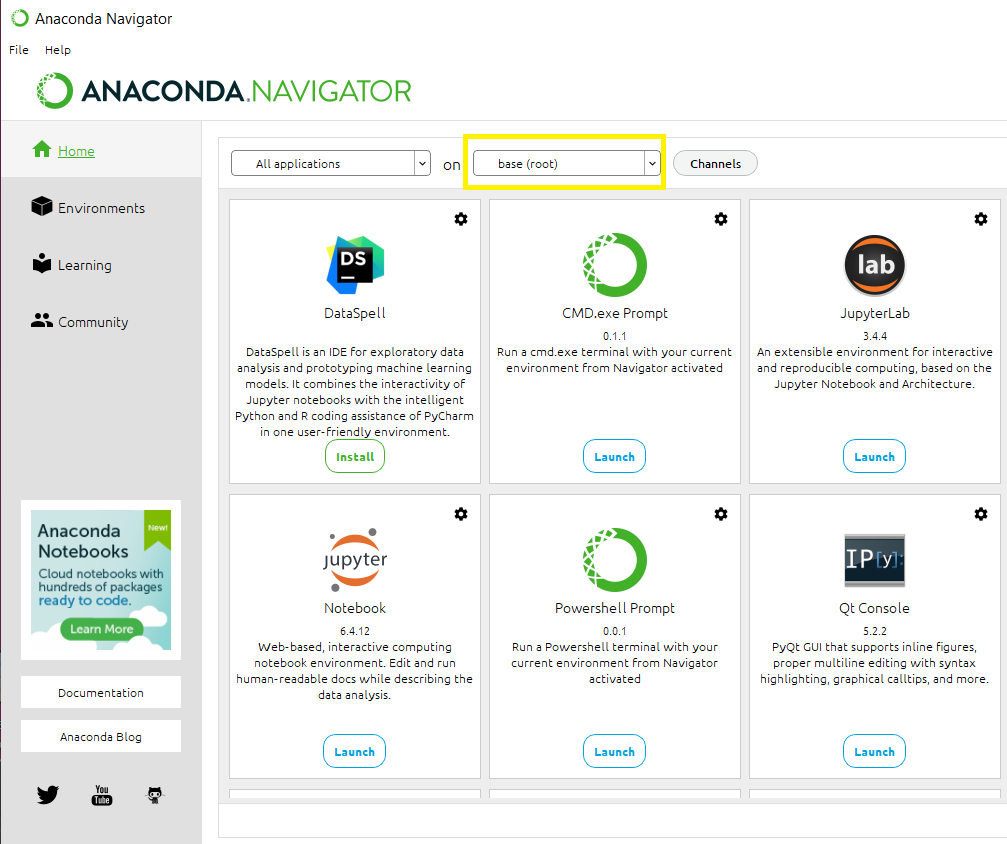


**3**. Click “Environments” and then “Create” (marked in yellow in the figure below) to create a new working environment for SproutAngio.


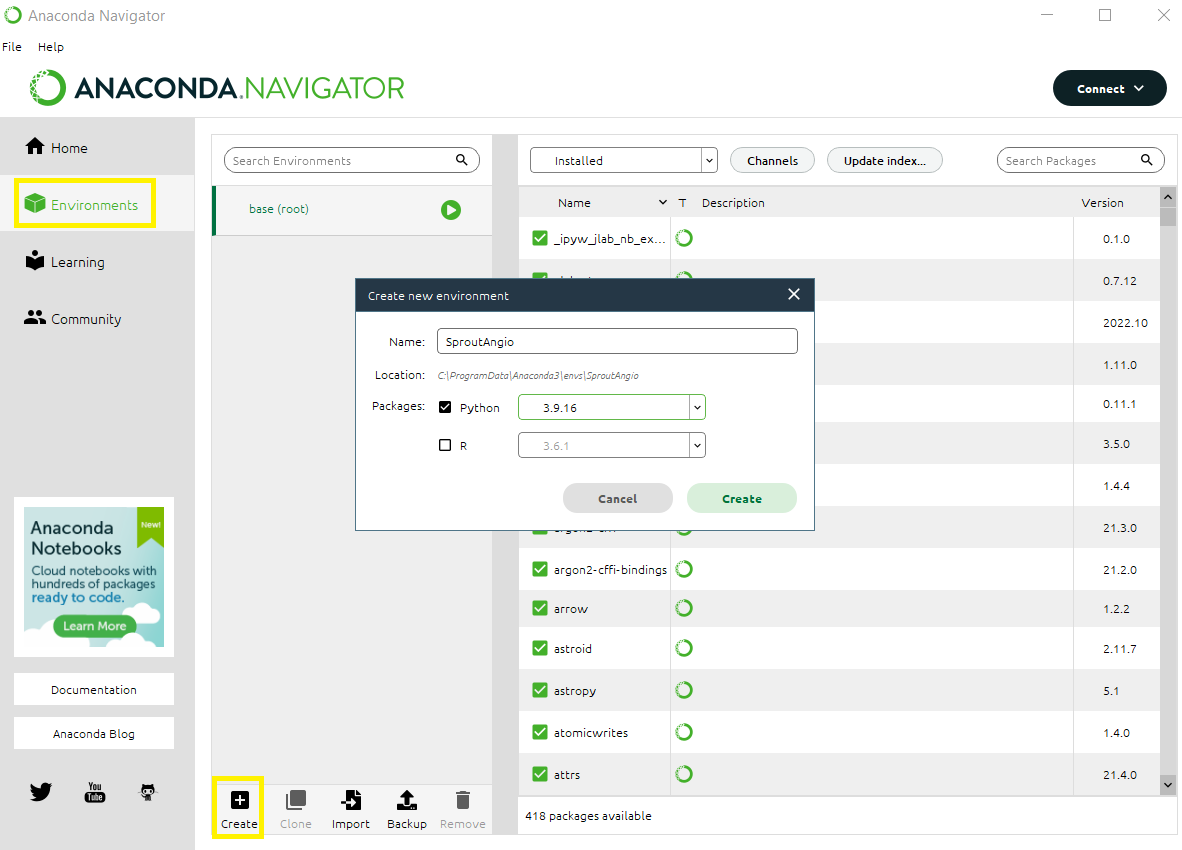


**4**. After creating the working environment, you can now go back to the home tab of Anaconda Navigator. Then, you can install one of the interactive environment plugins, in this case we prefer using JupyterLab (marked with yellow rectangular to figure below) simply because we feel it is easier to use.


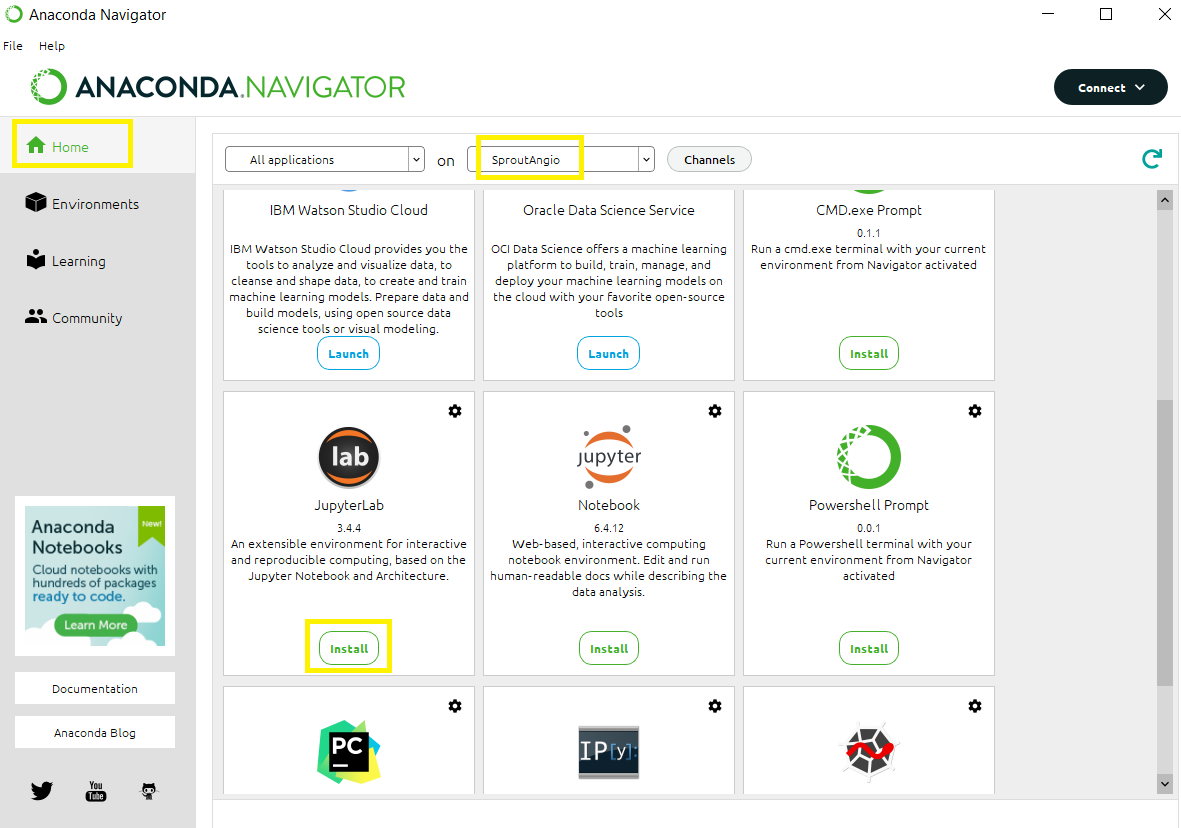


**5**. Once JupyterLab is installed, you can launch it (marked in figure with yellow).


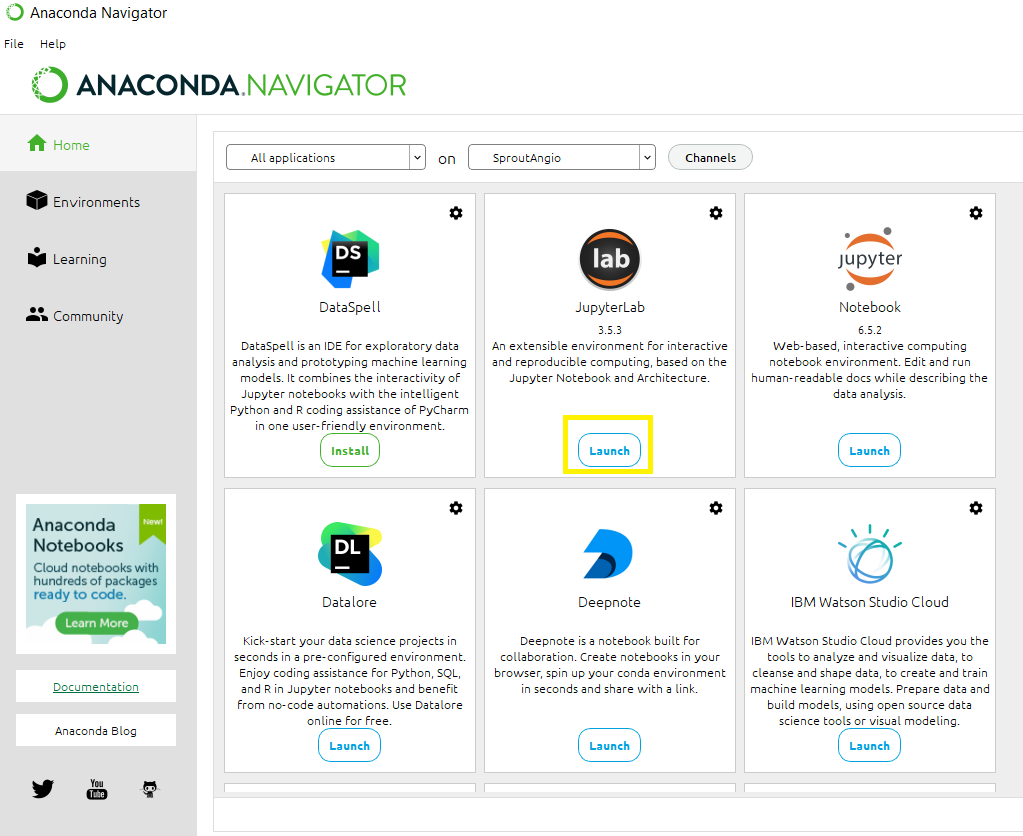


**6**. JupyterLab will simply open using your internet browser. Notice that Jupyterlab has multiple tabs in both left and right sides. Left tabs are file browser, terminals and kernels, table of contents, extension manager. On the right tab, you can create new launcher tabs by clicking the + icon.


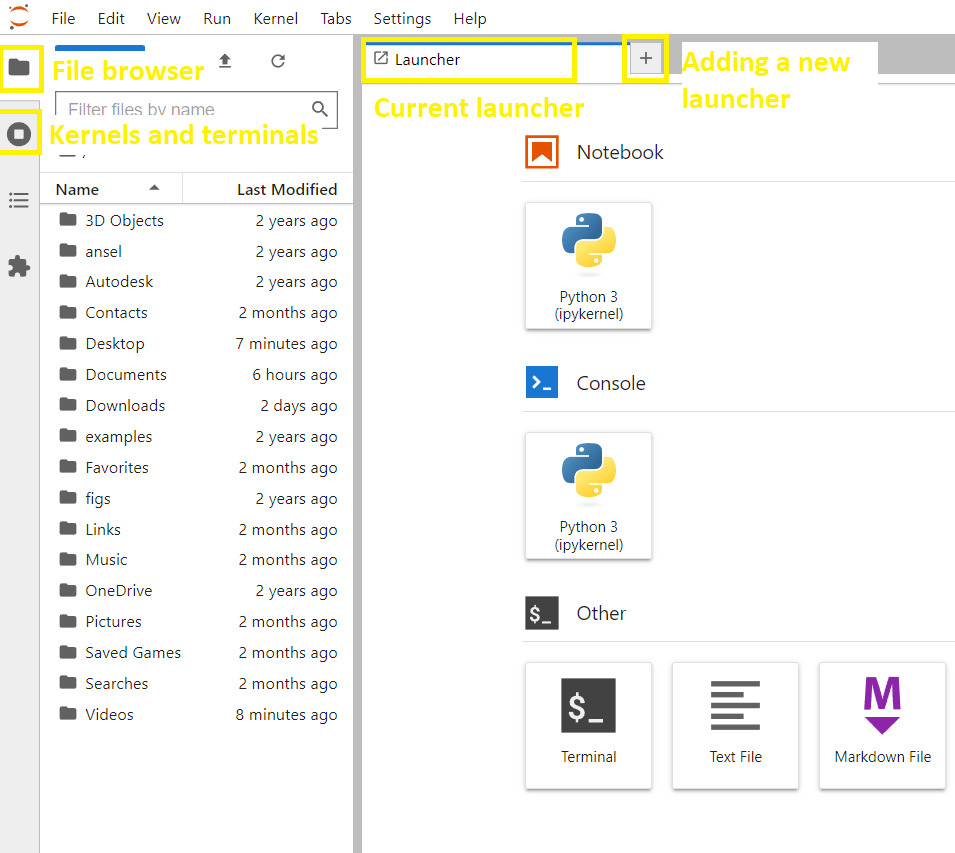


**7**. Now select a file path for your working environment. As an example, here we double-click the Desktop in the browser (marked in yellow in the figure below). If you do not see your desktop, you can alternatively choose some other file path you know such as Documents or Downloads.


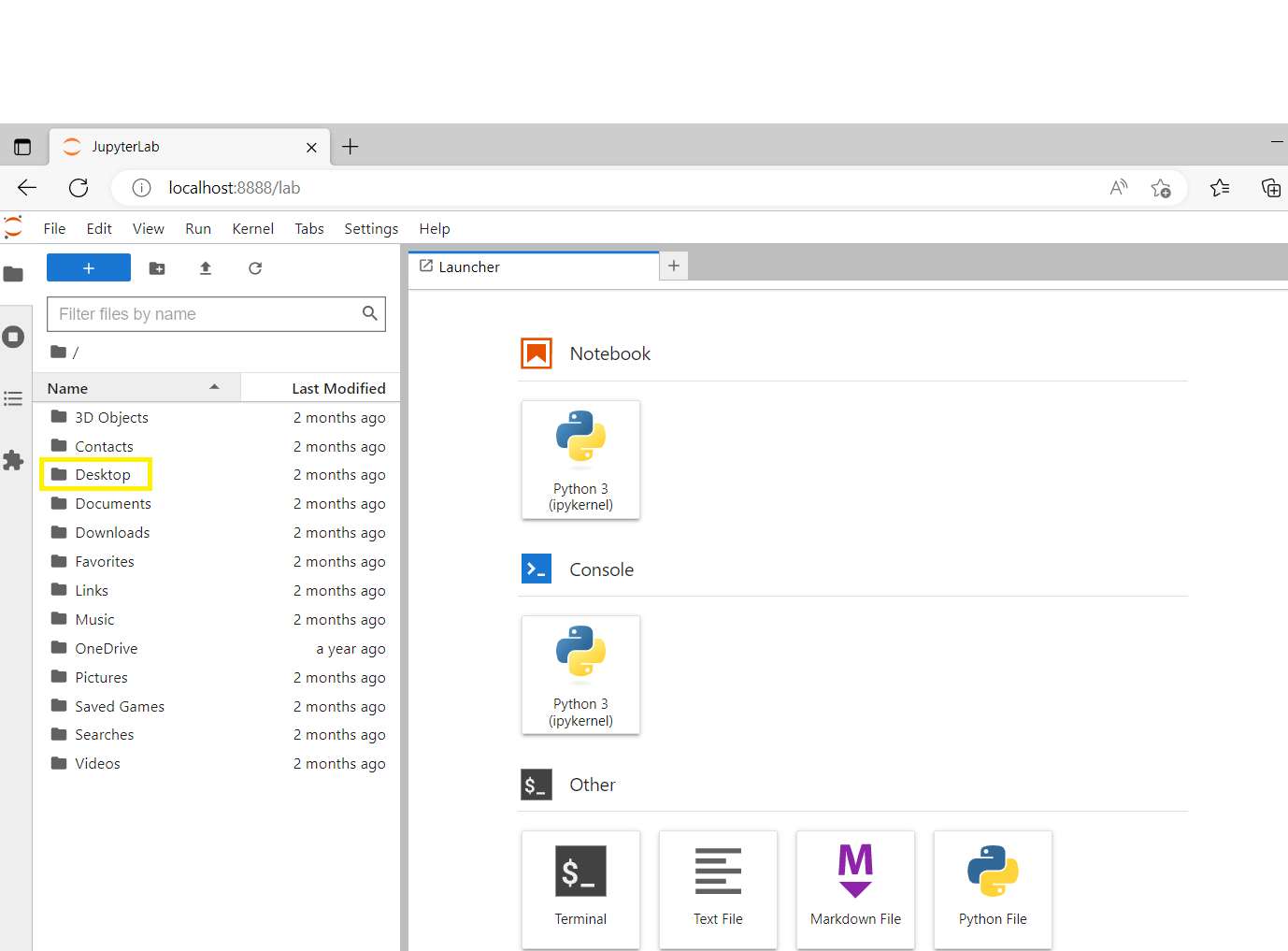


**8**. Then, click the new folder icon to make a new folder on your desktop (see arrow in the figure below). We name it here as SproutAngio.


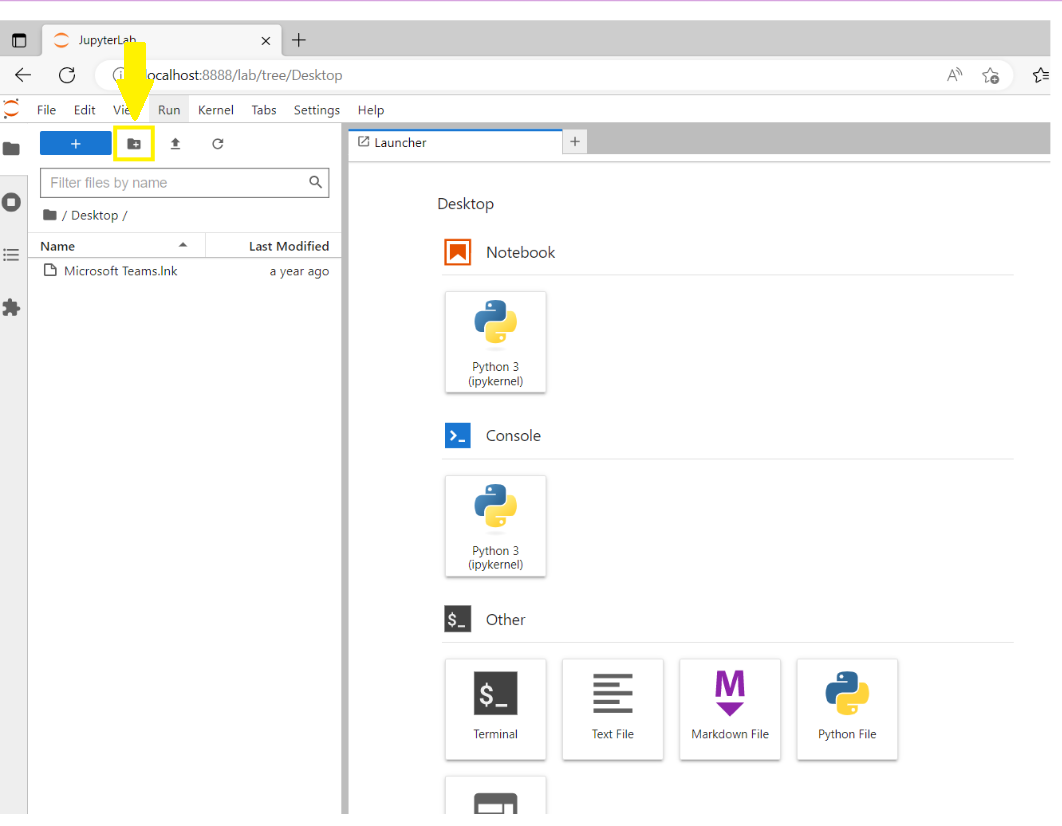


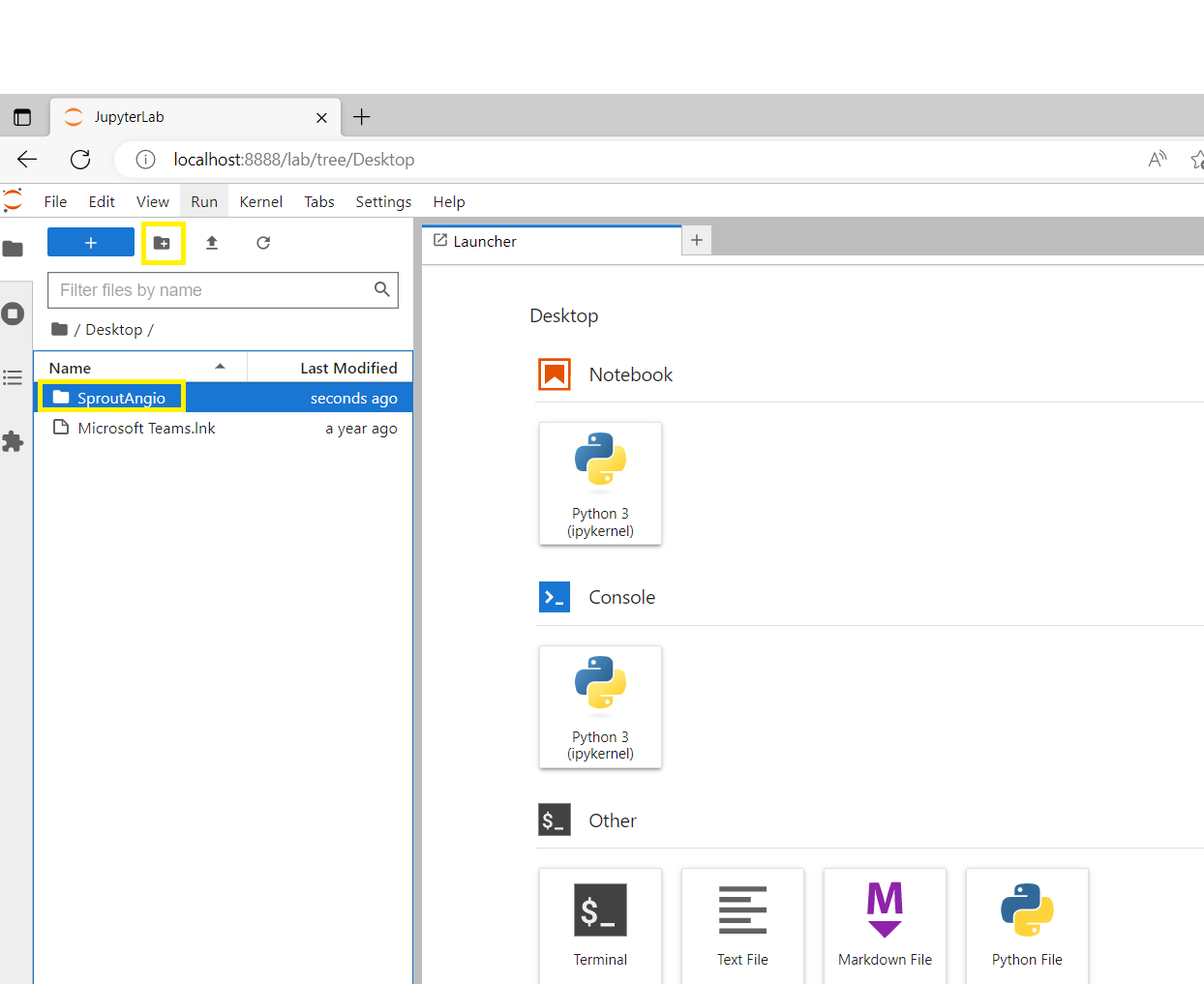


**9**. You can double click and get inside the newly created SproutAngio folder, which is empty right now.


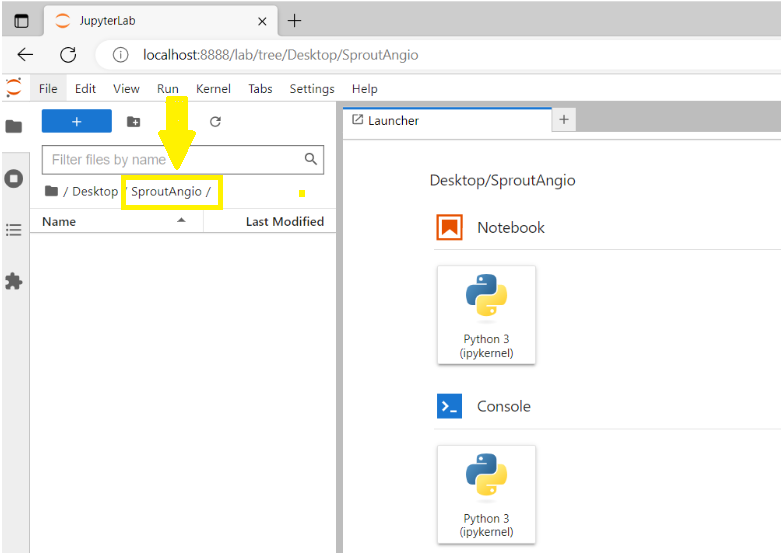


**10**. Now, you will install the necessary dependencies to use the SproutAngio tool. By clicking on the Terminal, we will open a terminal (marked in yellow in the figure below).
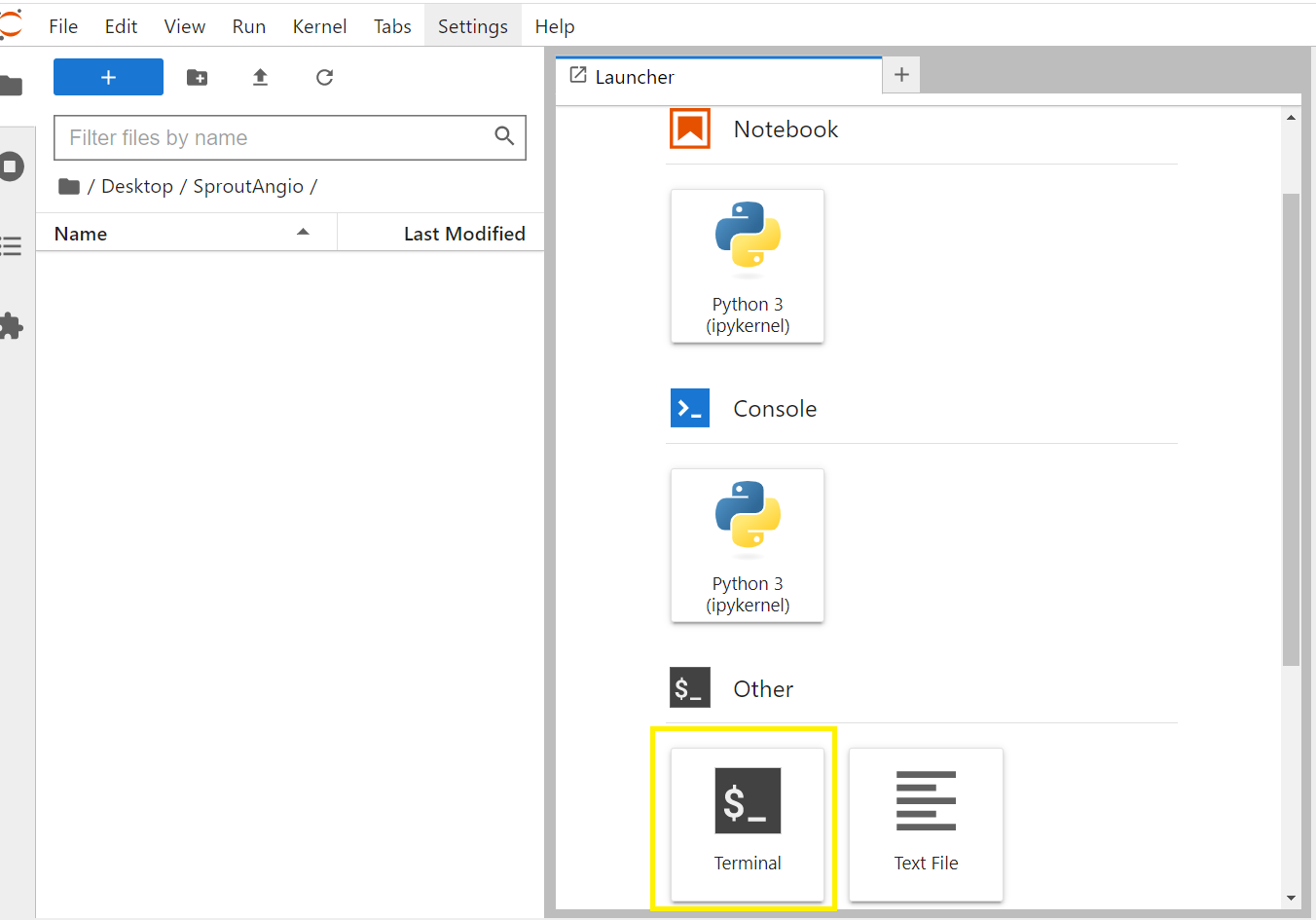


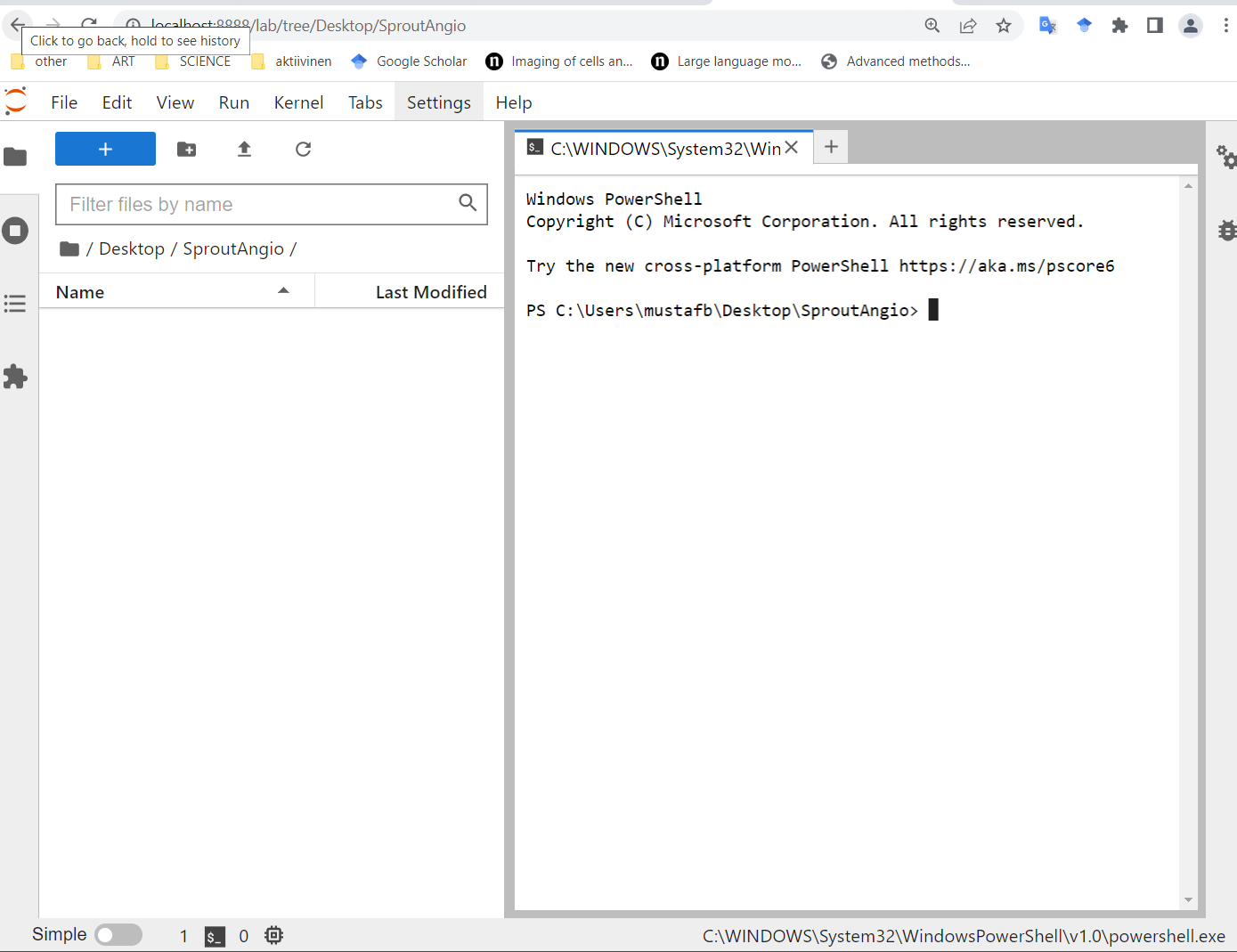


**11**. Next part is the installation of the prerequisites to use SproutAngio. Before reading the next parts, just a reminder that installations might take couple of minutes especially for the first library (Napari) and when you run, it might seem that it is doing nothing in the beginning, but actually it is. So, be patient.

Before you continue, to briefly explain the function of each library:

Napari is a multi-dimensional image viewer for Python environment. Czifile is a library to read confocal microscopy Carl Zeiss Image (CZI) files. Seaborn is a data visualization library. Fil-finder is a package to analyze filamentary structures in fibrin bead assay. Skan is a library to analyze skeleton structures in retina images. Openpyxl and XlsxWriter are libraries to handle and transfer data in excel files.

**12**. Now, copy (ctrl – C) paste (ctrl - V) the installation commends, one by one, for the prerequisites and press “enter” key on your keyboard. You can check when the installation of each package finishes, by seeing the same starting path for your new commend:

pip install "napari[all]"

**13**. Then press enter on your keyboard. Note that installation might take couple of minutes depending on the internet speed and processing time.


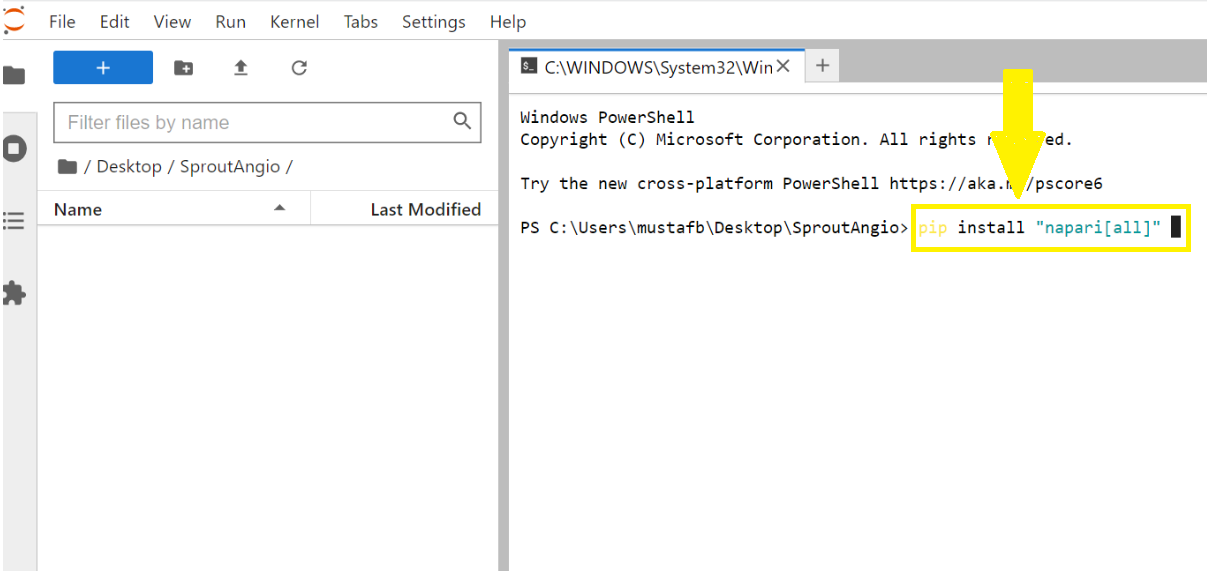


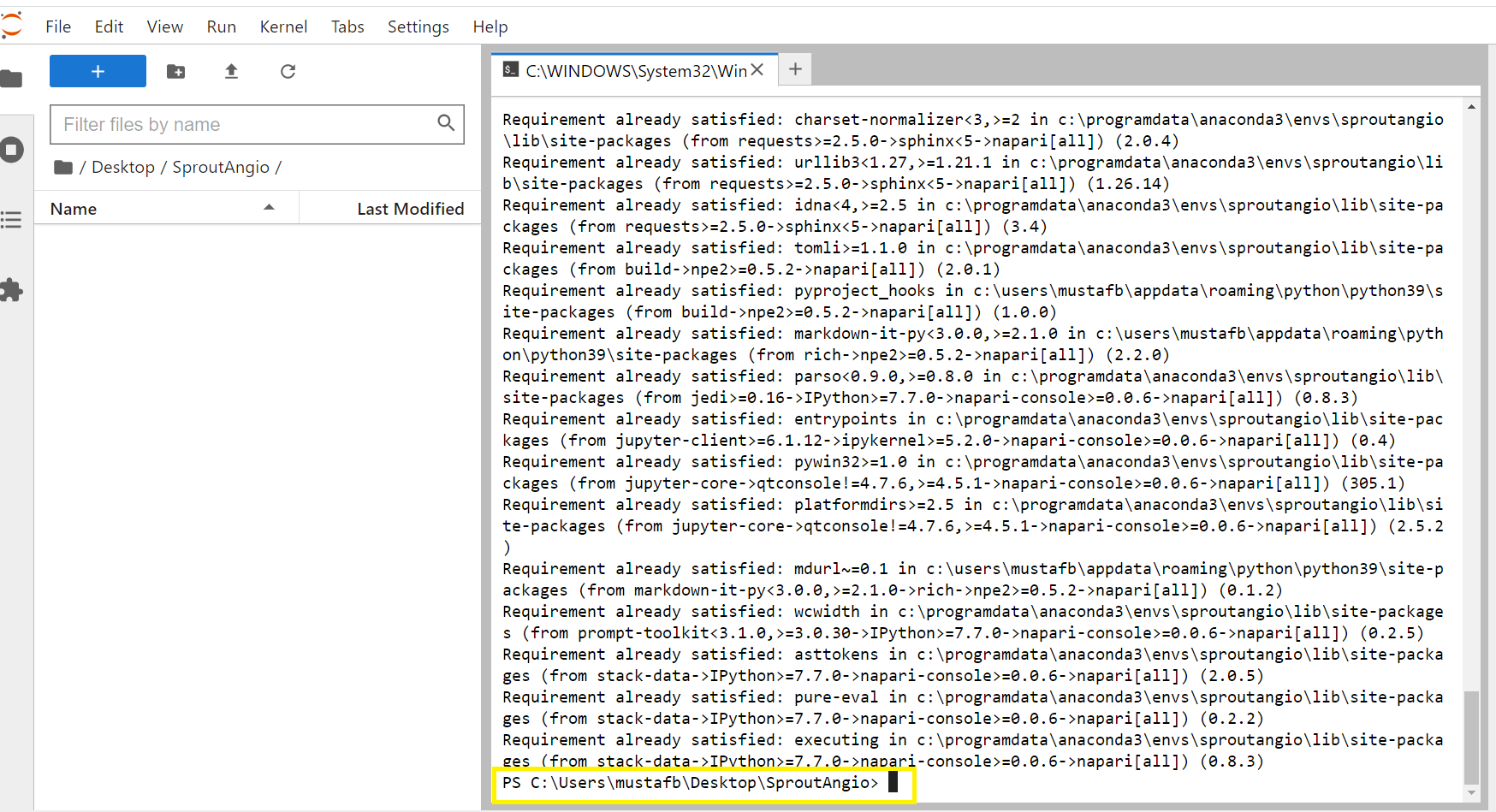


**14**. Once you see the Users\your username... line, it means the installation is finished for that library. You can do the same copy-paste with these next libraries:

pip install czifile

Then press enter

pip install seaborn

Then press enter

pip install fil-finder

Then press enter

pip install skan==0.10.0

Then press enter

pip install openpyxl

Then press enter

pip install XlsxWriter

Then press enter

**15**. After all the packages are installed, you can close the terminal:


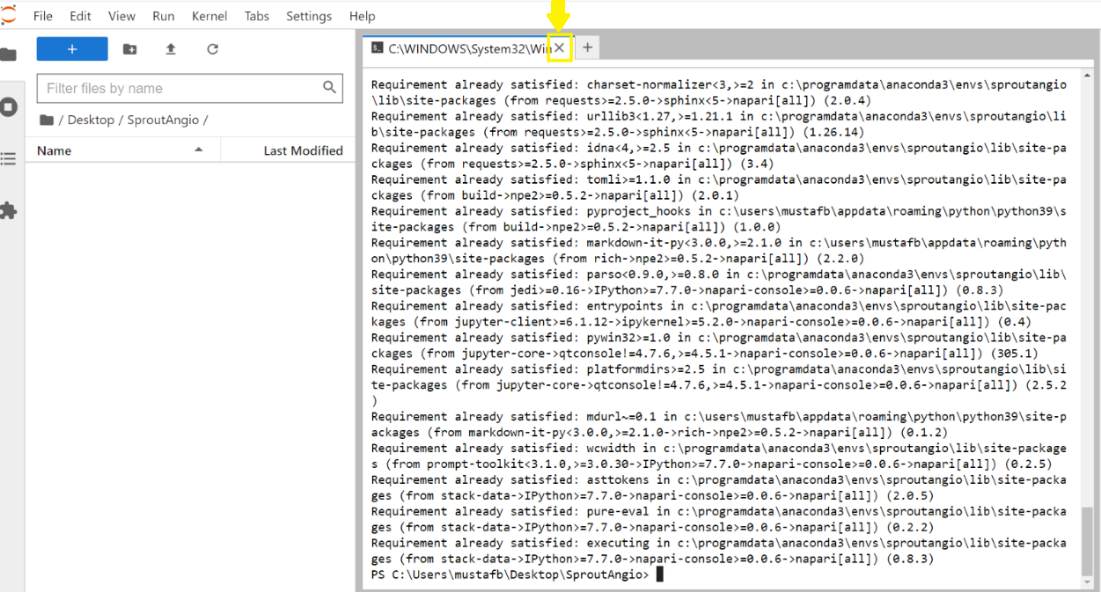


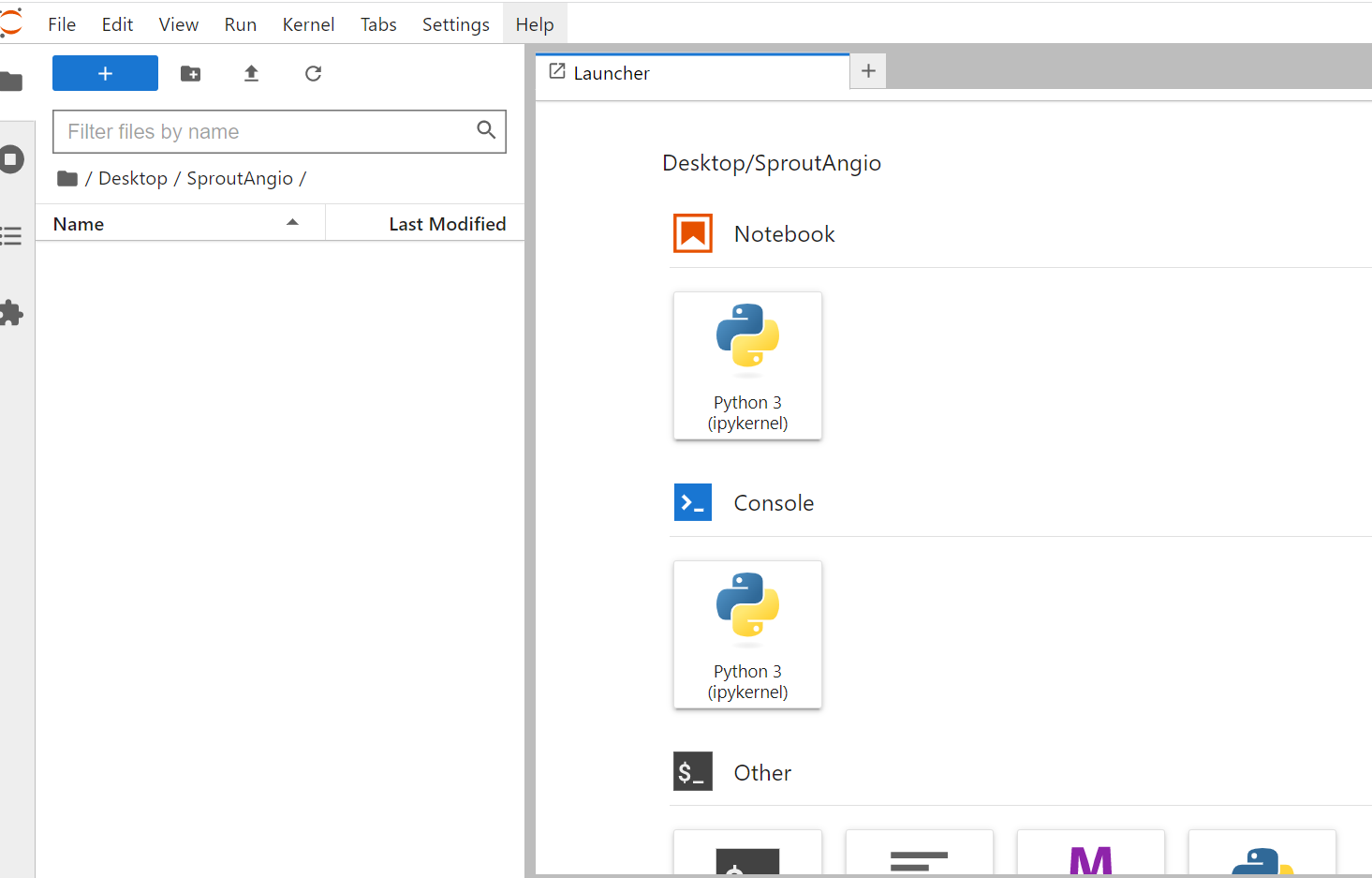


**16**. Now you can download SproutAngio files from GitHub, i.e., a website and cloud-based service used for storage and management of codes. If you do not have experience with using GitHub, we recommend using GitHub desktop application. To install it: <https://desktop.github.com/>

**17**. After installing GitHub, you can clone SproutAngio repository following the next steps (steps 1,2,4,5).

Manual Uniform Resource Locator (URL) for SproutAngio, for step 2 cloning steps below is: <https://github.com/mbbio/SproutAngio>


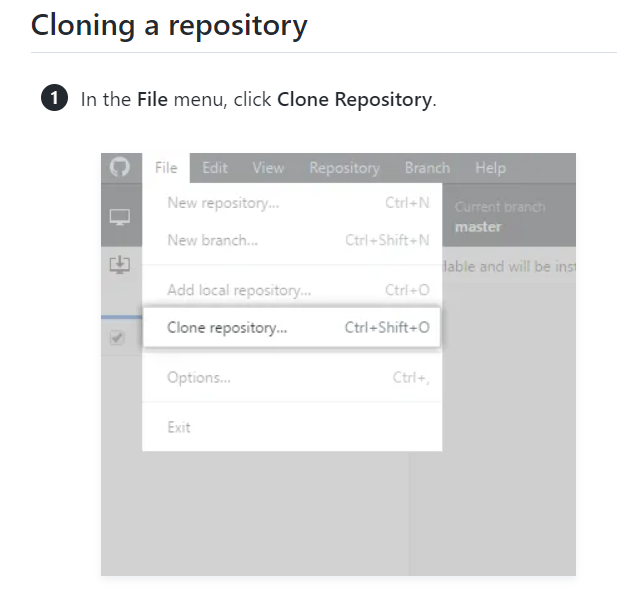


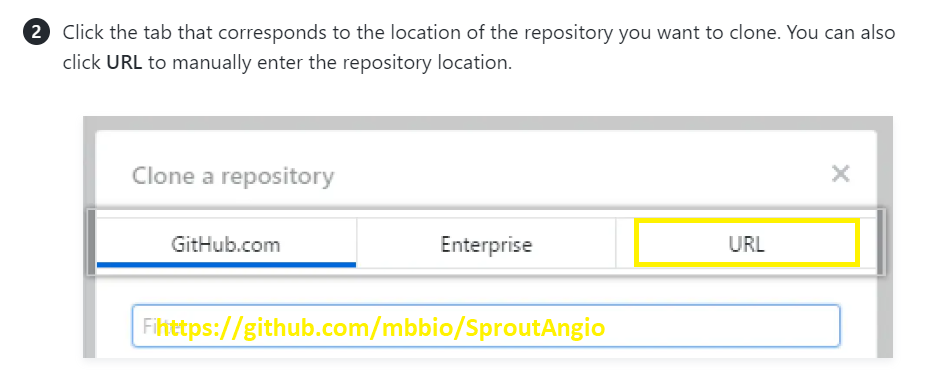


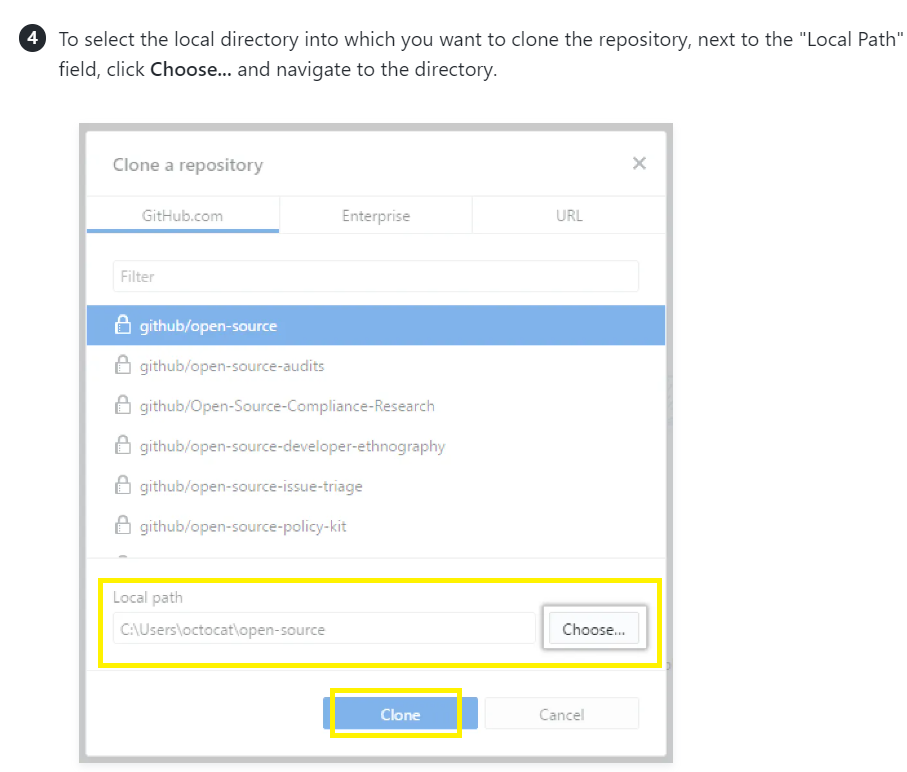


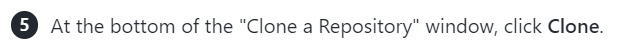


Note: Alternatively, you can download the SproutAngio-main files here:

<https://github.com/mbbio/SproutAngio/archive/refs/heads/main.zip>

However, if you download the files from the link directly, you need to unzip the “SproutAngio-main" zipped folder. Then you need to copy paste the files into the SproutAngio folder you created previously in JupyterLab.

**18**. After you downloaded the SproutAngio-main files, you still need to re-download the test samples. Normally, test data are uploaded in GitHub but, currently GitHub protocol does not let the downloading of large sized files unless it is from a direct link or Git Large File Storage (Git LFS). So, if you are not familiar with using Git LFS, you can click download the data here:

<https://github.com/mbbio/SproutAngio/raw/main/test_data/VEGFA_1ng.czi>

<https://github.com/mbbio/SproutAngio/raw/main/test_data/VEGFA_10ng.czi>

<https://github.com/mbbio/SproutAngio/raw/main/test_data/VEGFA_20ng.czi>

**19**. After your download is complete, copy paste these test samples inside the test_data folder inside your SproutAngio folder. (Notice that SproutAngio folder is the one you created using Jupyterlab, while SproutAngio-main folder is the one you downloaded from GitHub.)

Note! When you open the test_data folder for the copy-paste, you can see that there are already files with the same name in test_data folder but their size is 1kb, so they are not the actual image files. You can delete them.

**20**. Turn off the GitHub application and be sure to copy paste all 6 files below from GitHub downloaded files and test samples into the “SproutAngio” folder which you created with Jupyterlab. After all these files are in your Jupyterlab created folder, when you open the Jupyterlab tab in your internet browser, you can see the files in your folder there.


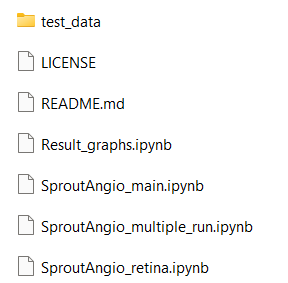


**21**. Well done! Now, everything is ready to start using the SproutAngio tool.

**B. USING THE TOOL:**

**1**. To start using the SproutAngio, double click the SproutAngio_main file on the left (yellow rectangular in the figure below).


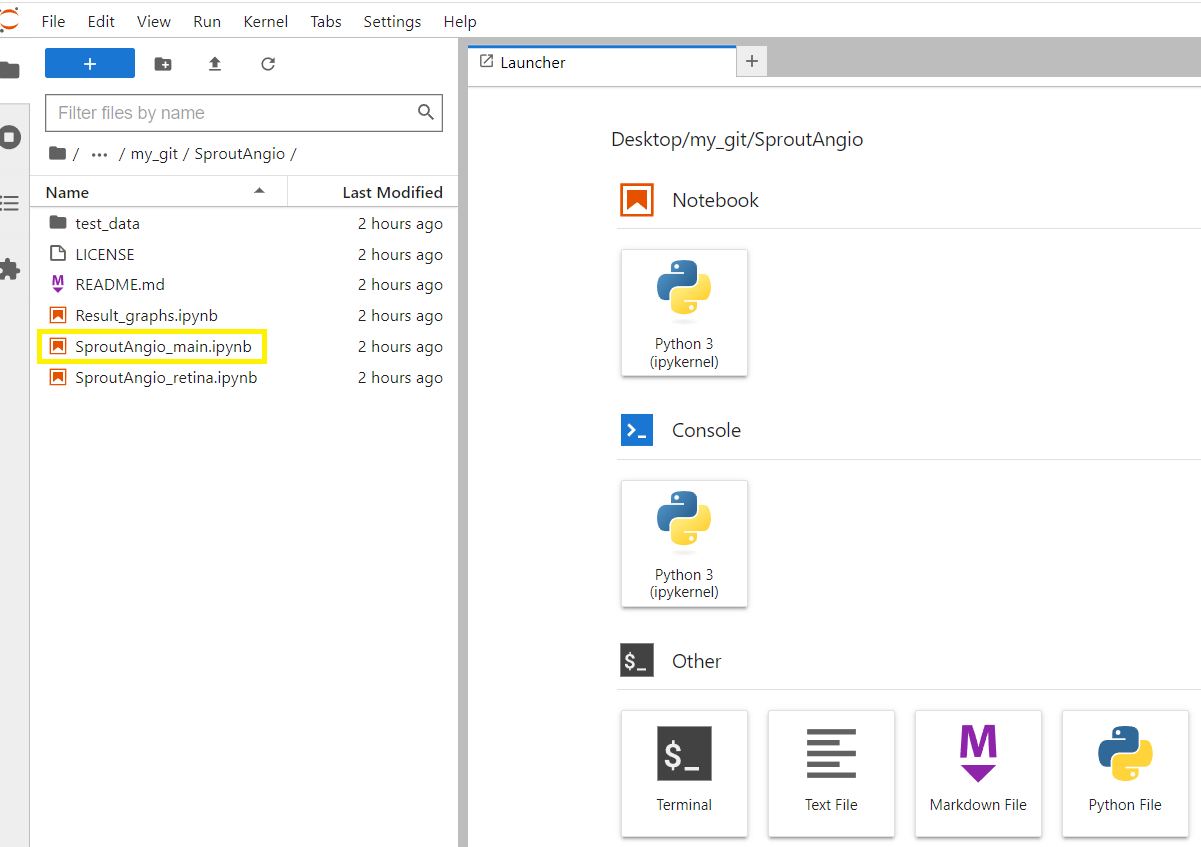


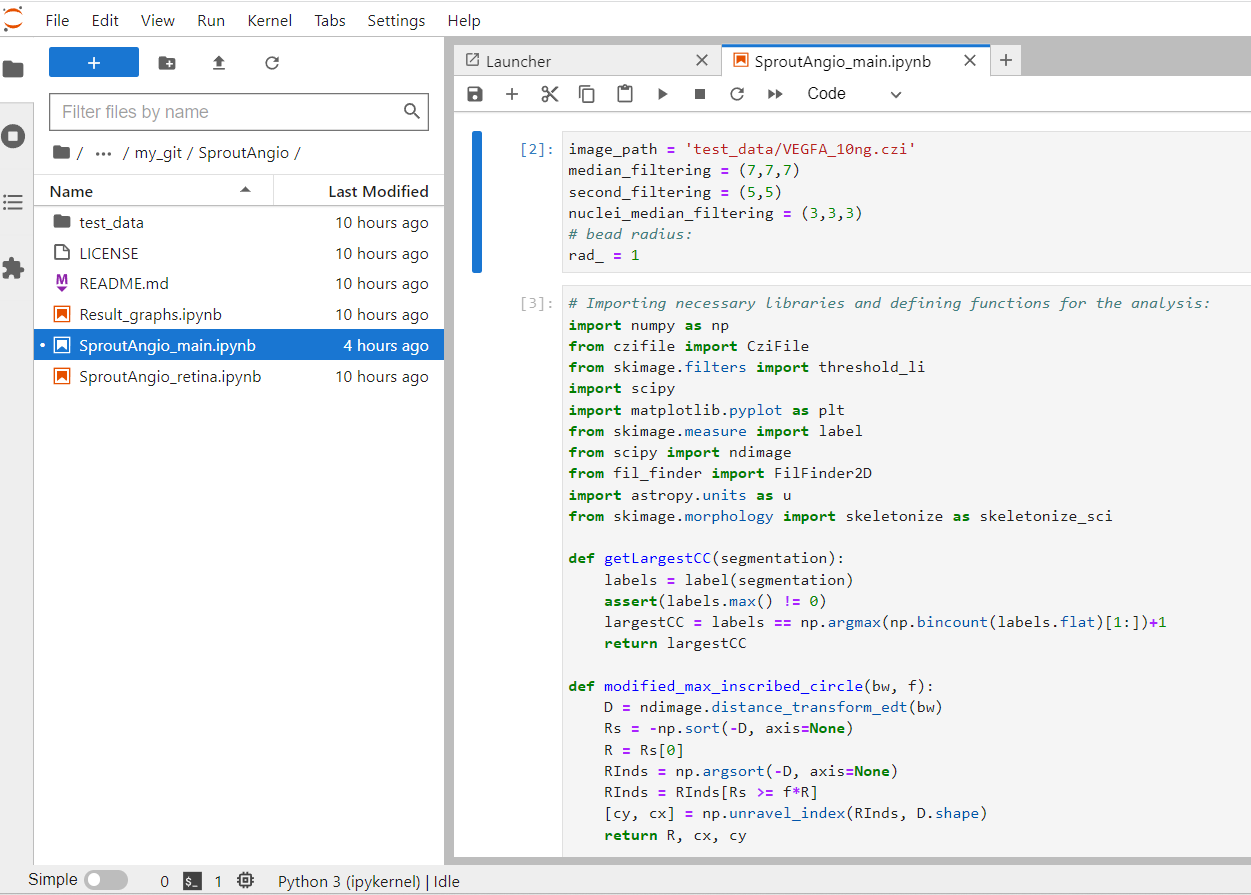


**2**. In this test run, we are using one of the images from our VEGFA dataset. So, if you cloned the github repository using github application and downloaded the test_data files seperately as instructed so far, then you do not need to change the path: 'test_data/VEGFA_10ng.czi'

However, if you downloaded the image samples from zenodo or if you are testing any other image file, then change the path. For example, if you have an image file named “FILENAME.czi”, inside test_data folder, then you need to change the image_path into:

'test_data/FILENAME.czi'


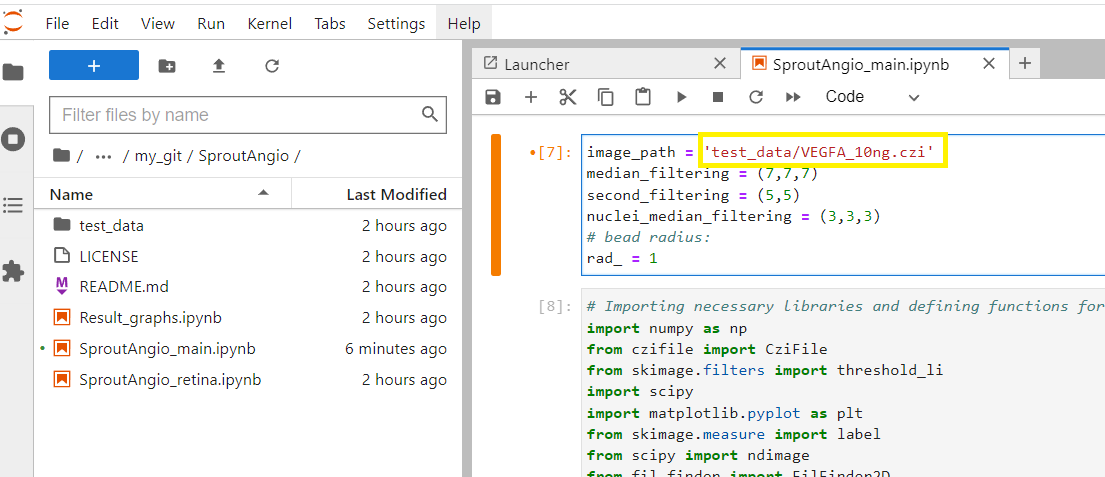


**3**. If you are not familiar with using Python, the code on the right is separated into different sections. Every section has a name referring to its function. When you click inside each section, you can run the code for only that section by clicking the “run” button. You need to run the first section first. This section includes the user defined basic parameters.


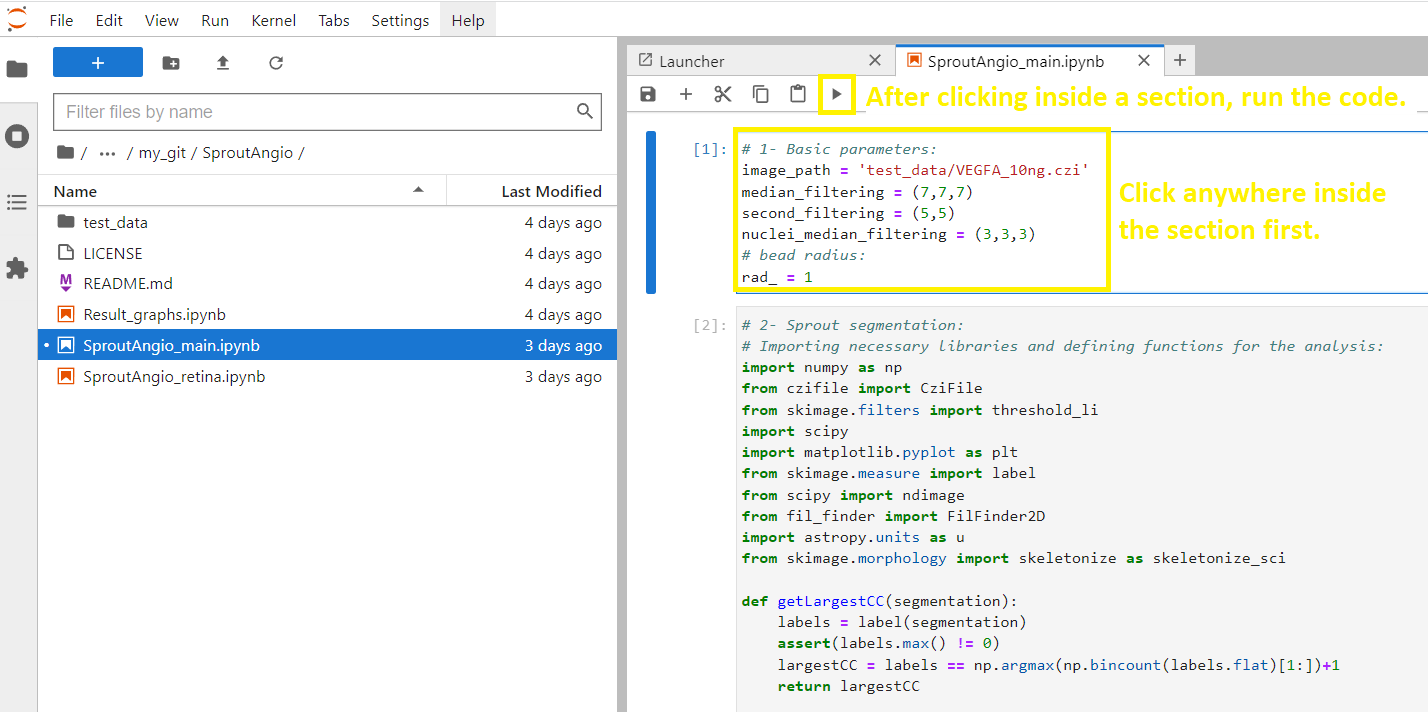


**4**. Then, you can click inside anywhere the second section and run it (see below the yellow rectangular). This section includes: importing the necessary libraries, channel separation, filtering, thresholding, skeletonization, bead removal and sprout segmentation.
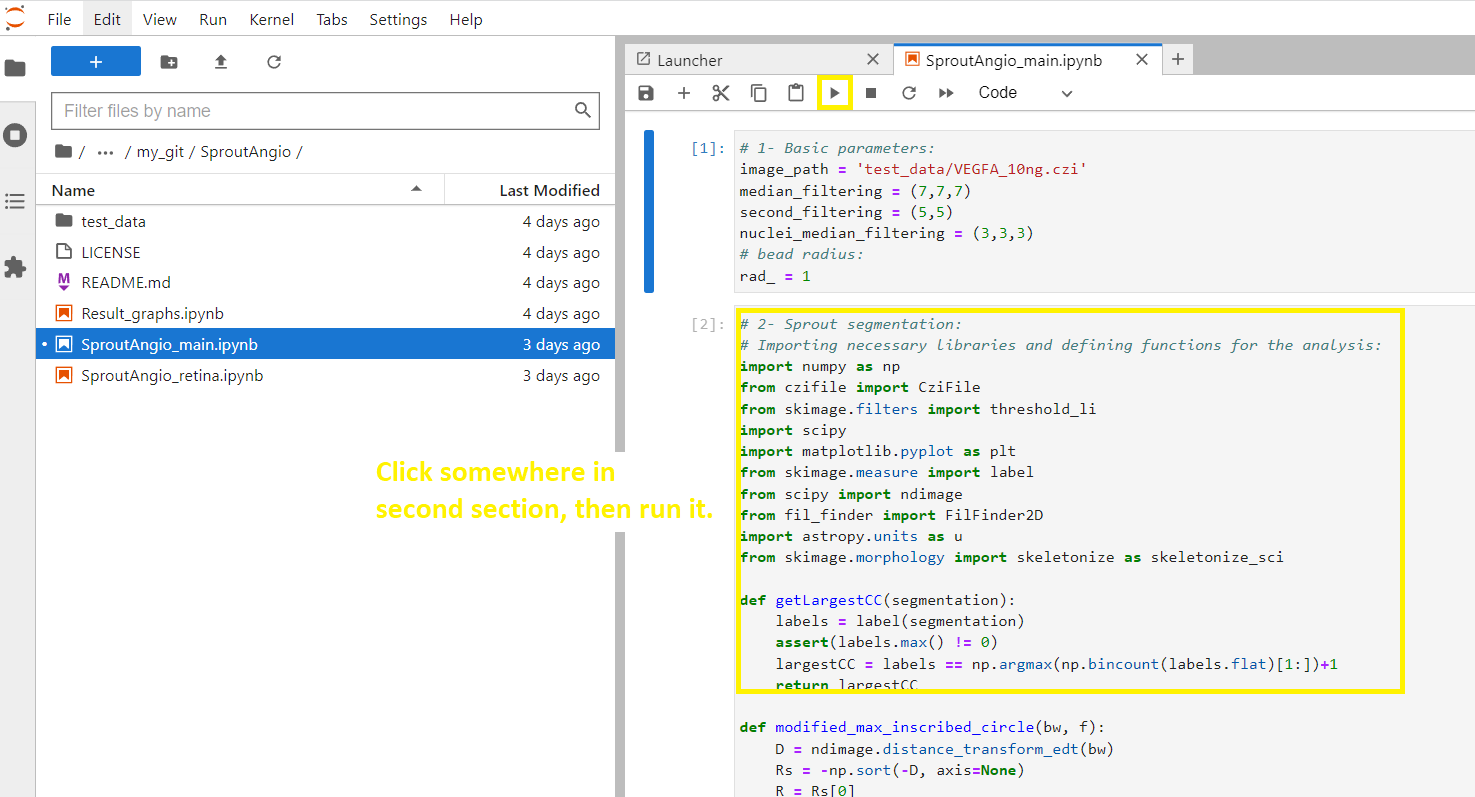


Note! Running of this section will take some time depending on your computer’s power and you can observe this from the bottom of the panel which says “Busy” during the run. When the run finishes, it writes “Idle”.


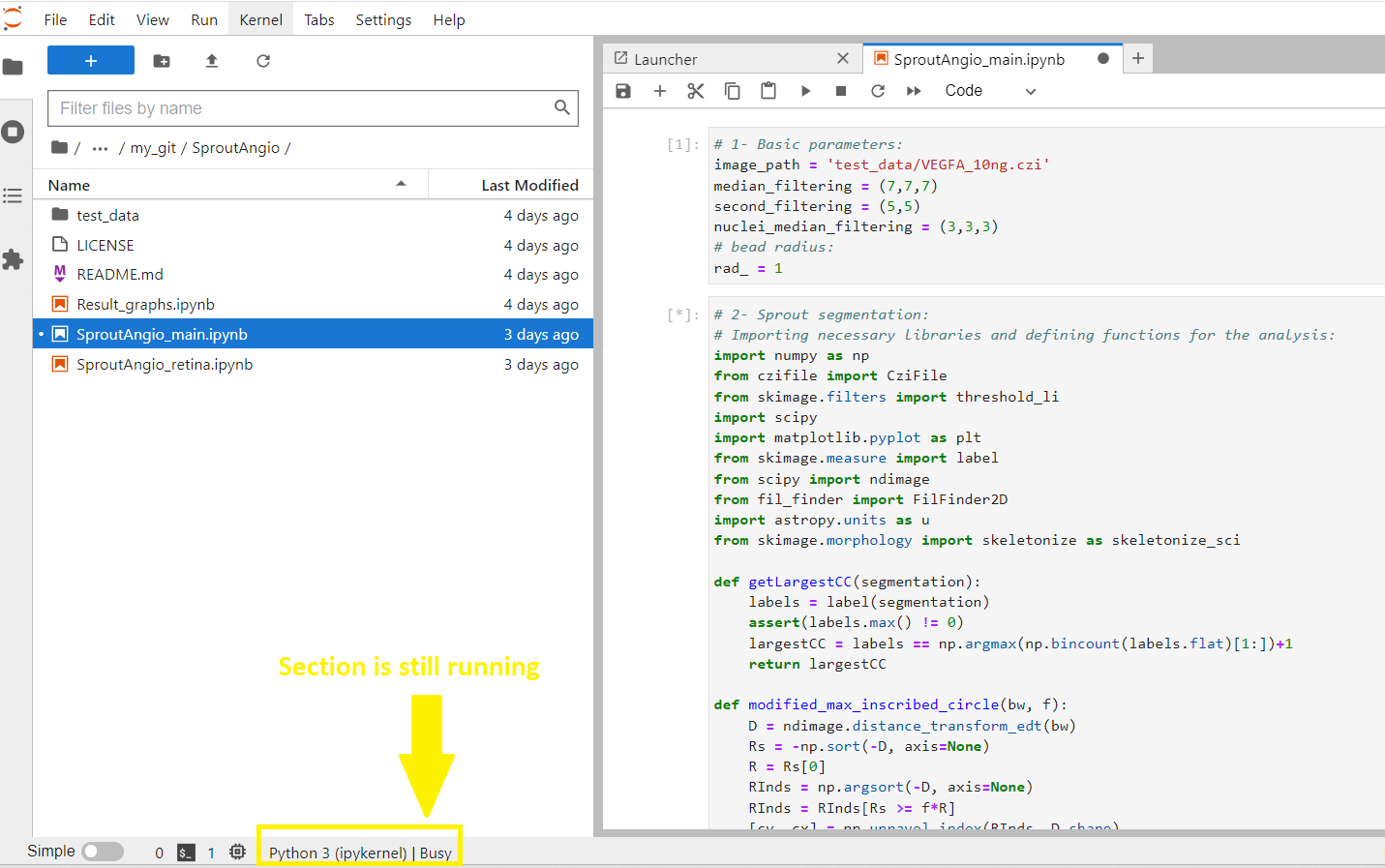


**5**. Once it finishes, a Napari user interface window opens.


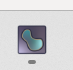


**6**. Now you have two application screens:

i) a SproutAngio Jupyterlab tab which is working on your browser

ii) a Napari viewer screen (not on your browser but as independent screen). Click on the Napari symbol to switch to Napari viewer screen.


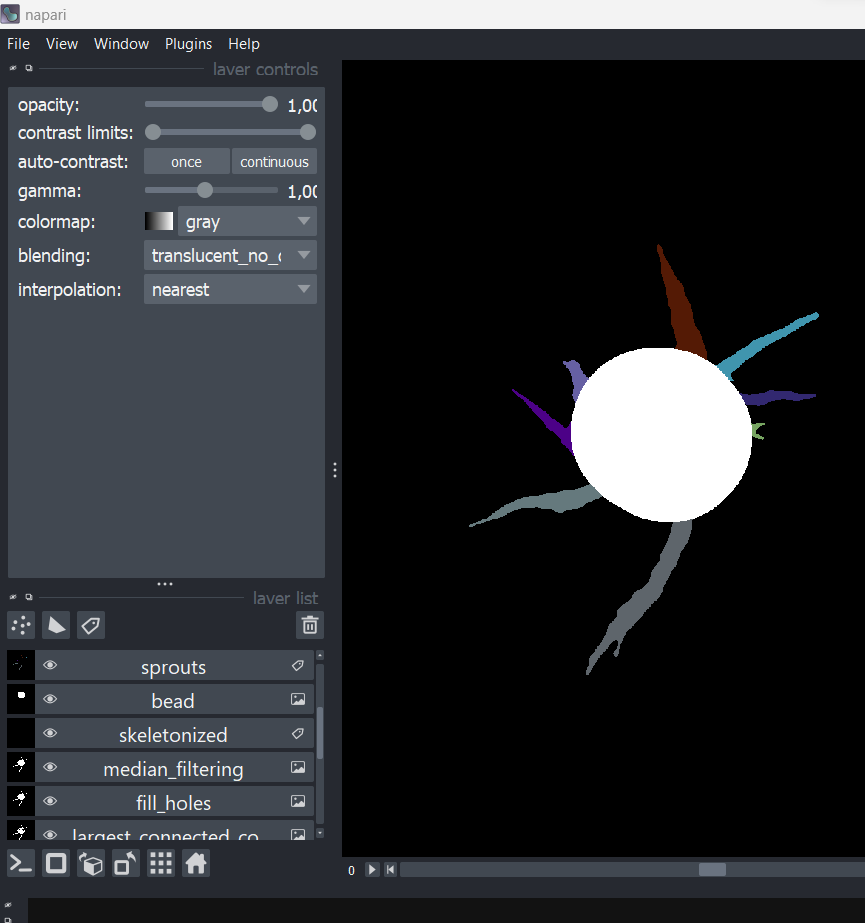


**7**. Now continue in SproutAngio.

The next section is nuclei segmentation part (marked with yellow rectangular in the image below). It includes importing the libraries and initial 3D nuclei segmentation.

Reminder: Run finishes when you see “Busy” turn into “Idle” and it might take couple of minutes or more depending on your computer. After you run this section, you can click and see initial nuclei segmentation on the Napari screen if wanted.


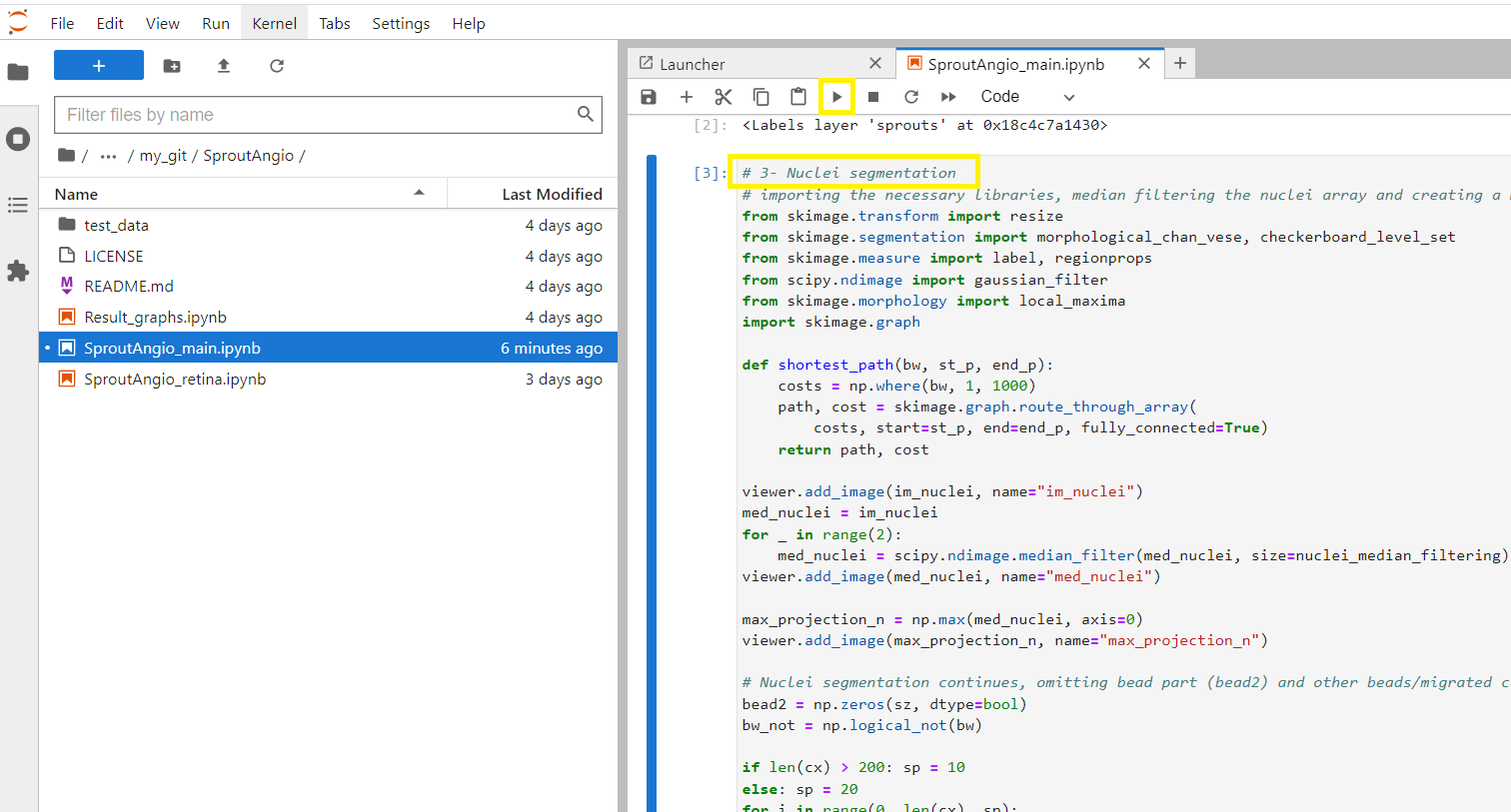


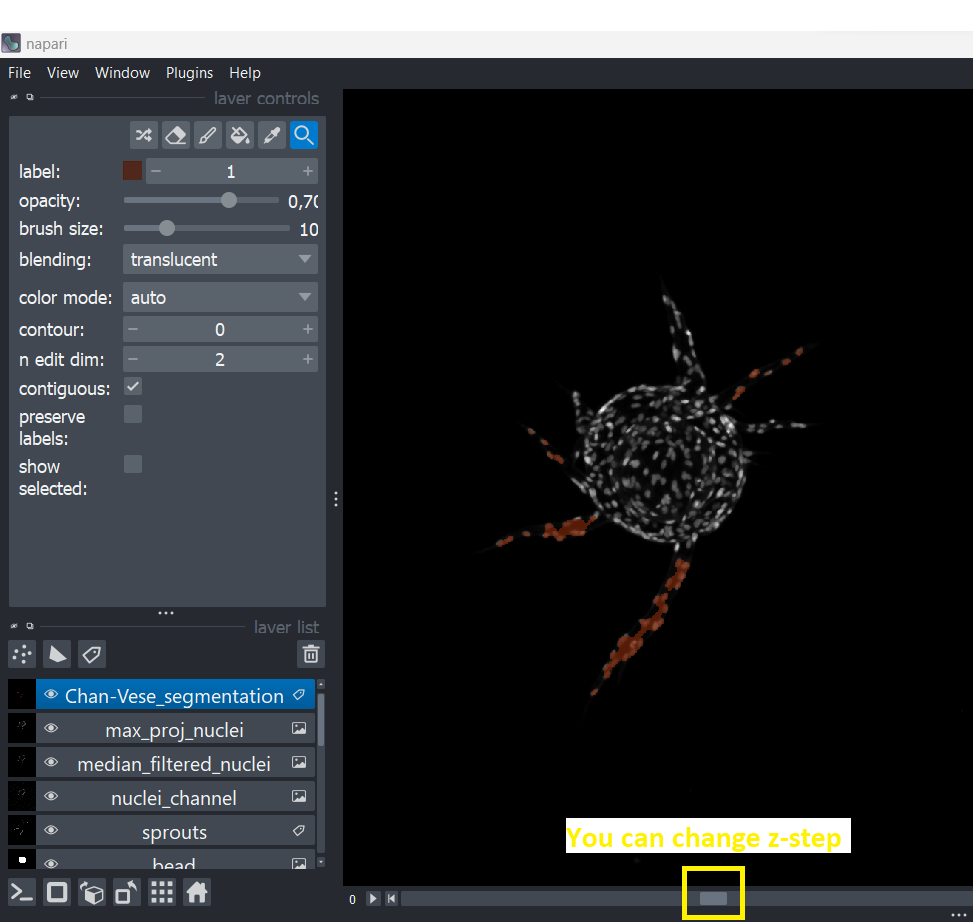


**8**. Now run the next two parts one by one. Lumen analysis and results section includes segmentation of under-segmented nuclei, lumen space analysis (regional width and paired nuclei distance analyses) and printing the results in the Jupyter notebook. The volumetric analysis section includes 3D sprout segmentation and volume measurements.


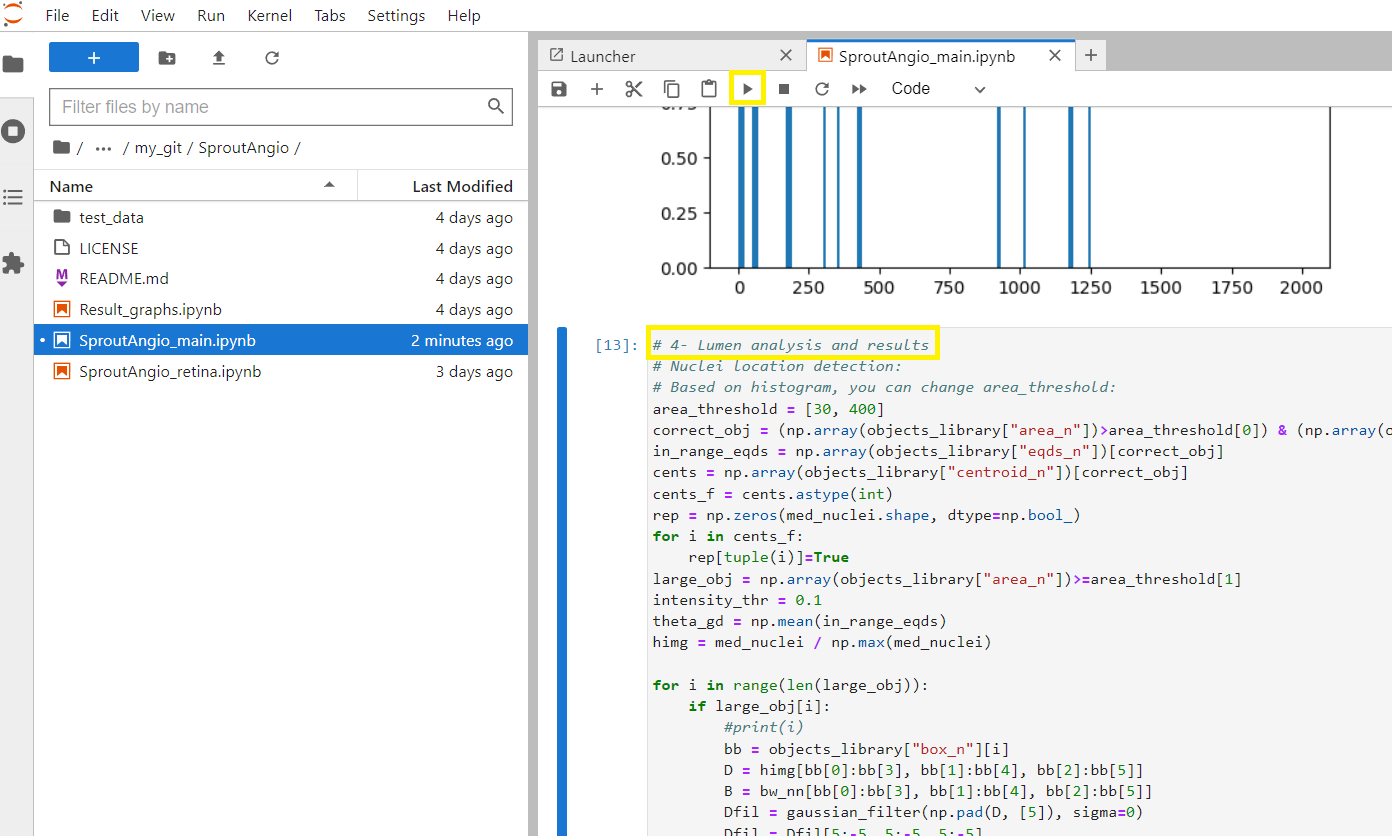


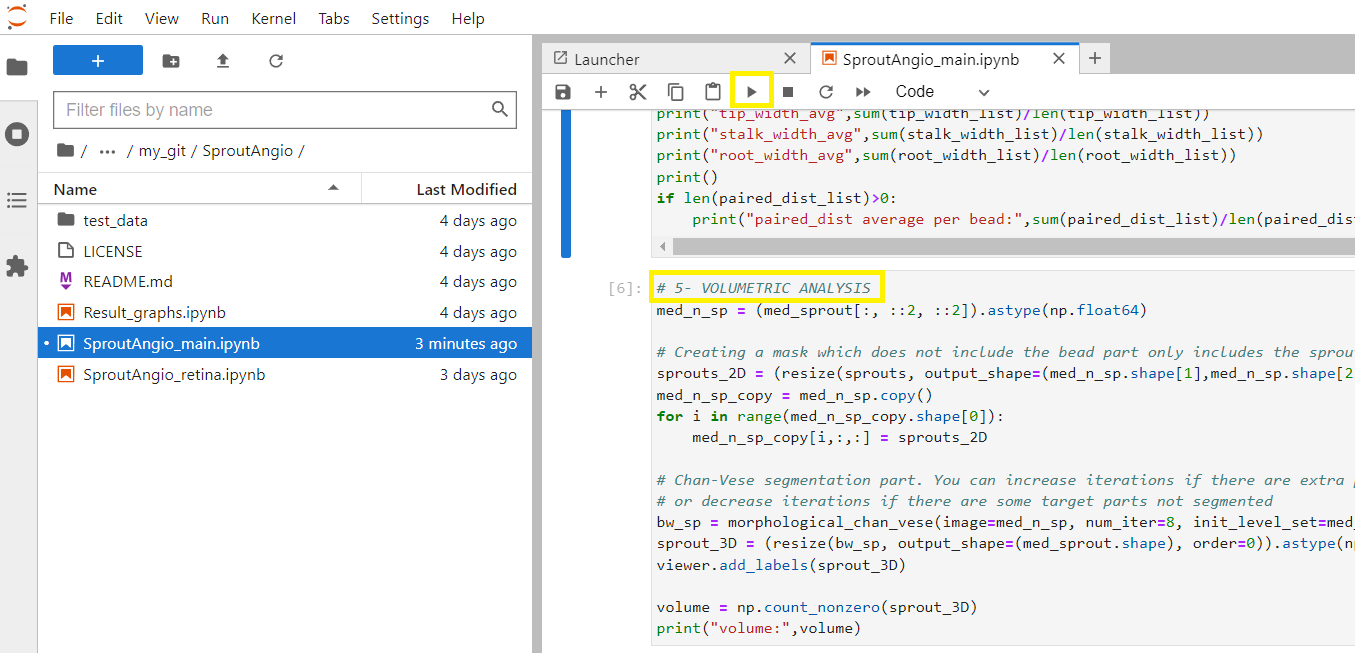


**9**. After both runs are finished, you can go to the Napari screen to see the result.

**10**. You can switch between the results by clicking on the eye symbol on the left.

Also, as the top image is 3Dsprout segmentation result, you can move in the z-dimension using the bottom tool.


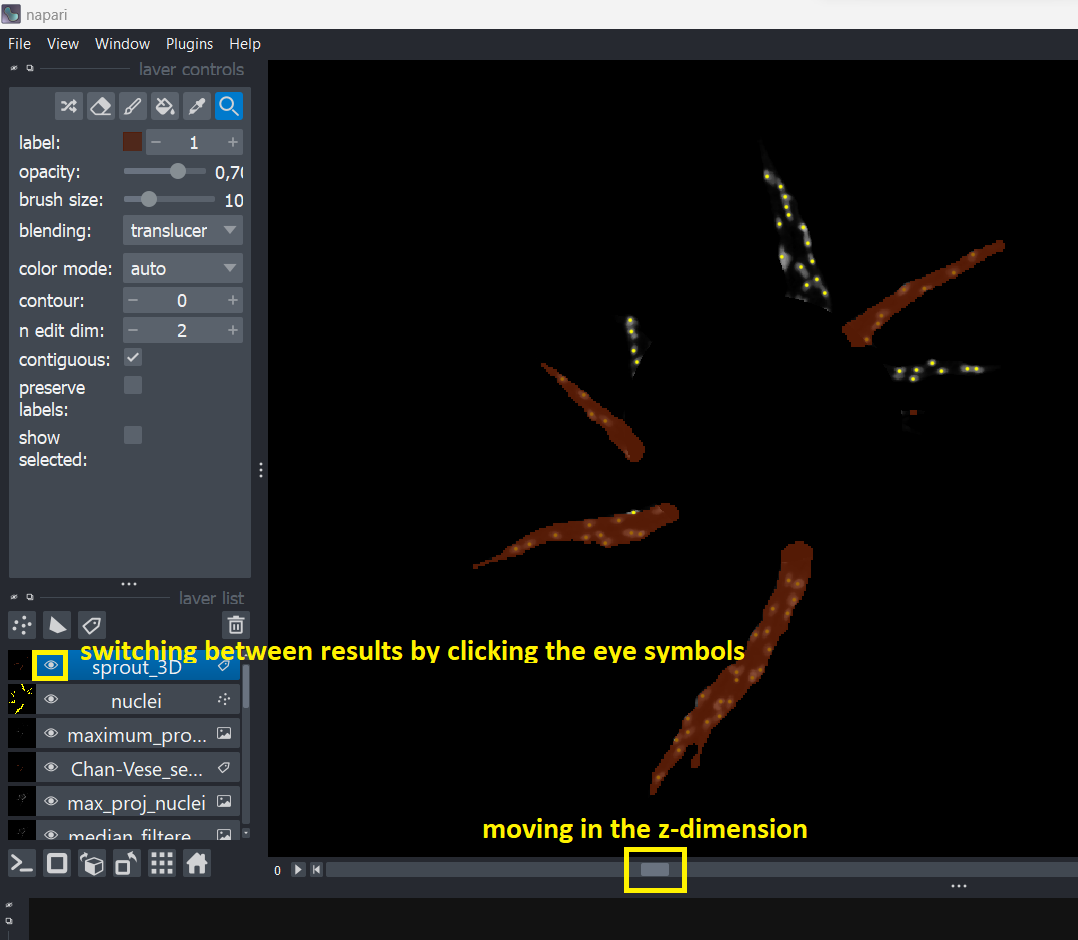


**11**. Since some of the results are in 3D such as “sprout_3D” and “Chan-Vese nuclei segmentation”, to see the 3D results, you can click “Toggle display” icon on the bottom left part.


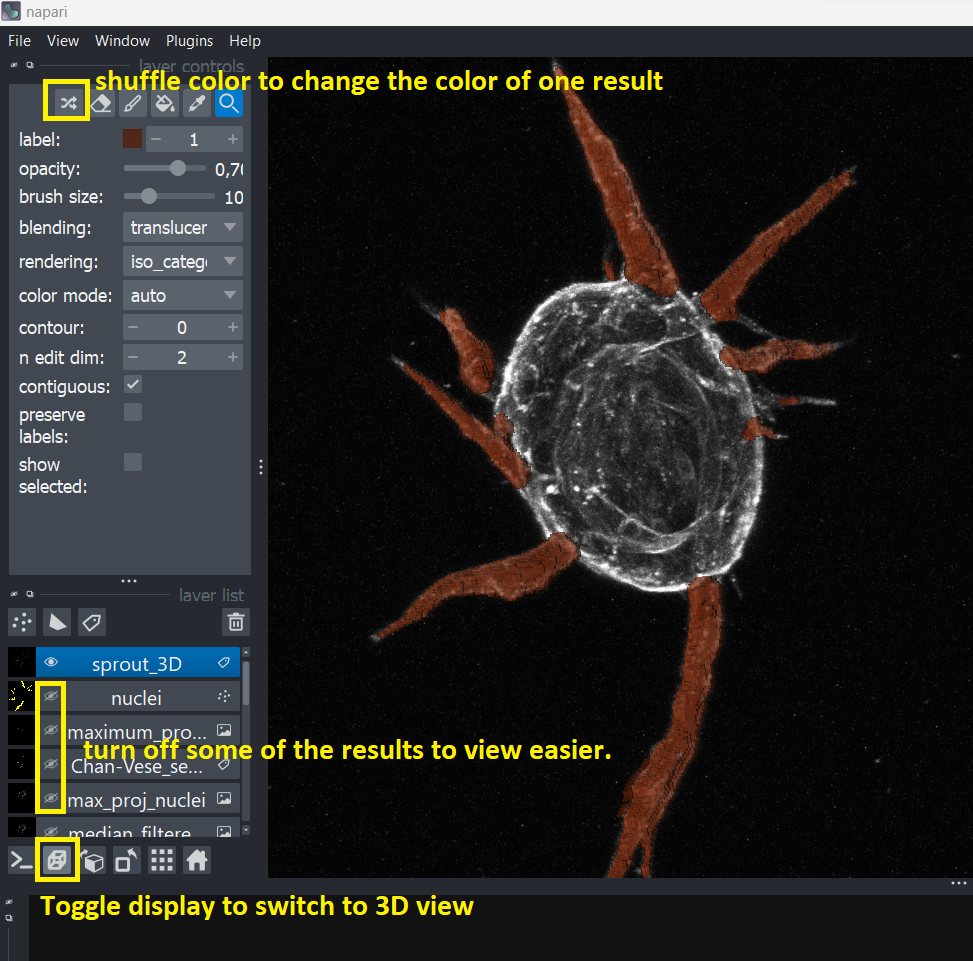


**12**. To simplify the view, you can make only a couple of selected results visible by clicking the eye icons. In 3D image such as this you can use your mouse to move the orientation. Also, in 3D view if you press “shift” in your keyboard then you can move the 3D image using your mouse without changing its orientation.

**13**. For more detailed information related to Napari viewer, you can check its tutorials: <https://napari.org/stable/tutorials/fundamentals/viewer.html>

**14**. When you switch to the SproutAngio JupyterLab tab, you can see that the results are written on the right side after section 4 run and the paired nuclei distance result image was saved as “analyzed_image.png” on the left in the SproutAngio folder.


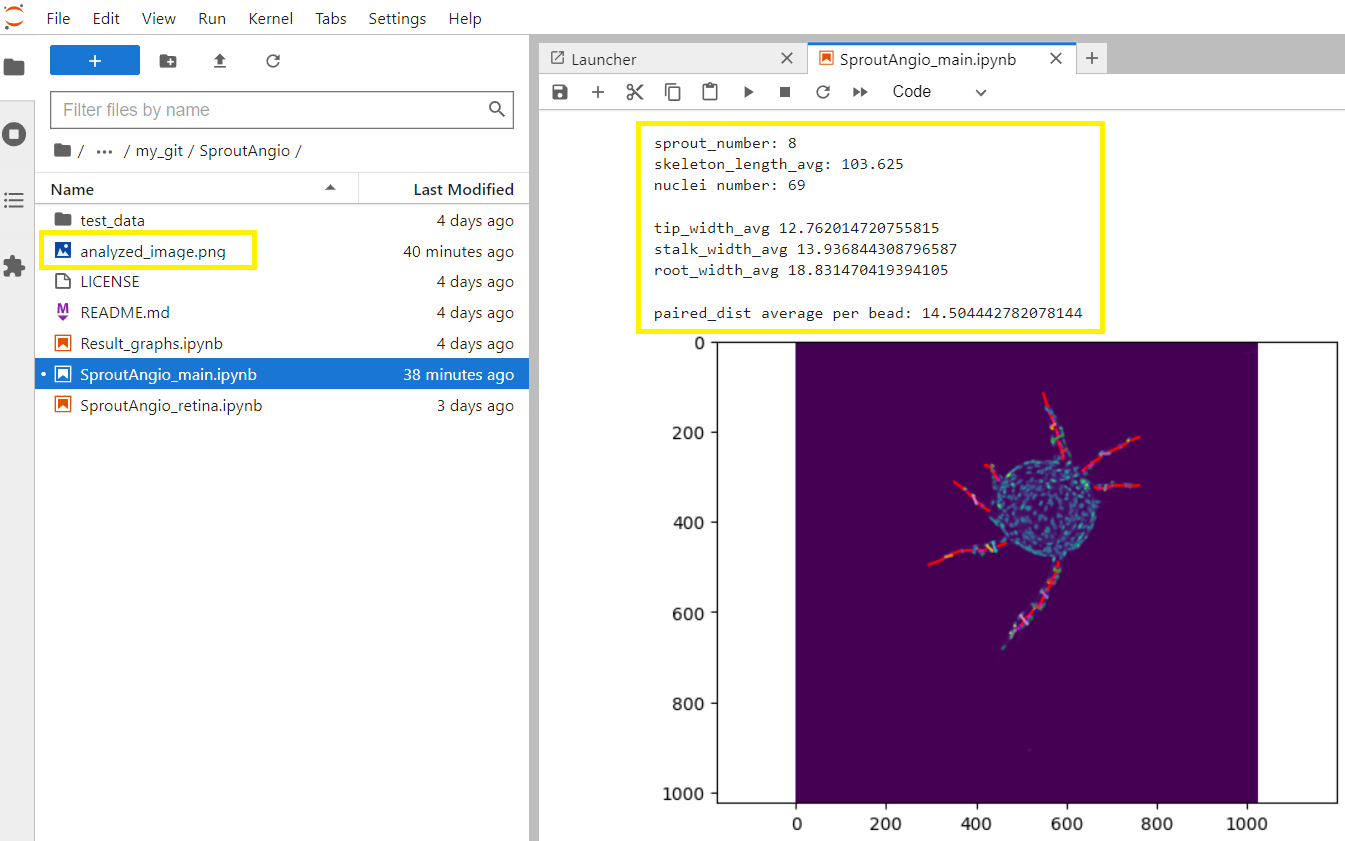


**15**. You can easily change the parameters if needed for the filtering and % radius of the detected bead from the first section you ran.

As examples:

1. When you increase/decrease the rad_ it will increase/decrease the detected bead area. Therefore, it will change the sprout segmentation. E.g., test by changing median_filtering = (6,6,6) and rad_ = 1.1.
2. If you think the sprouts are not separated well enough, you can increase the radius and improve the sprout segmentation.


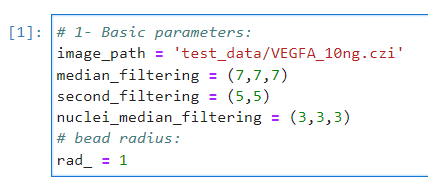


**16**. Another important section which you can easily change is the nuclei segmentation part (section #3). Depending on your image, if you are not satisfied with the nuclei segmentation, you can change the num_iter.

As examples:

1. You have nuclei that are not included in the segmentation, decrease the iterations (num_iter), as this changes the number of repetition steps for the algorithm to get a better segmentation.
2. You have parts included in the nuclei segmentation which are not true nuclei, improve the analysis by increasing the num_iter value. Note that for num_iter values you can only use integers.


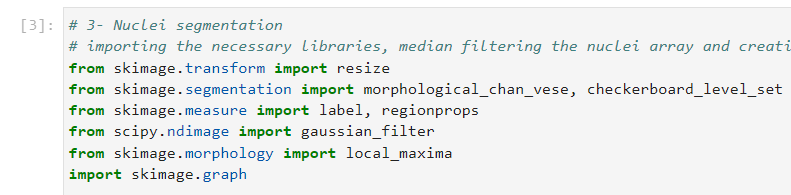


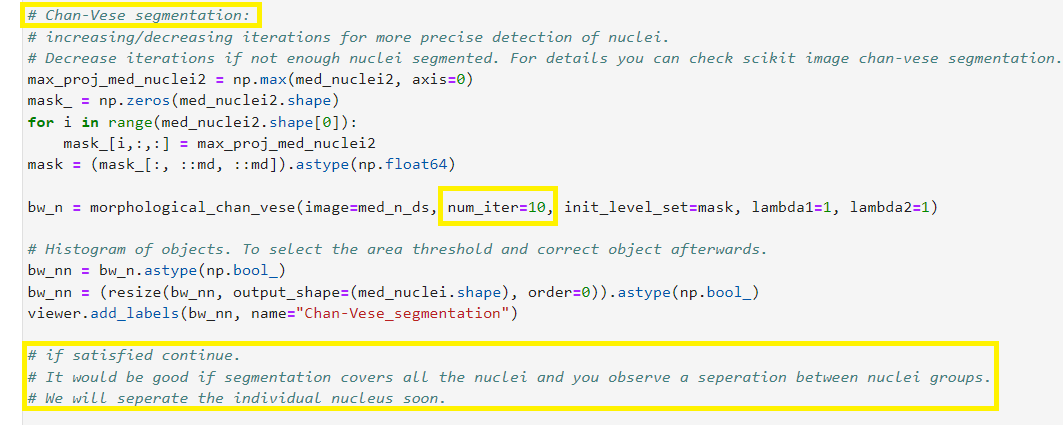


**17**. For the segmentation of under-segmented nuclei and following calculations, you can change the area threshold values if needed (section #4 Lumen analysis and results). After you run the previous section (section #3), you will get a histogram for the area of the segmented nuclei. Area_threshold value in section #4 defines the threshold for the correct nuclei area.


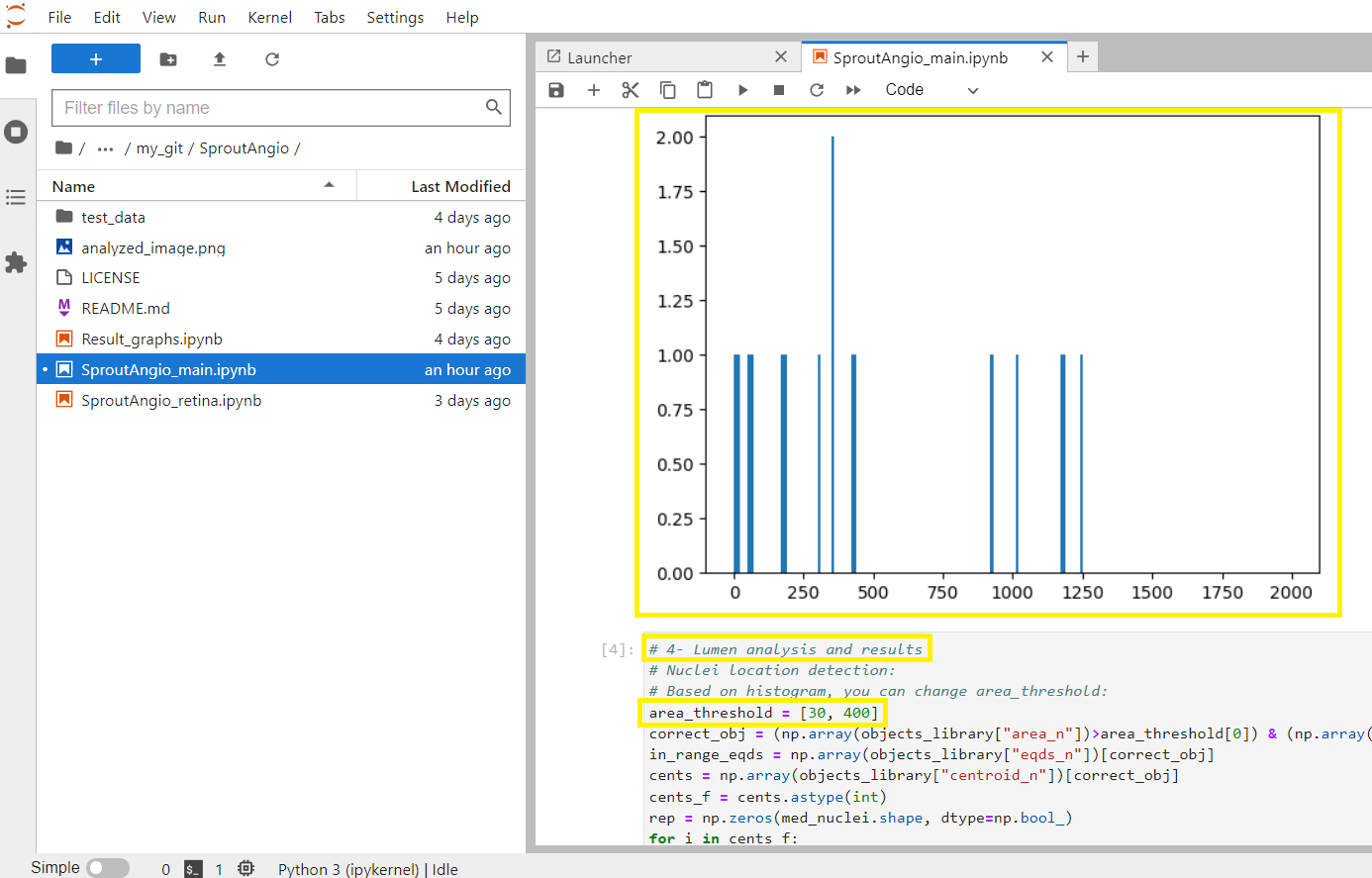


Note! Depending on your data, you can check the histogram and decide the correct area threshold for individual nucleus. For example, cell size might be different between your cell lines.

**18**. For the sprout width measurement (section #4), you can change the part of the sprout from which the width is measured. By default, SproutAngio tool measures the width of 25%, 50%, 75% parts of the segmented sprouts. However, you can change this if you want to get measurements from different parts of the sprouts depending on your experiment.


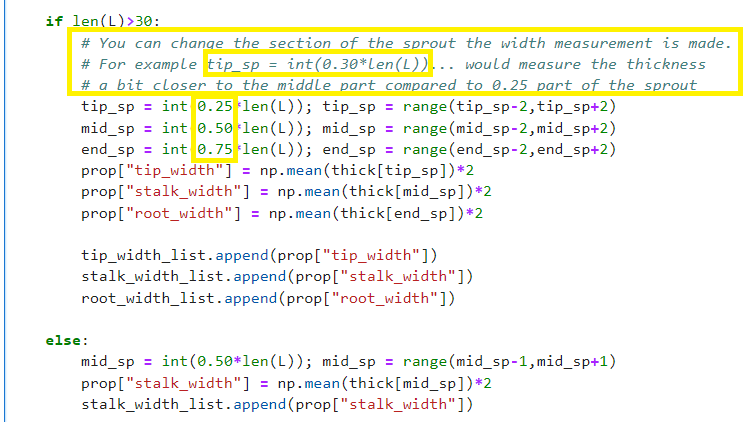


**19**. For the volume measurement, similar to the nuclei segmentation part, you can change the num_iter value to get better segmentation.

As examples:

1. If you have areas missing in the segmentation, you can decrease the num_iter integer value.
2. If you have regions which are not sprouts but still segmented as sprout volume, then you can increase the num_iter to improve the segmentation.


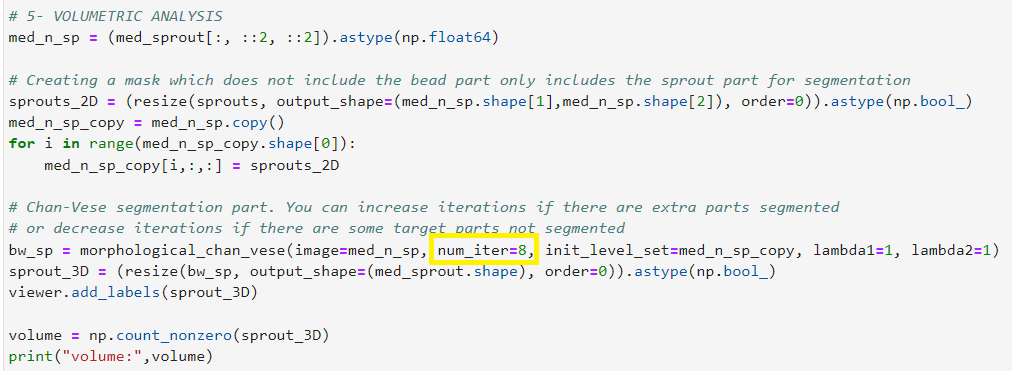


Note! In this user’s guide, we cover the most useful parameters that you can modify. However, you can also play with different parts of the sections to familiarize yourself with the code. There are more explanations related to the sections on the code itself.

**20**. The last part of SproutAngio_main is for writing the data into excel file. After you run this part (section #6), there will be an excel file created in the SproutAngio folder you created in the beginning on your desktop. Also, an image of the paired nuclei distance analysis used for studying lumens is automatically saved in the same folder as “analyzed_image.png”.


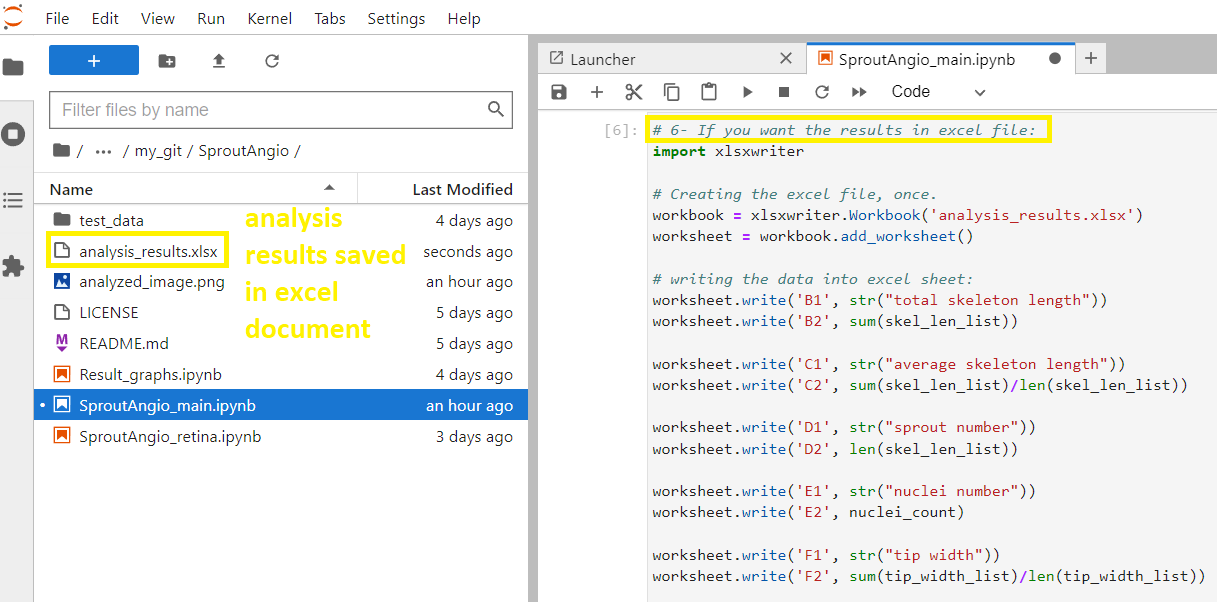


**21**. Now, to turn off the analysis platform and the SproutAngio Jupyter tab, you need to close both the Jupyter notebook tab and the kernel (marked in the figure below in yellow). First, select on the left the “running terminals and kernels” and “shut down all”.


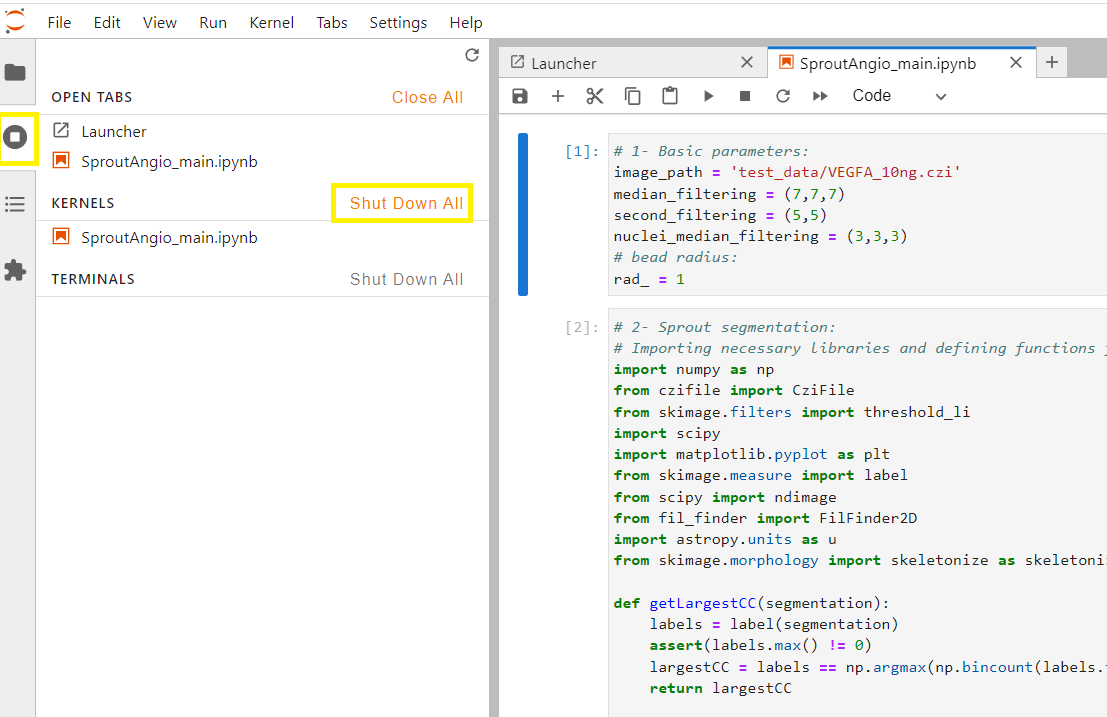


**22**. Close the SproutAngio_main tab.


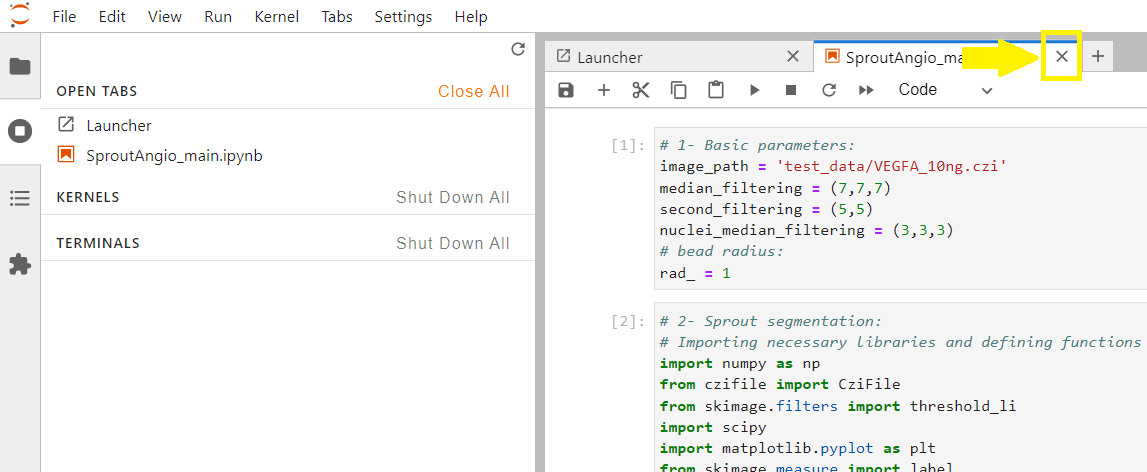


**23**. When you work on your dataset, first optimize your analysis using SproutAngio_main tool as demonstrated in this guide. After you finish optimizing your parameters, if you want to run multiple files at once, then launch the “SproutAngio_multiple_run.ipynb” by double click opening it instead of “SproutAngio_main.ipynb”. Change the parameters you optimized previously, here. Note that there is only one section here and after you run it, it will take a while for the run to be completed depending on the number of images you analyze.


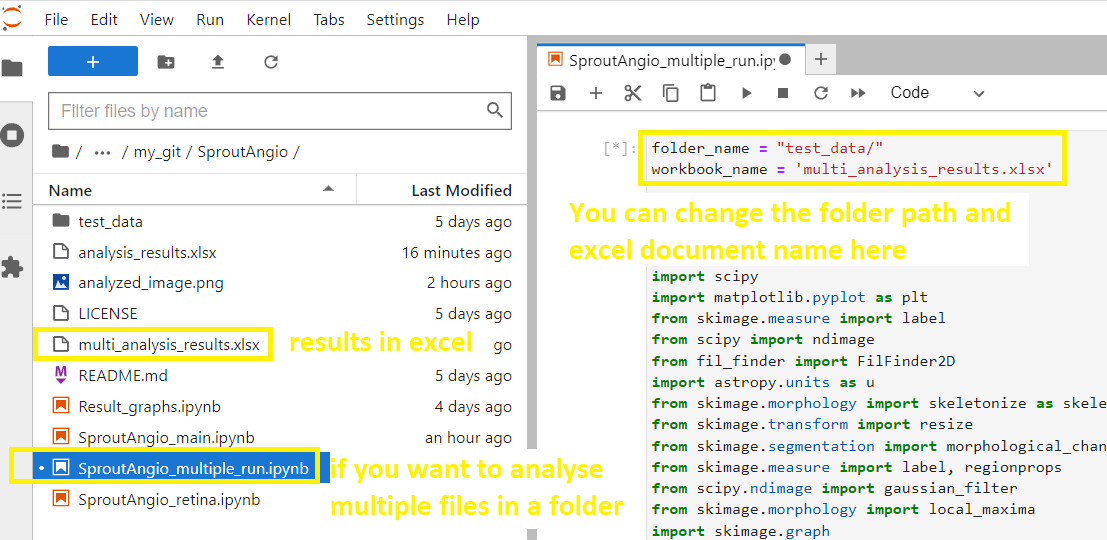


**C. RETINA ANALYSIS:**

**1**. To start using the SproutAngio_retina, double click the SproutAngio_retina file on the left (yellow rectangular in the figure below). Then, run the first section which imports the necessary libraries such as Skan Skeleton Analysi library and define necessary functions to exclude the central part during image processing.


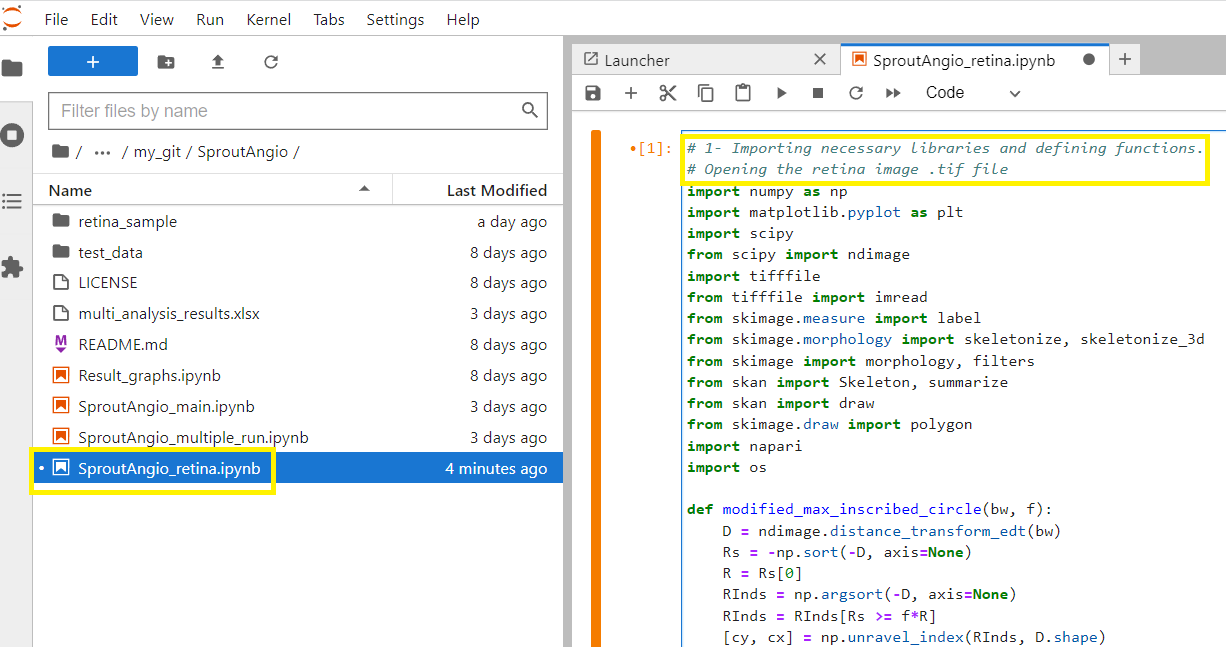


**2.** Next, run the second section. This section first reads all the files in defined “retina_sample” folder. If you create another folder in this file, simply change the file_path to open the new folder.

In our retina_sample folder there is only 1 retina image file. However, if you have multiple files, you can change to the next image file by simply changing the “folder_names[0]” into “folder_names[1]”. Read the instructions above the code for more information.


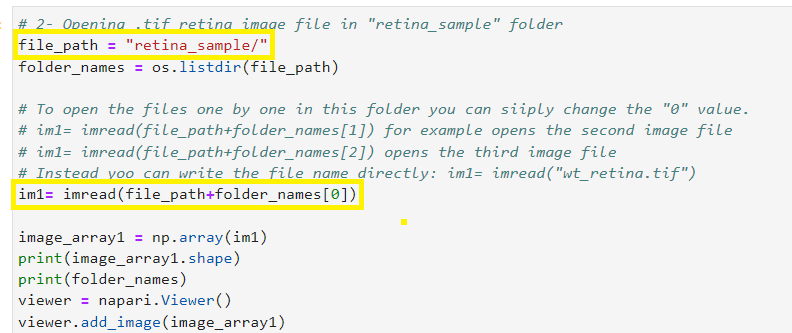


**3.** After running the previous section, Napari viewer tab should open in your computer. Open the Napari viewer.


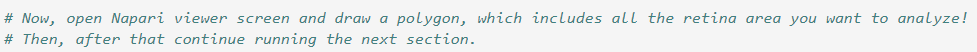


Now click the “new shapes layer” as shown below image to create a new shape layer to draw the region of interest.


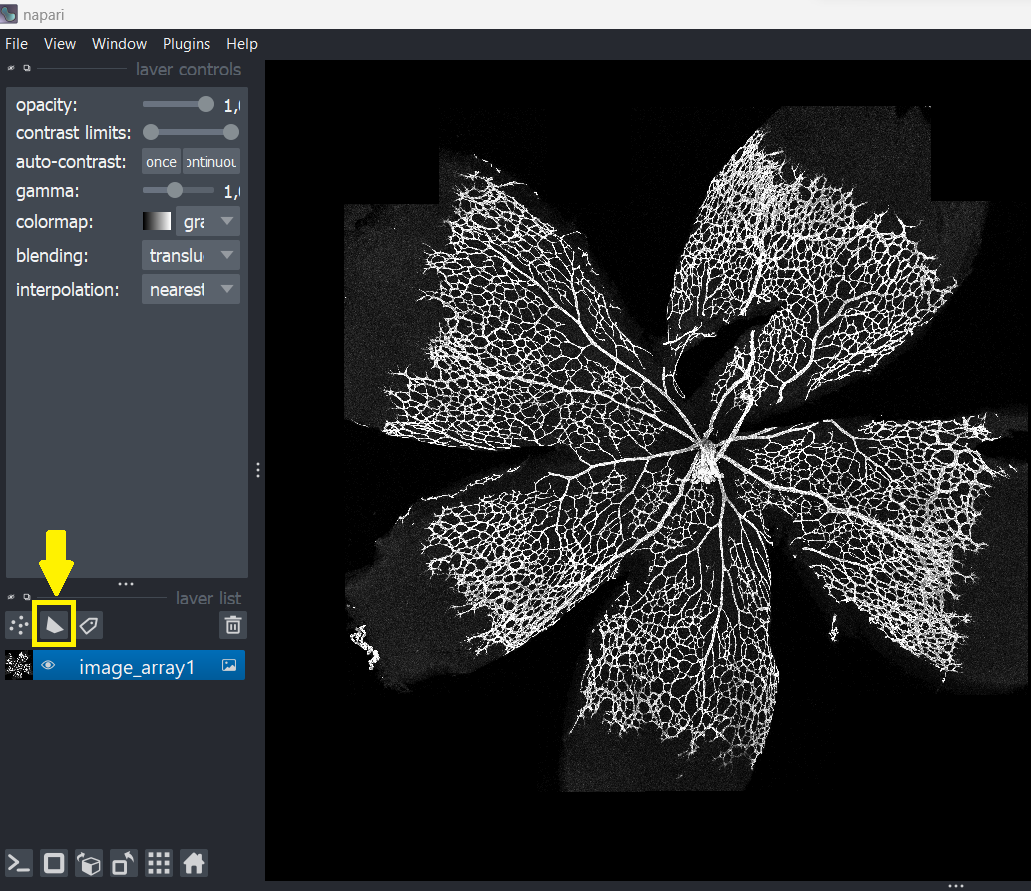


**4.** When “Shapes layer” is selected, change the color into something you can easily see/ differentiate during drawing on this black and white image. After that choose “add polygon” to start drawing the region of interest.


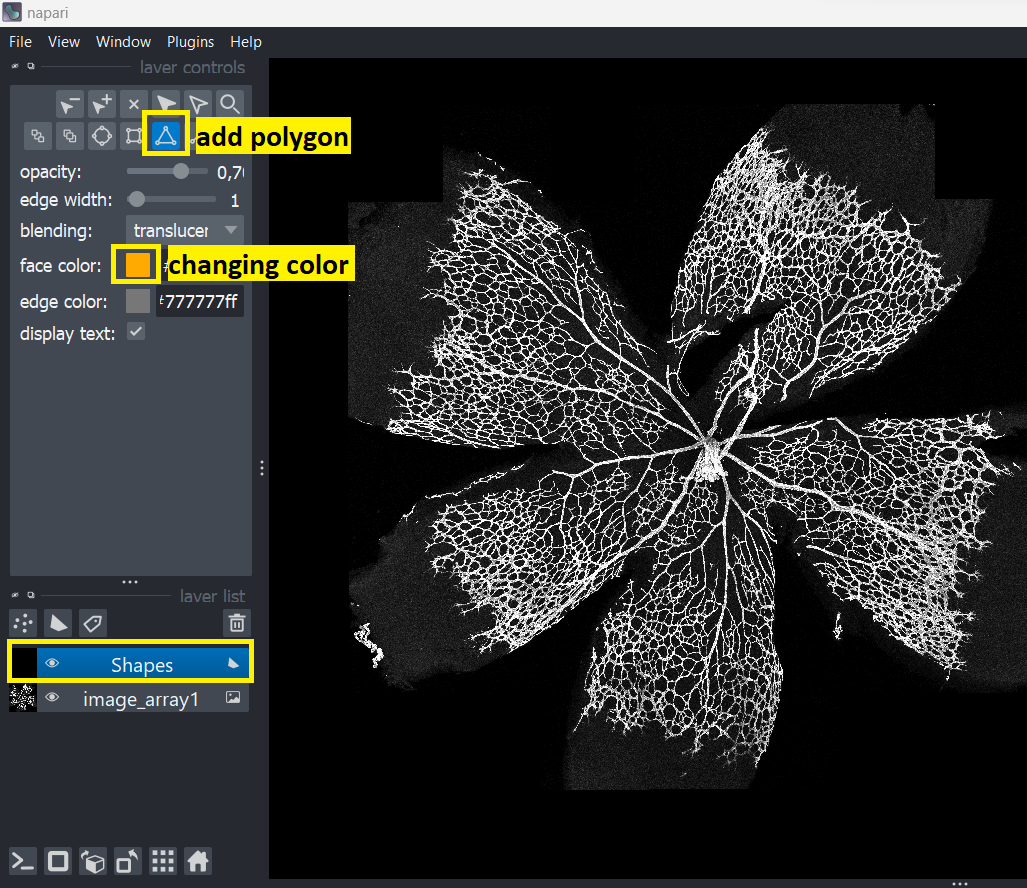


**5.** Now draw around the retina area by clicking one by one, on your last click you either use double clicking or “space” key on your keyboard to stop drawing. If you make a mistake you have multiple options shown in below figures.


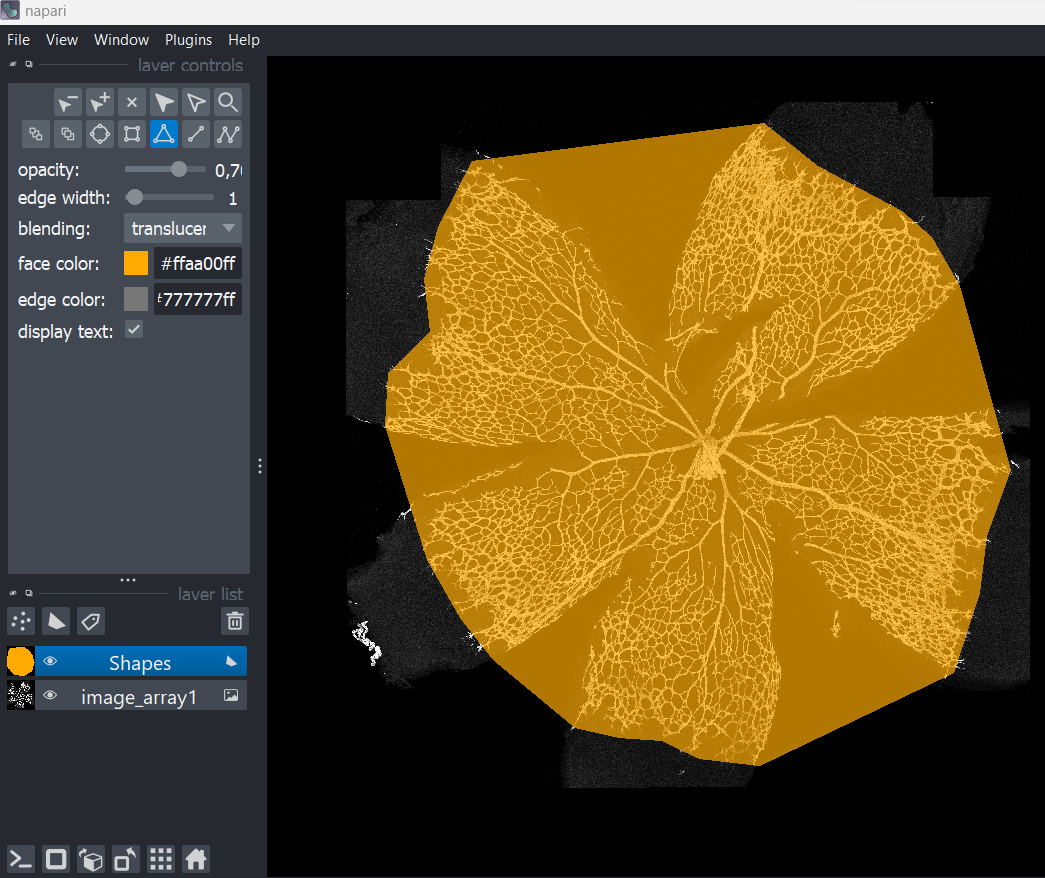


If you make a mistake, you can click to the selection tool and move each individual dot you have put so far.


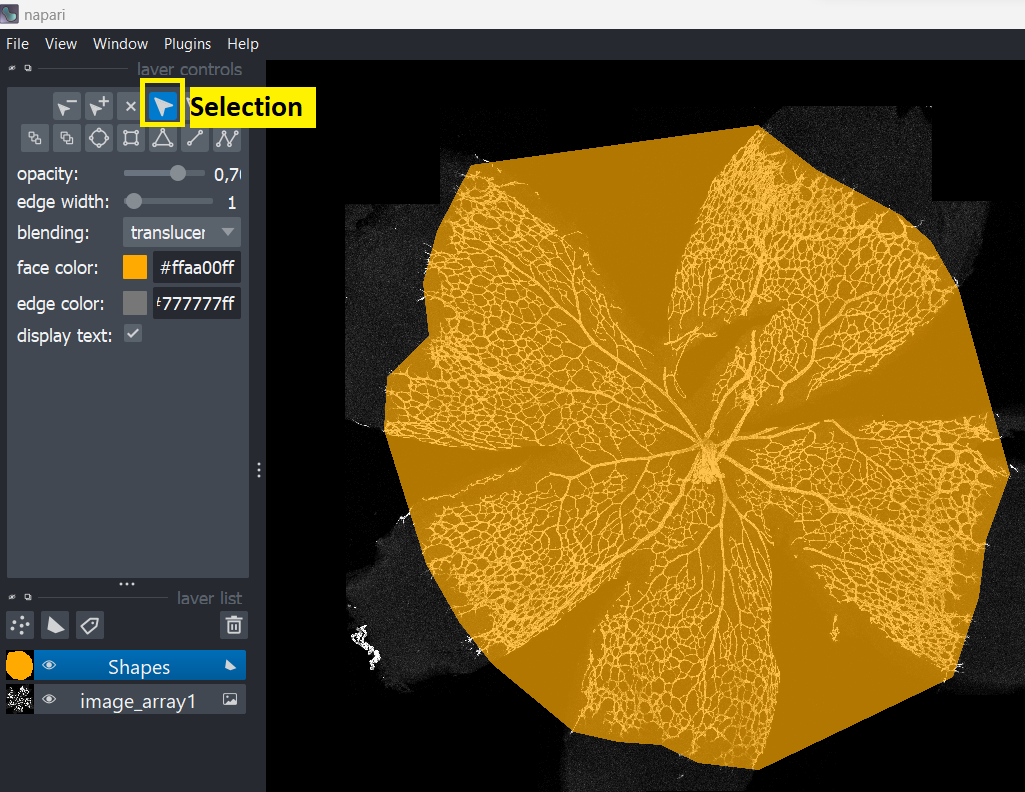


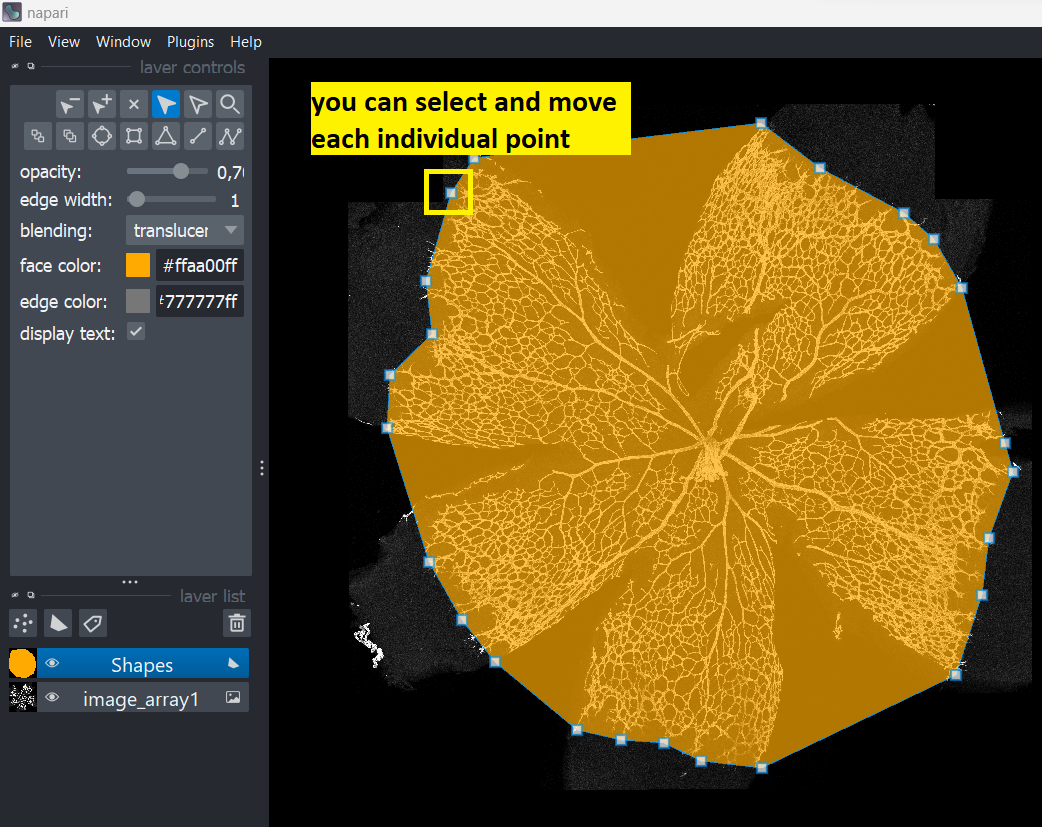


Or you can delete the whole shape and draw a new one:


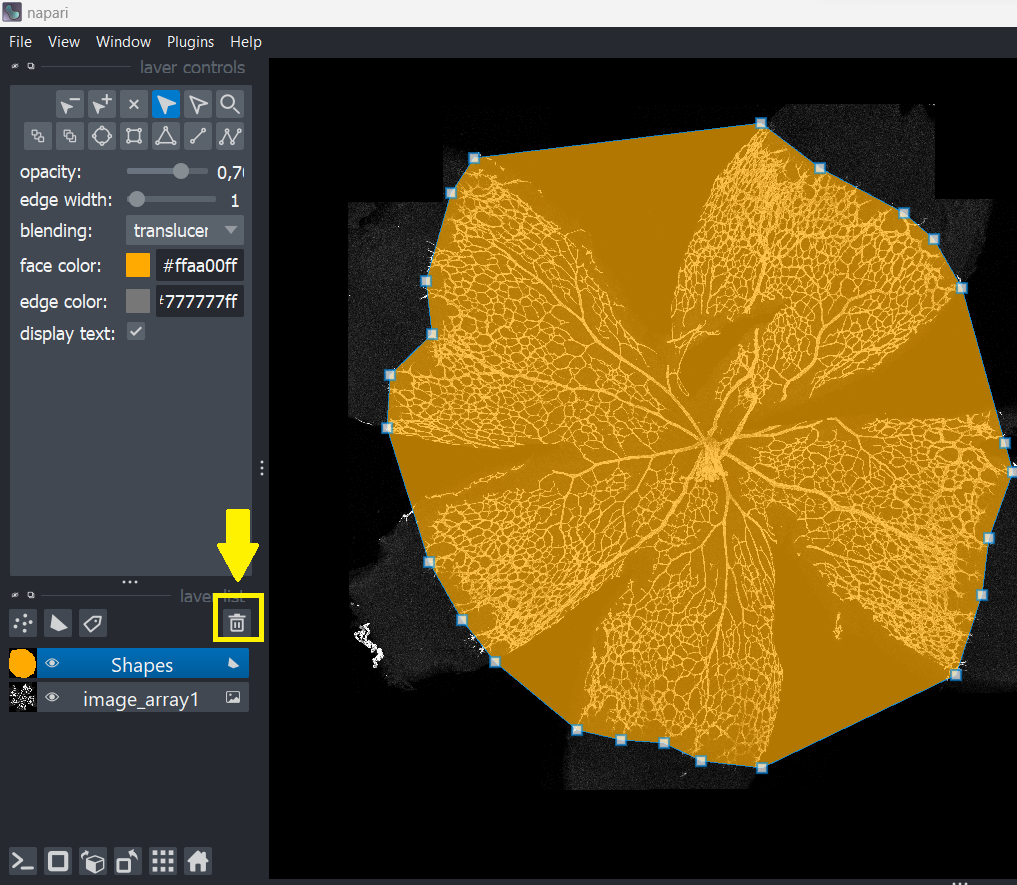


**6.** After you finish drawing your region of interest, in our case it is the whole retina, you can return to Jupyter notebook and run the next section. This section’s purpose here is to get the drawn points into an array to use in the pipeline.


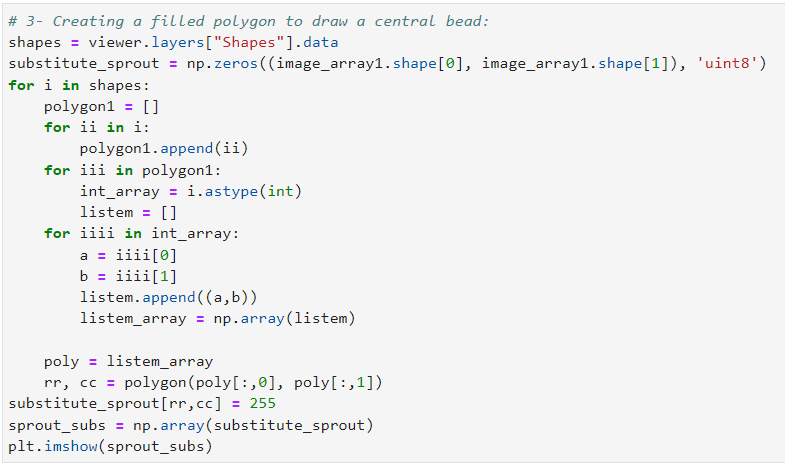


**7.** After the last section’s run finishes, run the next section. This part is all about the exclusion of user defined central part. You can easily change the radius you want to remove. Here, we will use 0.3 R, 0.5 R and 0.7 R radius exclusion.


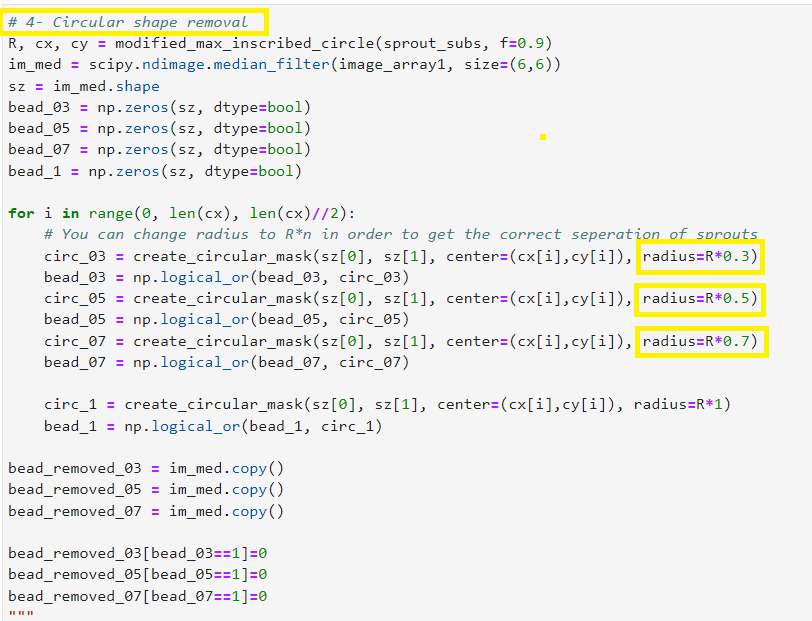


After the run ends (when you see Idle instead of Busy on the left bottom part of the notebook), you can open Napari viewer to check the images. Remember to switch between the images by clicking the eye symbol as shown below.


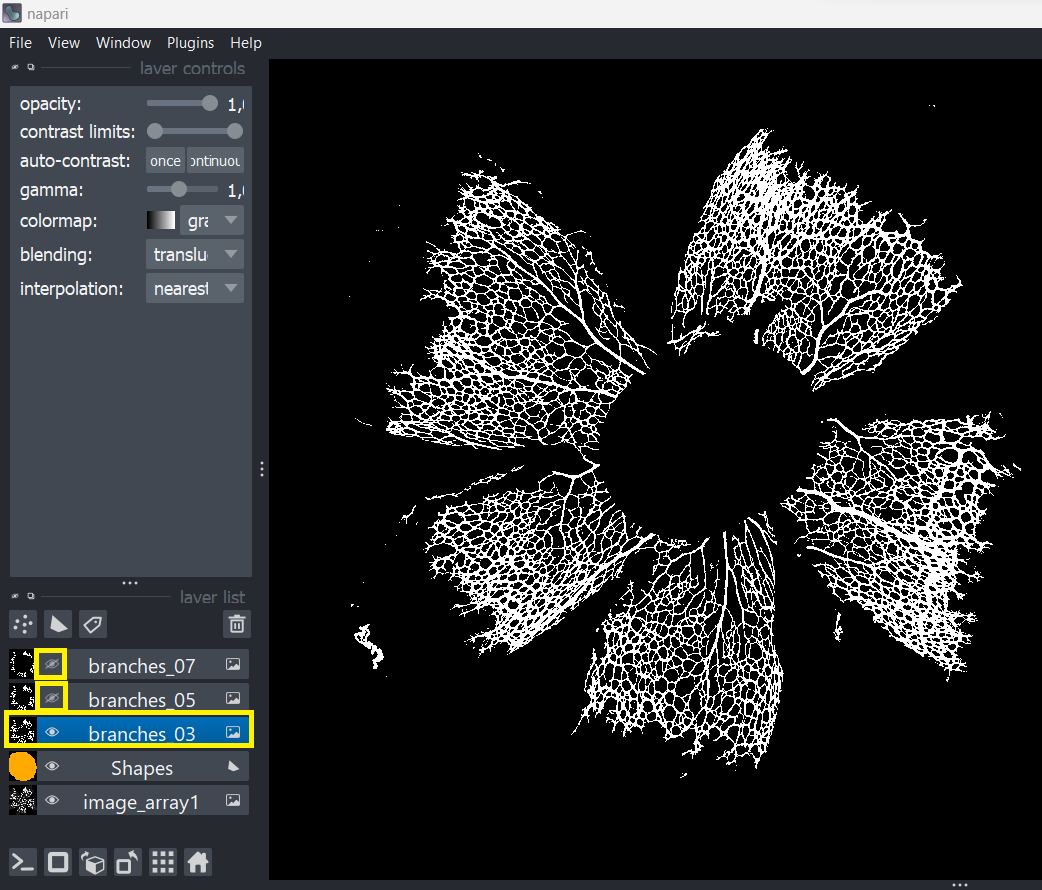


**8.** Next section is branch length analysis. Run this section. You will get total skeleton length (pixels), total branch number and long branch number results printed. Also, you will see three histograms of branch lengths for 0.3R, 0.5R, 0.7R radius selections. At the end of the run, you can also check the napari viewer to see the skeletonization of the branches. We will explain these steps in detail below.


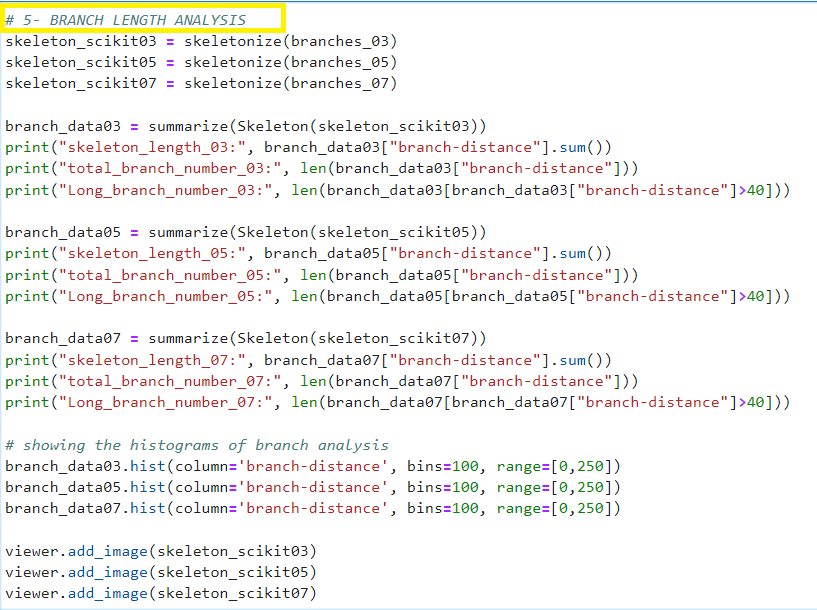


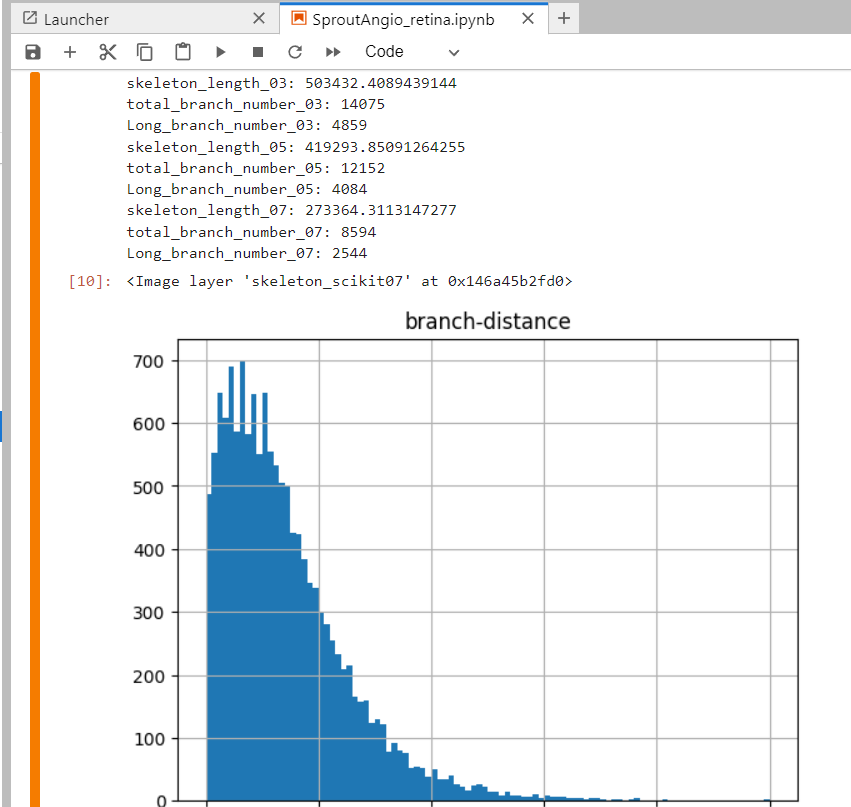


**9.** On histograms, x-axis shows the branch length (pixels) and y-axis shows the number of branches. Long branch number results give here the branch number which are longer than 40 pixels. However you can easily change this in the code. For example looking at the histogram, if you think you want to use 50 pixel length as threshold here, to get the number of branches longer than 50 px:


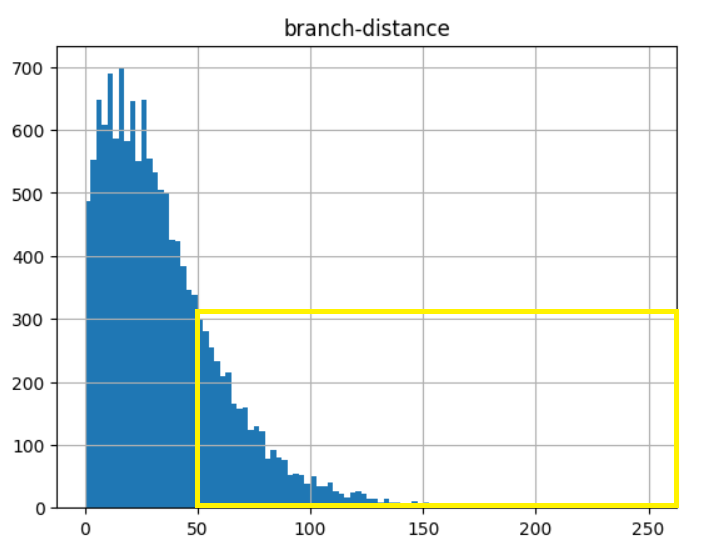


Simply change these “40” values into “50”:


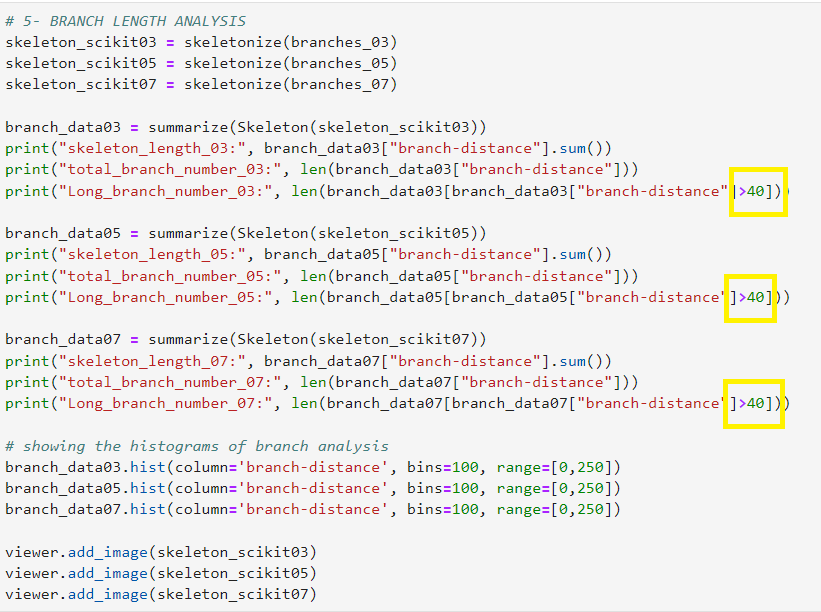


**10.** You can look at the Napari viewer to check the skeletonizations. You can open the same branch and skeleton to look at them together. Here we will choose 0.3 R branch and its skeleton together:


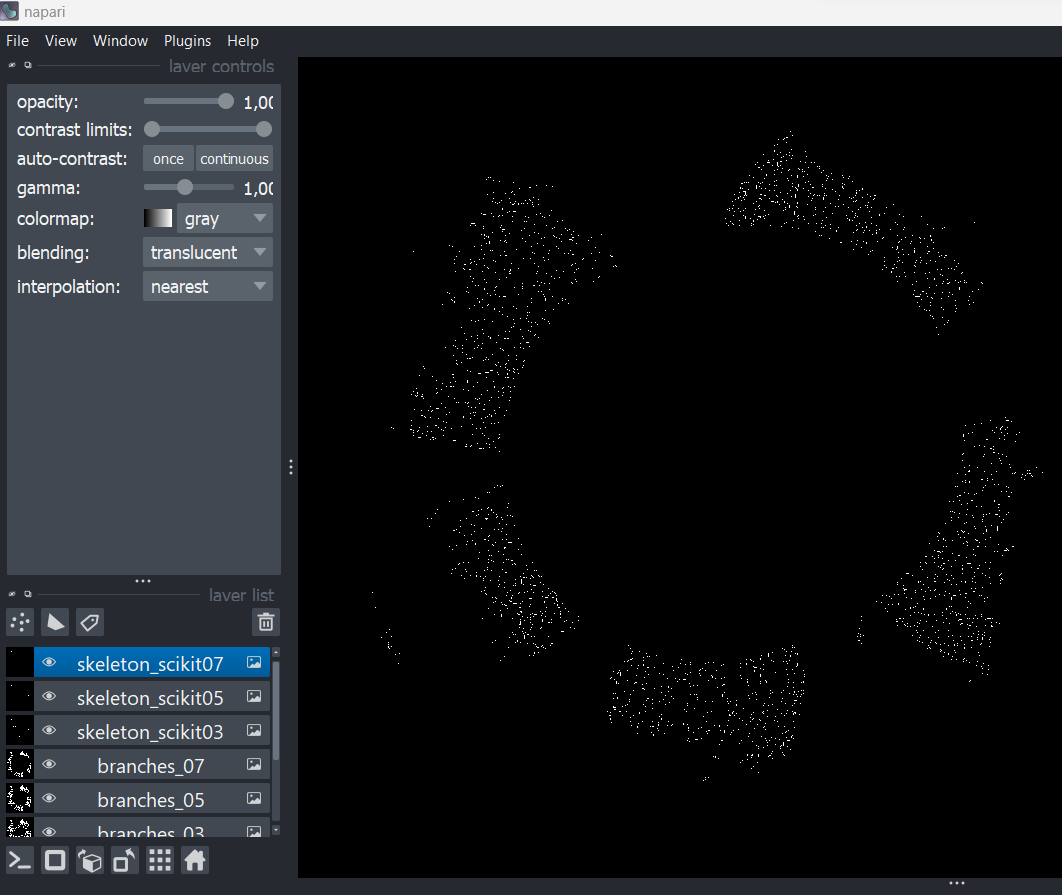


First, only select these two from the eye symbol:


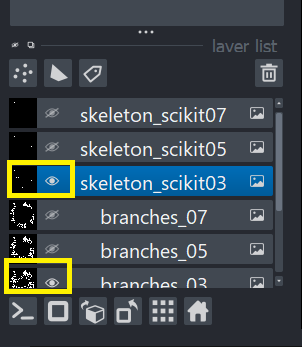


Then select the skeleton channel by simply clicking on it, and change the color of skeleton something visible and reduce the opacity. We used 0,60 for opacity here. Then zoom in using your mouse scroll to see in detail.


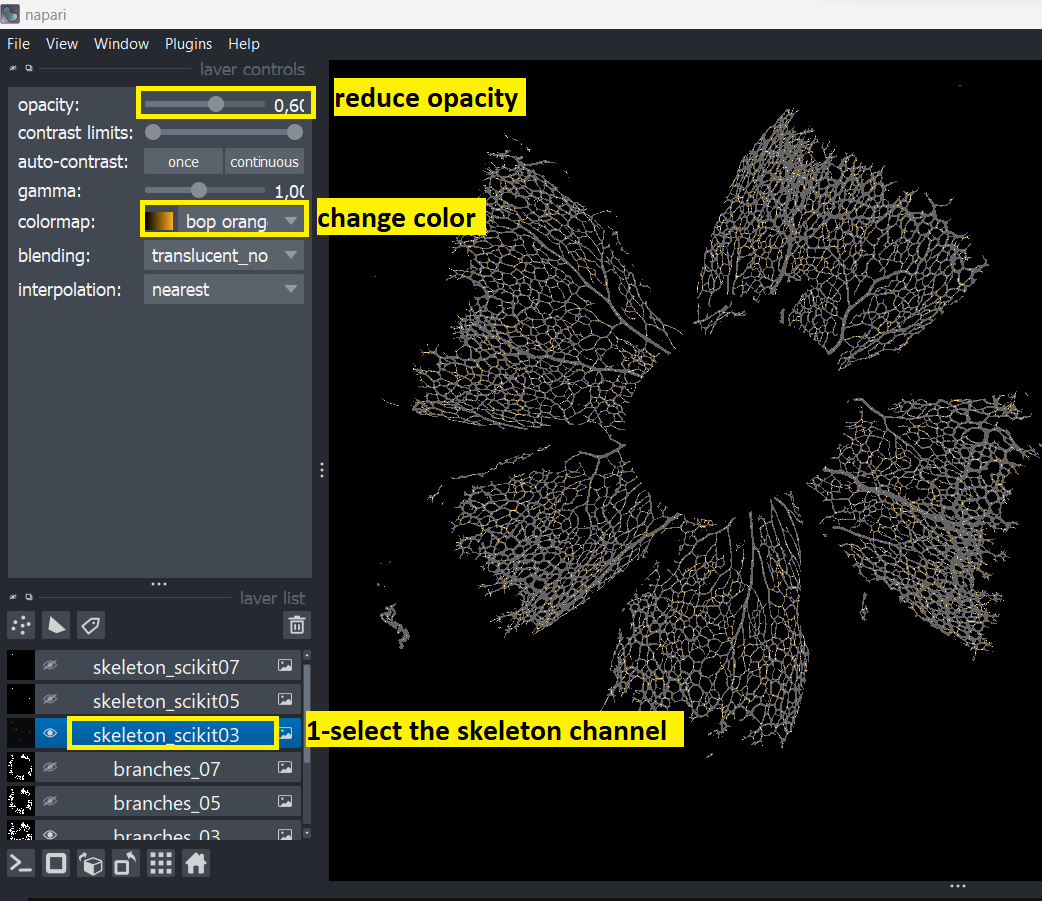


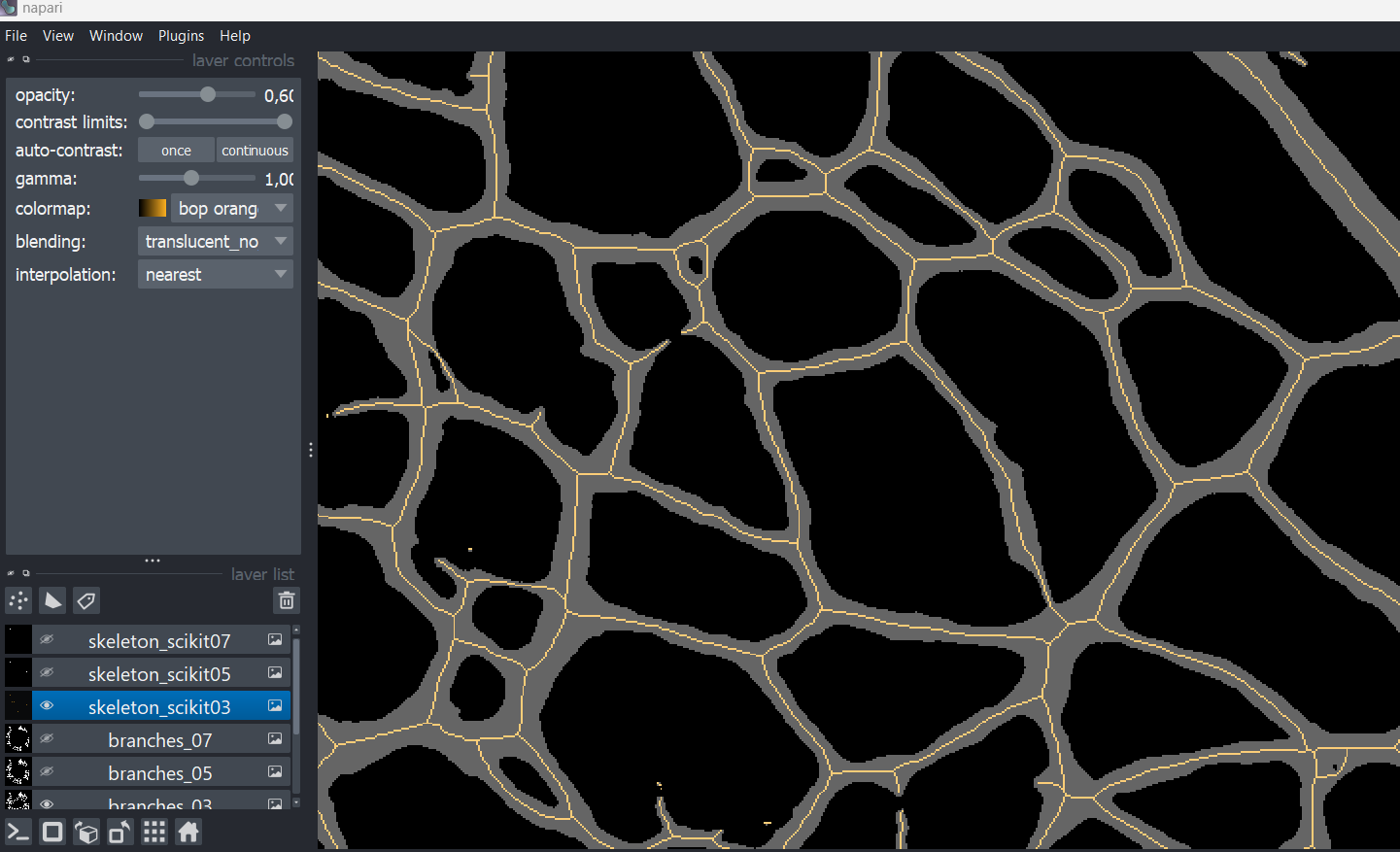


**11.** Now run the last part of the code. Output of this section gives you vessel density % area measurements and radial expansion result.


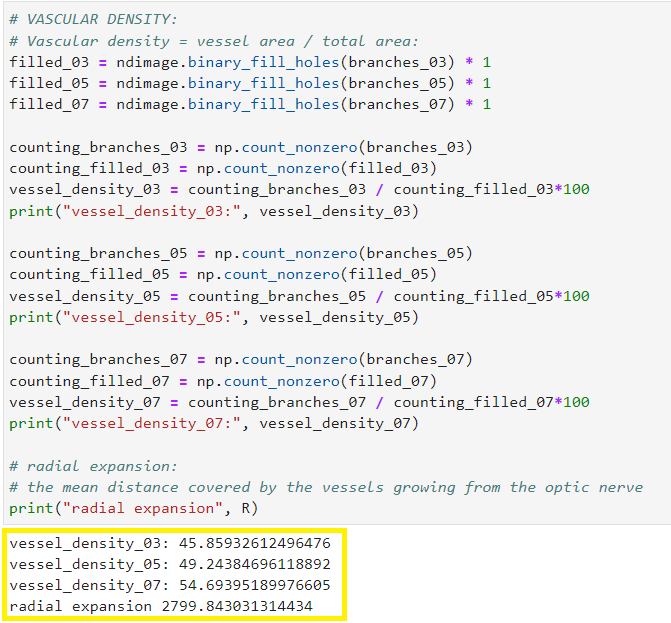


**12.** Remember to turn off JupyterLab!


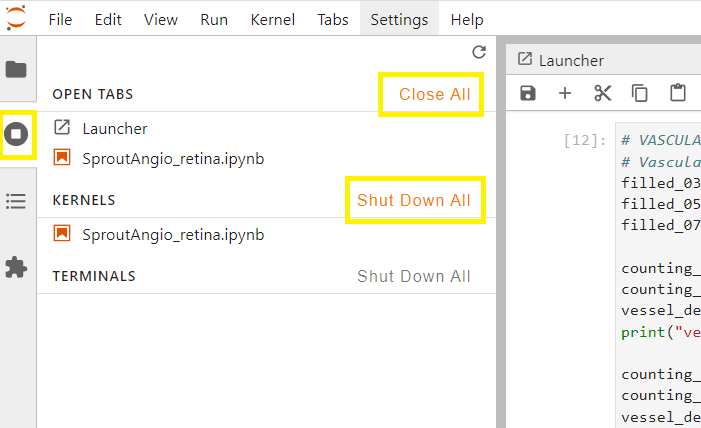


**D. RESULT GRAPHS AND STATISTICAL ANALYSIS:**

**1**. The purpose of this part is to help you do statistical analysis and draw graphs in the same platform easily. Open “Result_graphs” file by double clicking on it.


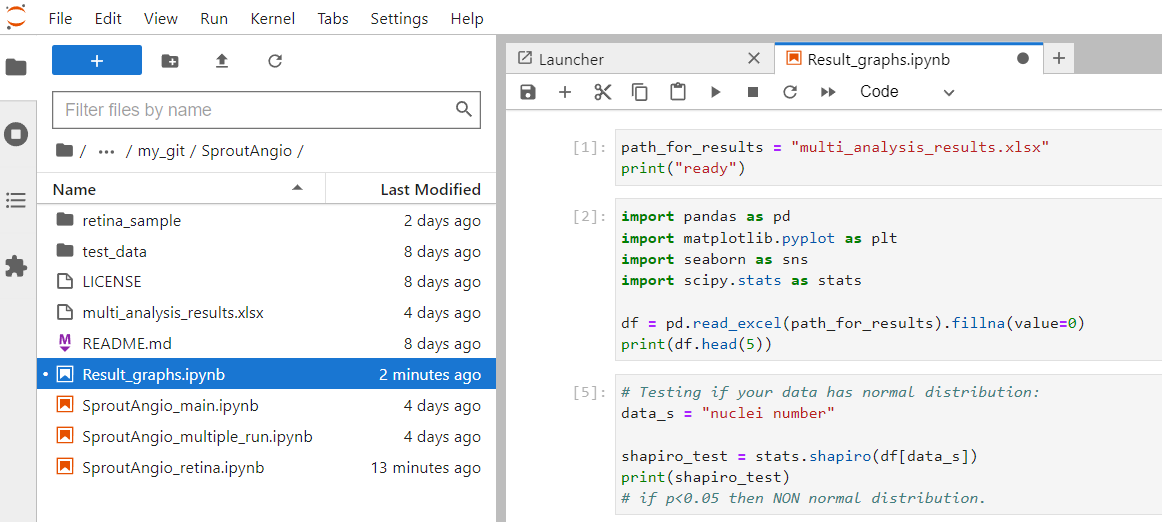


**2.** If you followed the guide so far, you already created multi_analysis_results excel document during part B Using the tool, step 23. We will not work on it as an example.

Run the first section to define the file path, which is the excel document we created previously here:


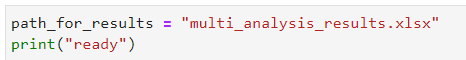


**3.** Run the next section. It will print the results in the excel file for you to check.


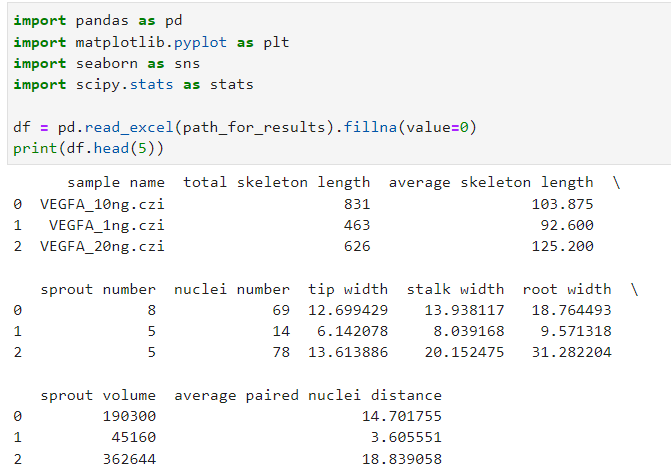


**4.** Run the next section. The purpose of this part is to check your data to see if it is normally distributed or not.


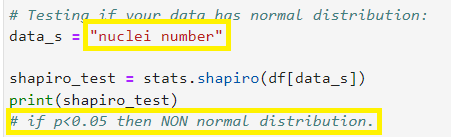


**5.** Run the next section. It draws a correlation heatmap. You can delete or add the parameters you want to include by changing inside the part shown in yellow rectangular below:


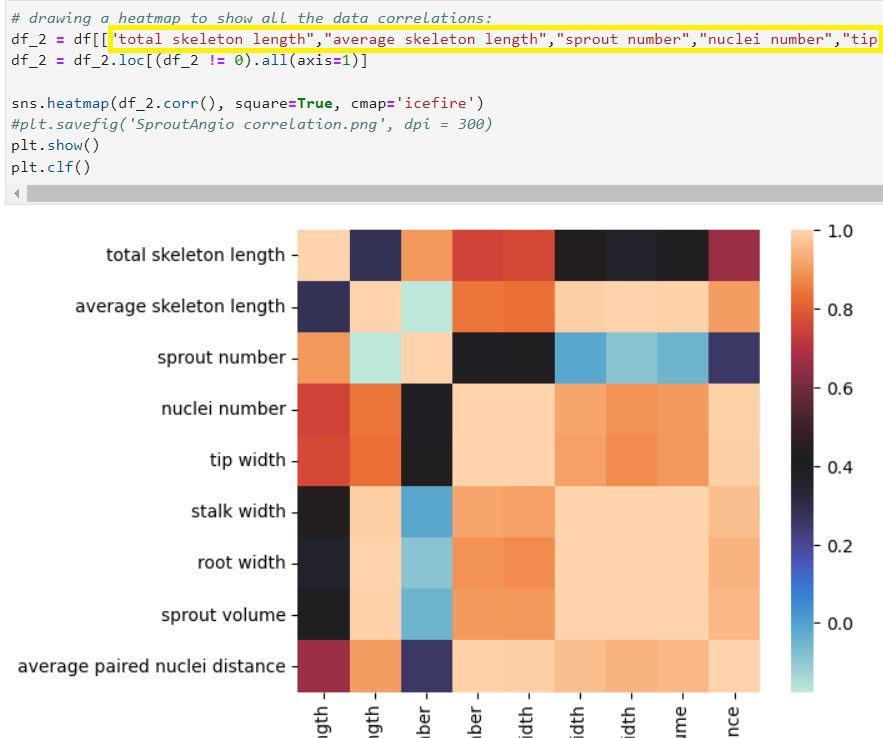


**6.** Run the next section. This section draws individual correlation graphs between two parameter results you choose. Here it shows a graph correlation of nuclei number and total skeleton length. Also, this part, removes the “0” data points, not including them in the correlation calculation.


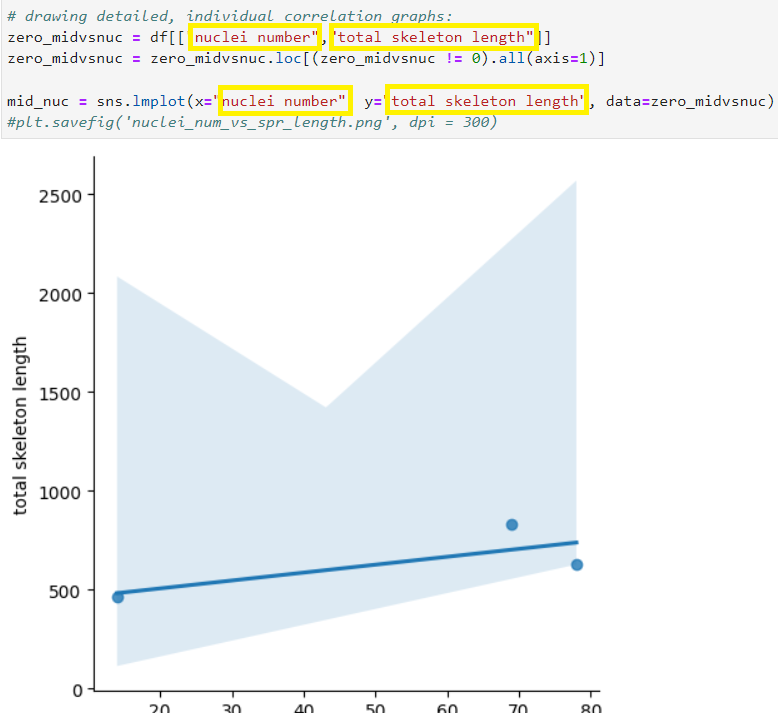


**7.** Next section draws bar graphs. One way of using this part is grouping your data in your excel file. For example, below we show a sample excel document and we added a column to group our data into untreated, 1ng and 10ng. So, we have three groups.


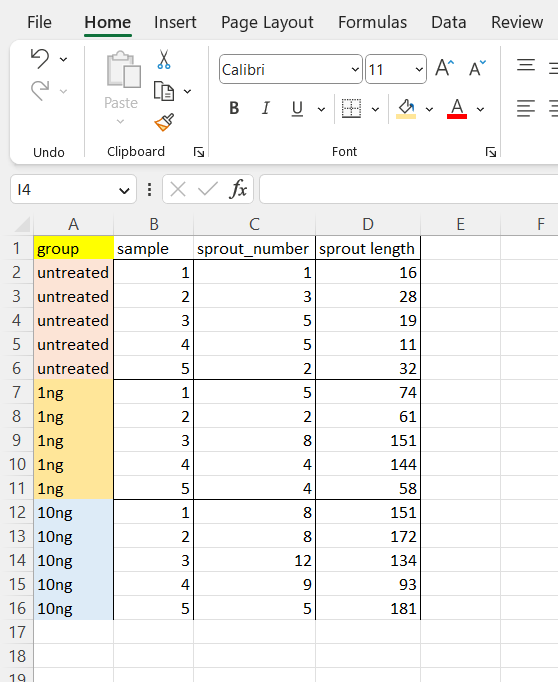


If we had used this excel for defining the path in step 2, then, running the section would give a bar graph with statistics:


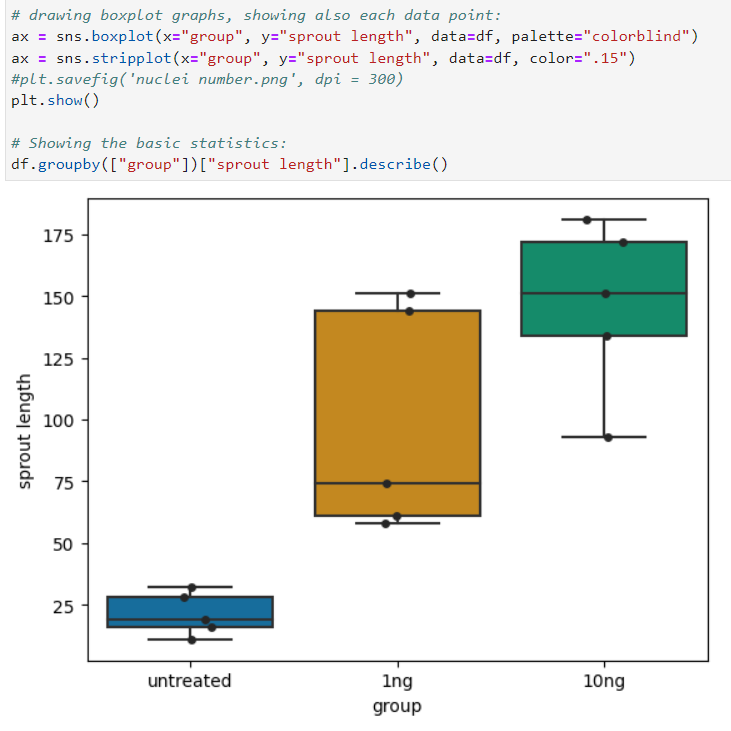


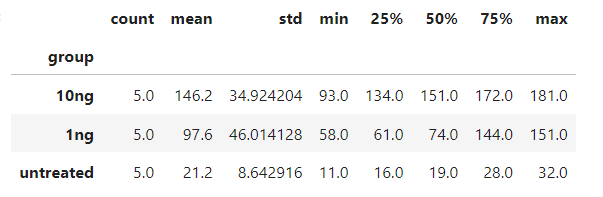


**8.** The last section is an example of Kruskal Wallis test. Here we tested sprout length in our grouped excel data shown above in step 7.


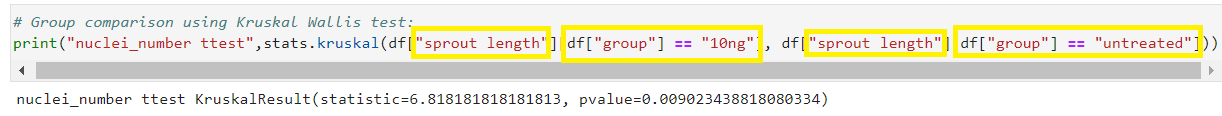
You can use other statistical tests: https://docs.scipy.org/doc/scipy/reference/stats.html
